# Subnanomolar Affinity and Selective Antagonism at α7 Nicotinic Receptor by Combined Modifications of 2-Triethylammonium Ethyl Ether of 4-Stilbenol (MG624)

**DOI:** 10.1021/acs.jmedchem.2c01256

**Published:** 2022-12-16

**Authors:** Francesco Bavo, Marco Pallavicini, Susanna Pucci, Rebecca Appiani, Alessandro Giraudo, Hyoungil Oh, Dana L. Kneisley, Brek Eaton, Linda Lucero, Cecilia Gotti, Francesco Clementi, Paul Whiteaker, Cristiano Bolchi

**Affiliations:** † Dipartimento di Scienze Farmaceutiche, Università degli Studi di Milano, via Mangiagalli 25, I-20133 Milano, Italy; ‡ Department of Drug Design and Pharmacology, University of Copenhagen, DK-2100 Copenhagen, Denmark; § Institute of Neuroscience, CNR, via Vanvitelli 32, I-20129 Milano, Italy; ∥ NeuroMi Milan Center for Neuroscience, University of Milano Bicocca, piazza Ateneo Nuovo 1, I-20126 Milano, Italy; ⊥ Division of Neurobiology, Barrow Neurological Institute, Phoenix, Arizona 85013, United States; # Department of Pharmacology and Toxicology, Medical College of Virginia Campus, Virginia Commonwealth University, Richmond, Virginia 23298, United States

## Abstract

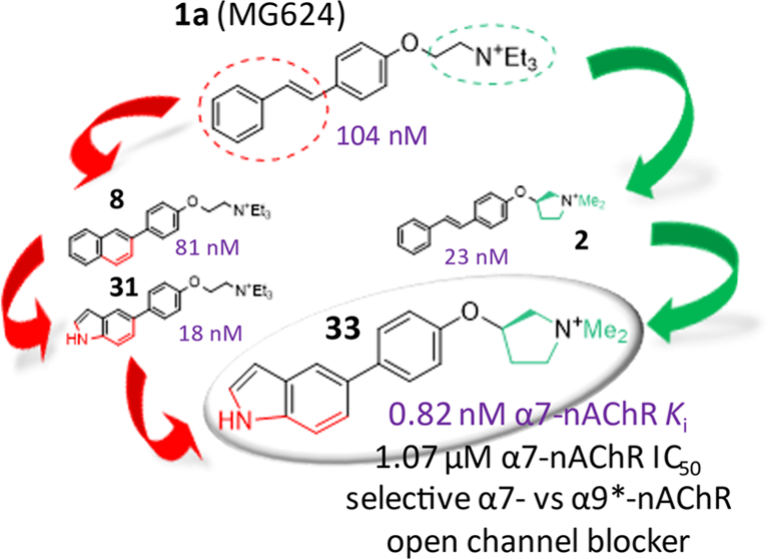

Modifications of the cationic head and the ethylene linker of 2-(triethylammonium)ethyl ether of 4-stilbenol (MG624) have been proved to produce selective α9*-nAChR antagonism devoid of any effect on the α7-subtype. Here, single structural changes at the styryl portion of MG624 lead to prevailing α7-nAChR antagonism without abolishing α9*-nAChR antagonism. Nevertheless, rigidification of the styryl into an aromatic bicycle, better if including a H-bond donor NH, such as 5-indolyl (**31**), resulted in higher and more selective α7-nAChR affinity. Hybridization of this modification with the constraint of the 2-triethylammoniumethyloxy portion into (*R*)-*N,N*-dimethyl-3-pyrrolidiniumoxy substructure, previously reported as the best modification for the α7-nAChR affinity of MG624 (**2**), was a winning strategy. The resulting hybrid **33** had a subnanomolar α7-nAChR affinity and was a potent and selective α7-nAChR antagonist, producing at the α7-, but not at the α9*-nAChR, a profound loss of subsequent ACh function.

## Introduction

The triethylammonium ethyl ether of 4-stilbenol (**1a**, MG624) has returned to the fore in very recent years after being reported in the 1950s as a ganglioplegic agent with very weak antimuscarinic activity and no activity on the neuromuscular junction^1,2^ and first characterized in 1998 as an antagonist of the homopentameric α7 nicotinic acetylcholine receptors (nAChRs) with moderate and high selectivity, respectively, over the β4- and the β2-containing nAChRs.^3^ An expanded knowledge of biochemistry, molecular pharmacology, and physiology of nAChR subtypes, along with a number of structure–activity relationship (SAR) studies, has allowed a fuller understanding of MG624′s pharmacological profile and its multifaceted potential as a therapeutic hit.^4−6^ Starting from the proven ability of nicotine to promote growth and metastasis of lung tumors by acting on α7- and α9α10-nAChRs, we have initially demonstrated that **1a** blocks these proproliferative effects on adenocarcinoma cells expressing such nAChRs.^4^ We have enlarged the investigation to glioblastoma and to analogues of **1a** with elongated O–N alkylene linker, further confirming the antitumor activity and finding that it is greatly advantaged by ethylene bridge lengthening, which generally corresponds to the increasing potency of α7- and α9α10-nAChR antagonism.^5^

**Chart 1:**
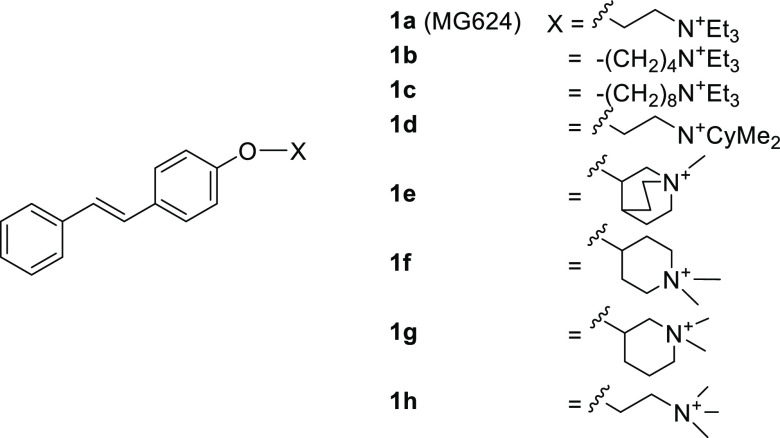
**1a** (MG624) and its Analogues Modified at the Ammonium Ethyl Residue

A deeper pharmacological and functional characterization of **1a** and its two analogues with tetramethylene and octamethylene O–N linker (**1b** and **1c**, respectively; Chart 1) led us to conclude that, at the α7-nAChRs, they behave as a very weak partial agonist (**1a**), a silent agonist (**1b**), and a full antagonist (**1c**) and that their antiproliferative and cytotoxic effects are not only due to the action on nAChRs.^6^ Other non-nicotinic intracellular mechanisms are involved, such as the reduction of the production of mitochondrial and glycolytic adenosine triphosphate (ATP),^5,6^ and further studies are needed to understand whether they are independent or cooperative with nicotinic antagonism. Leaving aside the multiple and incompletely defined mechanisms underlying antiproliferative effects (which are therefore hard to interpret), we returned to the electrophysiological assessment of α7- and α9α10-nAChR subtypes and a systematic SAR study. We have very recently reported a series of analogues of **1a** modified at the ammonium head or at the two-carbon O–N linker.^7^ Some of these modifications, detrimental to the α7-nAChR affinity, such as the inclusion of the linker in six-membered nitrogen heterocycles (**1e**, **1f**, and **1g**; Chart 1) or oversized increase or decrease of the ammonium head volume (**1d** and **1h**, respectively; Chart 1), led to selective antagonists of human α9α10-nAChR, devoid of any antagonist activity at the α7-nAChR and showing partial agonism at high supramicromolar concentrations. As noted in our recent publication, their selective α9α10-nAChR antagonist activity appeared to consist of opening and rapidly engaging the channel and then blocking it in an open but nonconducting state. These observations are compatible with an open-channel block mechanism,^7^ although we emphasize that a definitive demonstration of such a mechanism would require extensive further testing (e.g., competition and voltage-dependence experiments). Among these selective α9α10-nAChR antagonists, the cyclohexyldimethylammonium analogue **1d** (Chart 1) stands out for having no α7-nAChR agonist or antagonist effect and very low affinity for the ganglionic α3β4 nicotinic subtype, thus proposing itself as an invaluable tool to define the therapeutic potential of the α9α10-nAChR antagonism.^7^

**Chart 2:**
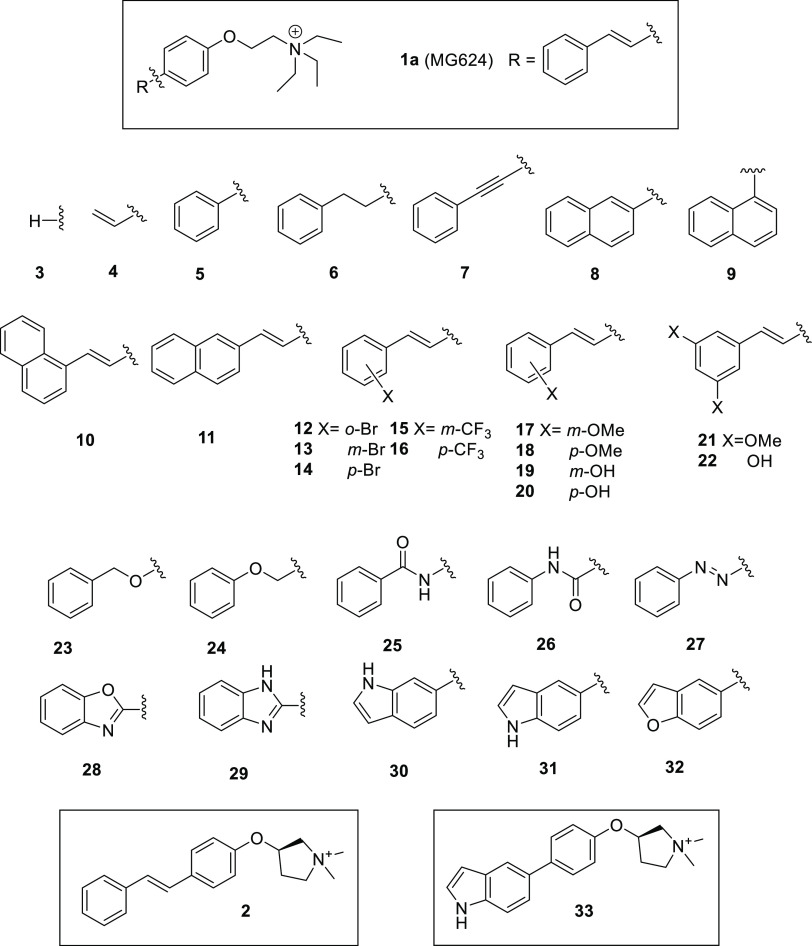
**1a** (MG624), its Analogues Modified at the Styryl Portion (**3**–**32**), its Previously Reported Analogue Modified at the Ammonium Ethyl Residue (**2**), and its Analogue Modified at Both the Substructures (**33**)

As a second part of the SAR investigation on **1a**, we considered modifications at its stilbene scaffold, more specifically at the styryl portion, which represents the distal part of such scaffold and whose modifications were expected to be highly influential, as evidenced by the present results, on the interaction with the α7- and α9α10-nAChR subtypes. Here, we report the synthesis and the biological evaluation of compounds **3**–**33** (Chart 2), in which (a) the styryl residue of **1a** is totally or partially abolished (**3**–**5**), made linear (**7**), derigidified (**6**), or further rigidified (**8** and **9**) also with phenyl bioisosteric replacement (**30**–**32**), decorated at phenyl (**12**–**22**) or benzo-condensed (**10** and **11**), or modified at the vinylene portion by the introduction of heteroatoms and cyclization (**23**–**29**) and (b) the two most productive modifications of **1a** in terms of the α7-nAChR affinity of this series and of the previous one,^7^ respectively, represented by the indolyl analogue **31** and the stilbenoxypyrrolidine **2** (Chart 2), are combined to give hybrid **33**. The biological evaluation was performed similarly to that for previously reported analogues of **1a** modified at the ammonium ethyl residue.^7^ First, an extensive determination of the nAChR subtype binding affinities was performed, followed by the functional screening of a large selection of compounds for α7- and α9α10-nAChR antagonisms and then more detailed tests on a few best hits to further study the mechanism of the antagonist activity at the two receptor subtypes.

## Results

### Chemistry

**Scheme 1:**
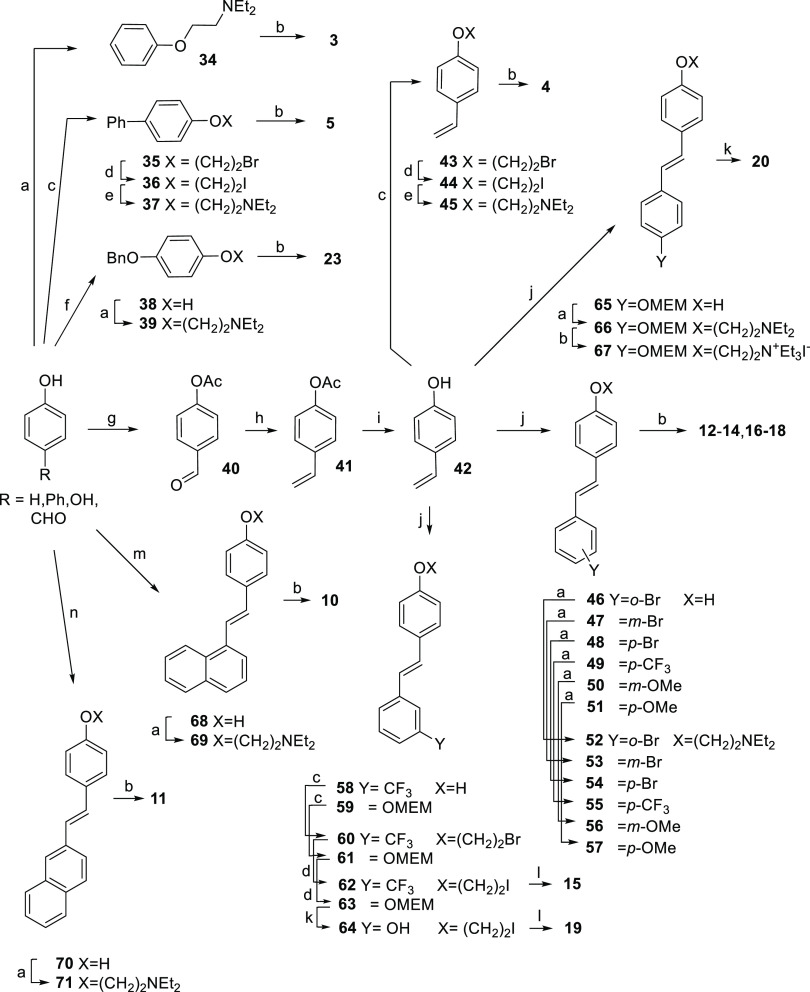
Reagents and Conditions (a) 2-Chloro-*N*,*N*-diethylethylamine hydrochloride, K_2_CO_3_, KI, acetone or methyl ethyl ketone, reflux; (b) iodoethane in 1,2-dichloroethane, rt for **3**, **23**; dichloromethane (DCM), reflux for **4**, **5**; neat, reflux for **17**; EtOH, 70 °C for **10**, **11**; tetrahydrofuran (THF), reflux for **12**–**14**, **16**, **18**, **67**; (c) 1,2-dibromoethane, K_2_CO_3_, KI, methyl ethyl ketone, reflux; (d) NaI, acetone, reflux; (e) diethylamine, toluene, 60 °C; (f) benzyl bromide, K_2_CO_3_, acetone, reflux; (g) acetic anhydride, pyridine, rt; (h) methyltriphenylphosphonium bromide, K_2_CO_3_, THF, reflux; (i) 5 M NaOH, THF, 0 °C; (j) appropriate aryl iodide, Pd(OAc)_2_, triethylamine, CH_3_CN, reflux; (k) 1.25 M HCl in MeOH, reflux; (l) triethylamine, toluene, reflux; (m) 1-naphthylmethyltriphenylphosphonium chloride, sodium, EtOH, 10 °C to rt; and (n) 2-naphthylmethyltriphenylphosphonium bromide, sodium, EtOH, 10 °C to rt.

Compounds **3**–**5**, **10**–**20**, and **23** were synthesized from phenol (compound **3**), 4-phenylphenol (compound **5**), hydroquinone (compound **23**), and *p*-hydroxybenzaldehyde (compounds **4** and **10**–**20**) according to Scheme 1.

Phenol was *O*-alkylated with diethylaminoethyl chloride to give **34**, which was quaternarized to **3** with ethyl iodide.

To obtain the *p*-vinylphenyl ether **4**, *p*-hydroxybenzaldehyde was acetylated (**40**), submitted to Wittig olefination with methylenetriphenylphosphorane (**41**), desacetylated (**42**), *O*-alkylated with 1,2-dibromoethane (**43**), converted into the iodoethyl ether **44**, and then reacted with diethylamine to give **45**, which was quaternarized with ethyl iodide (**4**). Starting from 4-phenylphenol, these last four steps (*O*-bromoethylation, bromine/iodine exchange, diethylamine reaction, quaternarization) led to **5**.

Intermediate **42** was also used to synthesize compounds **12**–**20**. The three positional isomers **12**–**14** were prepared from **42** by coupling with 2-bromo-, 3-bromo-, and 4-bromoiodobenzene, respectively, followed by etherification of phenol with diethylaminoethyl chloride and quaternarization with iodoethane. By the same steps, but using 4-trifluoromethyl-, 3-methoxy-, and 4-methoxyiodobenzene, respectively, we synthesized compounds **16**–**18**. The synthesis of **15** started from 3-trifluoromethyliodobenzene, which was coupled with **42**. The resulting intermediate **58** was *O*-bromoethylated with 1,2-dibromoethane (**60**), converted into the 2-iodoethyl analogue **62**, and then reacted with triethylamine to give **15**. The two positional isomers **19** and **20** were prepared by coupling **42** with MEM-protected 3-iodophenol yielding **59** and with 4-iodophenol yielding **65**. For the synthesis of **19**, the subsequent steps were *O*-bromoethylation (**61**), bromine/iodine exchange (**63**), MEM deprotection (**64**), and reaction with triethylamine. For the synthesis of **20**, the *O-*MEM intermediate **65** was reacted with diethylaminoethyl chloride (**66**), quaternarized with iodoethane (**67**), and MEM-deprotected.

The olefination of *p*-hydroxybenzaldehyde with 1-naphthylmethylenetriphenylphosphorane yielded intermediate **68**, which was treated with diethylaminoethyl chloride to give the tertiary amine **69** and then converted into **10** by treatment with ethyl iodide. The olefination of *p*-hydroxybenzaldehyde with 2-naphthylmethylenetriphenylphosphorane provided intermediate **70** and its cis isomer, which were separated by chromatography. Successive etherification of **70** with diethylaminoethyl chloride and quaternarization with iodoethane gave **11**.

Compound **23** was obtained from hydroquinone by etherification of one hydroxyl with benzyl bromide (**38**) and of the other with diethylaminoethyl chloride (**39**), followed by quaternarization of the tertiary amine **39** with iodoethane.

**Scheme 2:**
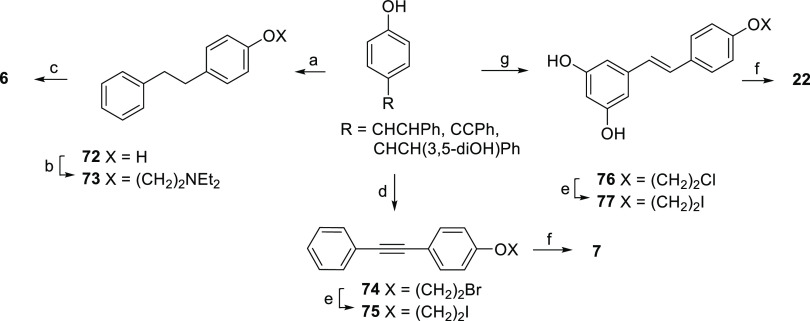
Reagents and Conditions (a) H_2_, Pd/C, MeOH, rt; (b) 2-chloro-*N*,*N*-diethylethylamine hydrochloride, K_2_CO_3_, KI, methyl ethyl ketone, reflux; (c) iodoethane, toluene, 90 °C; (d) 1,2-dibromoethane, K_2_CO_3_, KI, methyl ethyl ketone, reflux; (e) NaI, acetone, reflux; (f) triethylamine, toluene, rt for **7**, reflux for **22**; and (g) 1-bromo-2-chloroethane, K_2_CO_3_, *N*,*N*-dimethylformamide (DMF), 60 °C.

Compounds **6**, **7**, and **22** were synthesized from *trans*-4-stilbenol, 4-(2-phenylethynyl)phenol, and resveratrol, respectively, according to Scheme 2. Stilbenol was hydrogenated to 4-(2-phenylethyl)phenol (**72**), *O*-alkylated with diethylaminoethyl chloride (**73**), and quaternarized with iodoethane to give **6**. 4-(2-Phenylethynyl)phenol was *O*-alkylated with 1,2-dibromoethane (**74**) and, after bromine/iodine exchange (**75**), converted to **7** by reaction with triethylamine. Resveratrol was chloroethylated at the 4′-hydroxyl with 1-bromo-2-chloroethane (**76**) and, after chlorine/iodine exchange (**77**), converted to **22** by reaction with triethylamine.

**Scheme 3:**
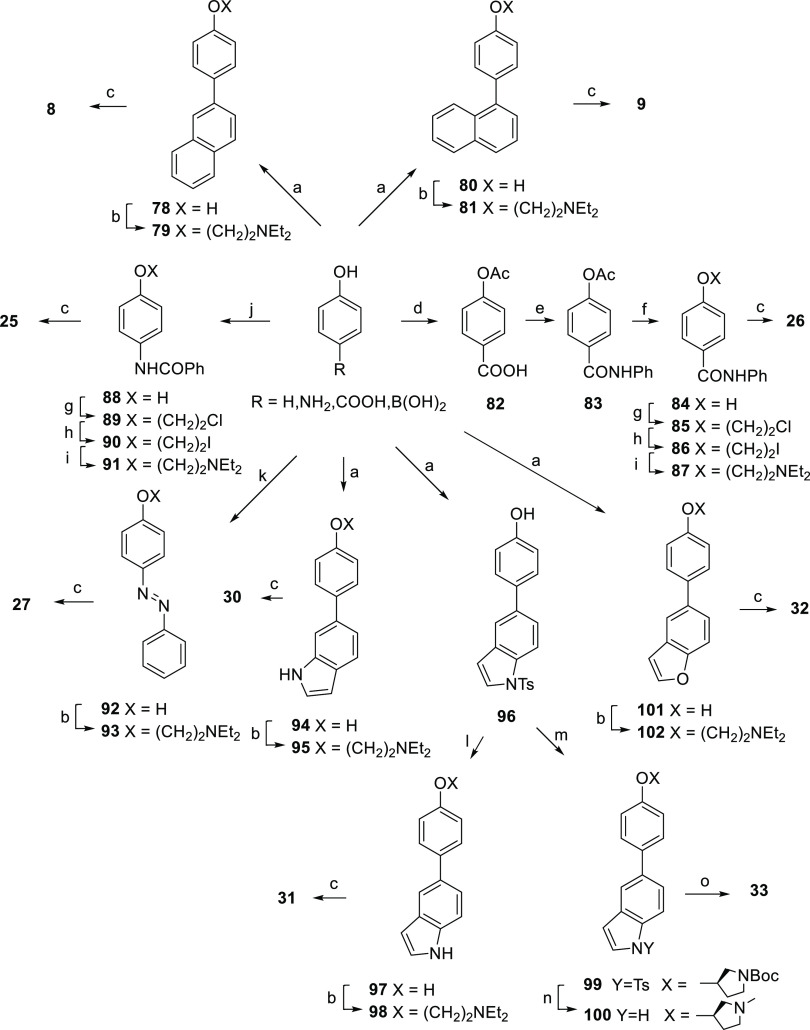
Reagents and Conditions (a) Appropriate aryl bromide, Pd(PPh_3_)_4_, tetrabutyl ammonium bromide (TBAB), EtOH/2 M_aq_ Na_2_CO_3_, 1,2-dimethoxyethane or EtOH/toluene, reflux; (b) 2-chloro-*N*,*N*-diethylethylamine hydrochloride, K_2_CO_3_, KI, methyl ethyl ketone, reflux; (c) iodoethane: neat, reflux for **8**, **9**, **25**, **26**; DCM, rt for **27**; THF, reflux for **30**–**32**; (d) Ac_2_O, H_2_SO_4_, 80 °C; (e) 1° step: ClCOCOCl, DCM, DMF, rt; 2° step: aniline, DCM, rt; (f) 1 M NaOH, MeOH, rt; (g) 1-chloro-2-bromoethane, Cs_2_CO_3_, DMF, 60 °C; (h) NaI, acetone, reflux; (i) diethylamine, reflux; (j) benzoic anhydride, sodium octyl sulfate, H_2_O, CH_3_CN, rt; (k) 1° step: aniline, NaNO_2_, 37% HCl, H_2_O, 0 °C to rt; 2° step: NaHCO_3_, rt; (l) KOH, MeOH, reflux; (m) *tert*-butyl (*S*)-3-hydroxypyrrolidine-1-carboxylate, PPh_3_, diisopropyl azodicarboxylate (DIAD), THF, −10 °C to reflux; (n) LiAlH_4_, THF, −10 °C to reflux; and (o) iodomethane, THF, 40 °C.

Scheme 3 shows the syntheses of compounds **8**, **9**, **25**–**27**, and **30**–**33**. 4-Hydroxyphenyl boronic acid was coupled with 2-bromo- and 1-bromonaphthalene and the resulting intermediates, **78** and **80**, respectively, were *O*-alkylated with diethylaminoethyl chloride (**79** and **81**) and quaternarized to **8** and **9**, respectively, with ethyl iodide. By the same reaction sequence, we synthesized the final compounds **30** and **32** from 4-hydroxyphenyl boronic acid using 6-bromoindole and 5-bromobenzofuran respectively. For the synthesis of **31**, 4-hydroxyphenyl boronic was coupled with *N*-tosyl-5-bromoindole and the resulting intermediate tosyl amide **96** was hydrolyzed to **97**, *O*-alkylated with diethylaminoethyl chloride (**98**), and converted to **31** with iodoethane. Intermediate **96** was coupled, by the Mitsunobu reaction, with (*R*)-*N*-boc-3-hydroxypyrrolidine to give **99**. Subsequent reduction with LiAlH_4_ provided the *N*-methyl pyrrolidine **100**, which was converted to **33** by treatment with iodomethane. The preparation of **25** and **26** was accomplished from 4-benzamidophenol (**88**) and 4-hydroxybenzanilide (**84**), respectively, through the same sequence of reactions: *O*-chloroethylation (**89** and **85**), chlorine/iodine exchange (**90** and **86**), reaction with diethylamine (**91** and **87**), and quaternarization with iodoethane (**25** and **26**). 4-Benzamidophenol (**88**) was prepared from 4-aminophenol, while 4-hydroxybenzanilide (**84**) was prepared from *p*-salicylic acid by acetylation (**82**), conversion into *p-*hydroxybenzoyl chloride, and reaction with aniline (**83**) and desacetylation. For the synthesis of **27**, phenol was coupled with benzenediazonium salt, generated in situ from aniline, and the obtained compound **92** was reacted with diethylaminoethyl chloride (**93**) and quaternarized to **27** with iodoethane.

**Scheme 4:**
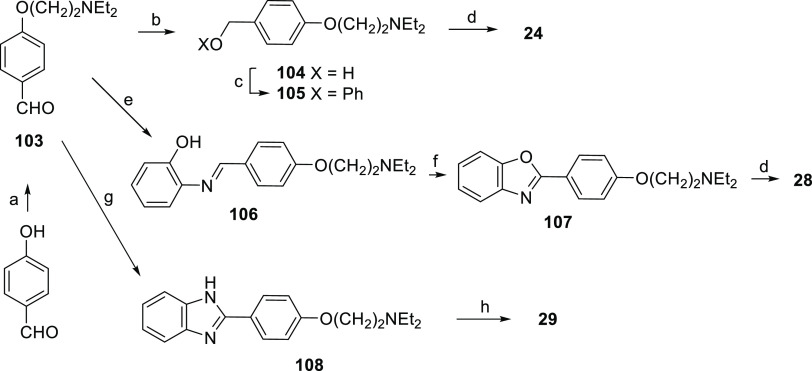
Reagents and Conditions (a) 2-Chloro-*N*,*N*-diethylethylamine hydrochloride, K_2_CO_3_, KI, methyl ethyl ketone, reflux; (b) NaBH_4_, MeOH, rt; (c) phenol, PPh_3_, DEAD, THF 0 °C to rt; (d) iodoethane, rt; (e) *o*-aminophenol, EtOH, reflux; (f) Pb(OAc)_4_, EtOH, reflux; (g) *o*-phenylenediamine, Pb(OAc)_4_, EtOH, reflux; and (h) iodoethane, 1,2-dichloroethane, rt.

Scheme 4 shows the syntheses of compounds **24**, **28**, and **29**. 4-Hydroxybenzaldehyde was etherified with diethylaminoethyl chloride to intermediate **103**, reduced to benzyl alcohol **104**, transformed into phenyl ether **105** by the Mitsunobu reaction with phenol, and quaternarized to **24** with ethyl iodide. Intermediate **103** was also used to synthesize the benzoxazole nucleus of **28** and the benzimidazole nucleus of **29**. The addition–elimination reaction of **103** with *o*-aminophenol provided the Schiff base **106**, which was transformed into **107** by oxidative cyclization and then quaternarized to **28** with ethyl iodide. The benzimidazole intermediate **108** was directly obtained from **103** by reaction with 2-aminoaniline in the presence of lead tetraacetate. Final quaternarization with ethyl iodide provided **29**.

### Biology

#### Binding Studies

The binding affinities (*K*_i_) of all of the compounds were determined by competition binding experiments on the α7 human subtype, transiently expressed in the SH-SY5Y neuroblastoma cells,^5^ and the results are shown in Table 1. With the exception of a few compounds that had a modest affinity for α7-nAChR, competitive binding affinity was also assessed at the human α3β4-nAChR subtype stably transfected in SH-EP1 cells^8^ and only select compounds were also tested on the human α4β2-nAChR subtype stably transfected in HEK 293 cells (a generous gift from Dr. Jon Lindstrom^9^).

**Table 1:** Affinity (*K*i in μM) of Compounds for the Human α7, α3β4, and α4β2-nAChR Subtypes

	α7-nAChR [^125^I]-αBgtx *K*_i_ (μM)	α3β4-nAChR [^3^H]-Epi *K*_i_ (μM)	α4β2-nAChR [^3^H]-Epi *K*_i_ (μM)		α7-nAChR [^125^I]-αBgtx *K*_i_ (μM)	α3β4-nAChR [^3^H]-Epi *K*_i_ (μM)	α4β2-nAChR [^3^H]-Epi *K*_i_ (μM)
**1a**	0.104 (0.55–0.202)	0.433 (0.227–0.823)	5.7 (3–10.6)	**18**	0.353 (0.146–0.853)	0.187 (0.090–0.388)	nd
**2**	0.023 (0.09–0.055)	2.700 (1.800–4.200)	9.3 (2.9–30)	**19**	0.573 (3.79–0.866)	0.998 (0.714–1.280)	10.1 (5.5–18.5)
**3**	27 (17.6–44.3)	10 (6.2–19)	19 (10–37)	**20**	0.342 (225–0.520)	0.873 (0.484–1.575)	9.82 (5.8–16.6)
**4**	0.285 (0.110–0.379)	1.070 (0.618–1.800)	24 (6.5–91)	**21**	0.189 (0.096–0.393)	0.676 (0.406–1.125)	1.2 (0.49–3.3)
**5**	0.129 (0.048–0.349)	0.440 (0.247–0.784)	11.8 (7–37)	**22**	0.242 (0.139–0.422)	0.793 (0.579–1.084)	7.24 (4.2–12.4)
**6**	1.646 (0.643–4.208)	1.249 (0.590–2.643)	nd	**23**	1.010 (0.380–2.670)	4.700 (3.400–6.400)	7.5 (10–37)
**7**	0.664 (0.416–1.061)	0.912 (0.693–1.200)	19.4 (3.5–105)	**24**	2.600 (1.020–6.800)	nd	nd
**8**	0.081 (0.046–0.145)	0.458 (0.191–1.000)	1.8 (0.69–4.7)	**25**	0.862 (0.522–1.400)	1.070 (0.208–5.500)	4.5 (0.77–27)
**9**	3.107 (1.532–6.299)	0.097 (0.418–2.263)	nd	**26**	0.250 (0.110–0.623)	0.113 (0.026–0.488)	23 (3.9–100)
**10**	1.347 (0.488–3.713)	0.501 (0.255–0.985)	nd	**27**	0.526 (0.310–0.805)	nd	nd
**11**	1.004 (0.425–2.374)	0.425 (0.192–0.985)	nd	**28**	0.166 (0.071–0.391)	0.653 (0.316–1.346)	nd
**12**	0.525 (0.293–0.940)	nd	nd	**29**	0.0336 (0.016–0.072)	0.345 (0.152–0.781)	nd
**13**	1.036 (0.539–1.990)	nd	nd	**30**	0.184 (0.091–0.374)	0.174 (0.067–0.453)	nd
**14**	0.723 (0.421–1.240)	nd	nd	**31**	0.0187 (0.0086–0.0402)	0.177 (0.078–0.403)	nd
**15**	1.525 (0.987–2.358)	1.411 (0.926–2.150)	21.8 (17–28)	**32**	0.450 (0.198–1.022)	0.343 (0.155–0.759)	nd
**16**	1.334 (0.734–2.423)	0.874 (0.597–1.280)	10.4 (4–26.7)	**33**	0.00082 (0.00065–0.00123)	0.365 (0.274–0.485)	5.1 (3.1–8.4)
**17**	0.621 (0.415–0.921)	nd	nd				

Heterologously expressed human receptors were used. α4β2 and α3β4-nAChR subtypes were expressed in HEK 293 or SH-EP1 cells, respectively; human α7-nAChR was expressed in SH-SY5Y human neuroblastoma cells. Binding was determined using as ligand [^3^H]epibatidine for α4β2- and α3β4-nAChR subtypes and [^125^I] α-bungarotoxin for the α7-subtype. Saturation and competition binding data were evaluated by one-site competitive binding curve-fitting procedures using GraphPad Prism version 6 (GraphPad Software, CA). In the saturation binding assay, the maximum specific binding (*B*_max_) and the equilibrium binding constant (*K*_d_) values were calculated using one-site—specific binding with the Hill slope—model. Inhibition constants (*K*_i_) were obtained by fitting three independent competition binding experiments, each performed in duplicate for each compound on each subtype and were estimated by reference to the *K*_d_ of the radioligand, obtained in separate saturation binding experiments, according to the Cheng–Prusoff equation and expressed in micromolar. The numbers in parentheses of *K*_i_ values represent the confidence interval of the value.

We found that, among the compounds modified by structural simplification or rigidification of the styryl residue (**3**–**9)**, only compounds **5** and **8** had an α7-nAChR *K*_i_ value close to that of the parent compound **1a** (*K*_i_ = 104 nM) and maintained a modest α7- vs α3β4-nAChR and a high α7- vs α4β2-nAChR selectivity.

The second set of analogues of **1a**, those decorated at the distal phenyl by substituents or an additional condensed benzene (compounds **10**–**22**), showed both a lower affinity for α7-nAChR and reduced selectivity over α3β4-nAChR (where measured).

The same outcome was seen across the third set of analogues (compounds **23**–**27**), those with an ether, an amide, or a diazo linker in place of vinylene. However, replacement of the vinylene linker with an imino linker locked into oxazole or imidazole condensed with the distal phenyl (compounds **28** and **29**) led, in the case of benzimidazole **29**, to an improved α7-nAChR affinity (*K*_i_ = 33.6 nM) and α7- vs α3β4-nAChR selectivity (10.3 ratio) compared to **1a (***K*_i_ = 104 nM; α7- vs α3β4-nAChR selectivity = 4.2). Among the last set of compounds (**30**–**32**), formally derived from **8** by replacement of 2-naphthyl with 5- or 6-indolyl or 5-benzoxazolyl, analogous results were obtained for indole **31** (18.7 nM *K*_i_ and 9.5 ratio).

Finally, we determined for compound **33**, a hybrid between compounds **31** and **2**, very high α7-nAChR affinity (0.82 nM *K*_i_), and α7- vs α3β4- and α4β2-nAChR selectivities (445 and 6200 ratios, respectively). Compound **2** is a previously reported analogue of **1a**,^7^ modified at the O–N linker and endowed with high α7-nAChR affinity (23 nM *K*_i_) and α7- vs α3β4- and α4β2-nAChR selectivities (117 and 404 ratios, respectively).

The binding affinities of the compounds for the α3β4- and α7-nAChR subtypes were generally similar or moderately different (≤10-fold ratio), except for the above-mentioned compounds **2** and **33** that had >400-fold preference for α7- over α3β4-nAChR and for compound **9**, which had ≈30-fold higher affinity for the α3β4- than for the α7-nAChR. Approximately half of the compounds were also tested for their affinity for the α4β2-nAChR, and we determined that all had low affinity (*K*_i_ > 1.2 μM) including compounds **1a**, **2**, **5**, **8**, and **33** that we have determined to have high α7-nAChR affinity.

#### *In Vitro* Functional Activity on α7 and α9α10-nAChR Subtypes

Compound **1a** was earlier shown to be an antagonist of chicken α7-nAChR expressed in *Xenopus laevis* oocytes (IC_50_ = 109 nM) and, more recently, at human α7 and α9α10-nAChR expressed in *X. laevis* oocytes (IC_50_ = 41 and 10 nM, at the respective subtypes).^3,5^

**Figure 1:**
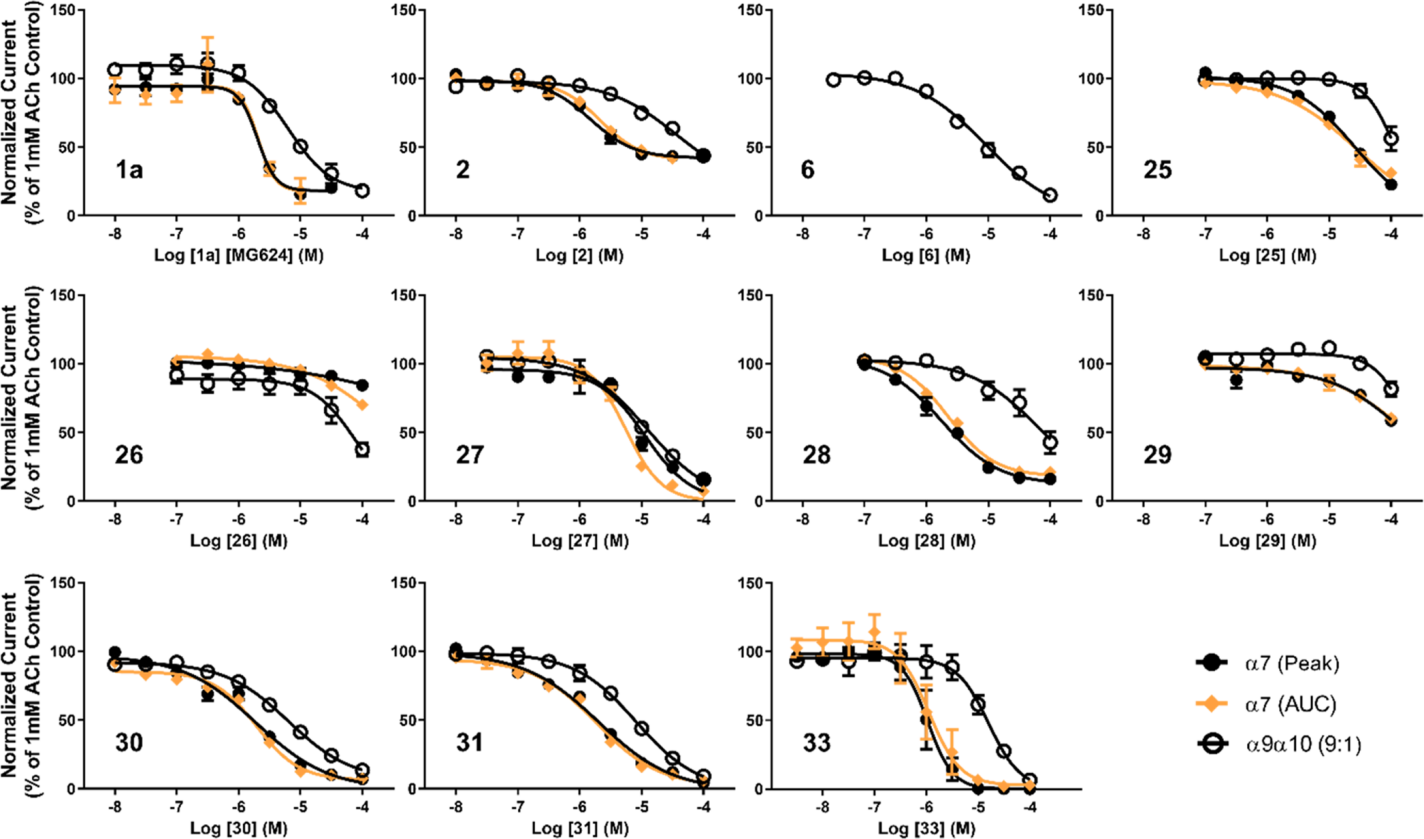
Inhibition concentration response profiles of test compounds at α7- or α9α10-nAChR subtypes. mRNA encoding human α7-nAChR subunit was coinjected in *X. laevis* oocytes along with mRNA for NACHO (in enhanced expression of α7-nAChR; ● or ⧫). Separate batches of oocytes were injected at a 9:1 ratio with mRNA encoding human α9- and α10-nAChR subunits, respectively (open circles). In both cases, the function was tested 1 week after injection, employing two-electrode voltage clamp electrophysiology. Initial stimulations were ACh-only (1 mM, 1 s stimulation, 60 s wash between stimulations, five repeats). These initial stimulations were used to confirm that agonist-alone responses were stabilized, and to provide a positive control, before test compounds were applied. Test compounds were coapplied with ACh stimulations (same 1 mM ACh concentration, 1 s application time, and 60 s wash between applications, as was used for the initial ACh-only stimulations). Concentrations of test compounds were increased from the lowest shown to a maximum of 100 μM in half-log steps. For α7-nAChR, responses were measured in two different ways (as peak currents (●) or as area under the curve (⧫). In all cases, responses when test compounds were coapplied were normalized to the mean of the magnitude of the final two positive control responses that preceded the introduction of the test compound. Each point is the mean ± standard error of mean (S.E.M.) of five to six responses, with each response being collected from an individual oocyte. Error bars are included for all points but are not visible where the size of the point exceeds that of the corresponding error bars. Even coapplication of compound **6** at 100 μM produced no inhibition of the α7-nAChR function; the resulting data have been omitted to increase clarity.

Of the compounds reported in this manuscript, nine (**6**, **25**–**31**, and **33**) were chosen for further testing in functional assays. The selection was driven by the significantly higher α7-nAChR binding potency and selectivity for α7- over α3β4-nAChR binding shown by **29** and **31** in comparison with **1a**, suggesting further rigidification and introduction of a weakly acidic NH in a suitable position as critical modifications of the styryl moiety of **1a**. Therefore, *in vitro* functional tests were extended also to benzamides **25** and **26**, benzoxazole **28**, and indole **30**, in which one or both the above modifications at the styryl moiety are featured. Compound **33** was selected on the basis of its greatly improved (subnanomolar) α7-nAChR affinity and better α7- over α3β4-nAChR selectivity compared to any of the other compounds considered in this study, while the diazo derivative **27** and the phenylethyl analogue **6** were included for the significance of their respective linker modifications. Antagonism of currents activated by 1 mM ACh was determined using *X. laevis* oocytes that expressed human α7- or α9α10-nAChR. Test compounds were coapplied during agonist stimulation. The approach and apparatus were similar to those earlier published for α7-nAChR.^10^ However, in this case, α9α10-nAChR was also tested (from oocytes injected with α9 to α10 cRNAs at a 9:1 ratio). As noted in our recent publication,^7^ the injection of α9-nAChR cRNA alone produces very little function. In contrast, the injection of our chosen ratio of α9 and α10 cRNA (9:1) produced the most function. Use of this α9:α10 cRNA injection ratio will likely produce functional α9α10-nAChR incorporating subunits in two different stoichiometries: (α9)_2_(α10)_3_ and (α9)_3_(α10)_2_.^11^ The just noted increase in function following coinjection of the α10 subunit (compared to that if the α9 subunit cRNA is injected alone) further reassures us that the α9-only-nAChR function will be either minimal or absent under the 9:1 α9:α10 cRNA coinjection condition that we use in this and our previous manuscript. We chose to use the same experimental approaches for the present study to allow comparisons to be made to our recently published data.^7^ Functional responses of α7-nAChR were assessed using both the measurement of peak currents and net charge gated (area under curve or AUC) to determine whether the rapid-desensitizing property of this subtype at high agonist concentrations might alter the IC_50_ values obtained.^12^ The concentration response curves thus obtained are shown in Figure 1, and the IC_50_ values calculated in each case are summarized in Table 2. Also given in Table 2 are IC_50_ values for the lead compound (**1a**) and for **2** (for comparison since, together with **31**, it is the parent compound of **33**). As may be seen, in this experiment, under the conditions applied here, IC_50_ values calculated using either peak current or AUC measurements of the α7-nAChR function were extremely similar. Despite this, it is worth mentioning that the exceptionally rapid kinetics of the α7-nAChR function at high agonist concentrations raise a concern that the coapplication of antagonists may result in inhibition being measured when drug application is incomplete. For this reason, later parts of this study examined the effects of applying the test compounds by themselves, rather than in a coapplication format.

**Table 2:** Inhibition Potency (IC_50_) of Test Compounds at α7- and α9α10-nAChR Determined from Concentration Response Curves Illustrated in Figure 1

	α7 IC_50_ μM (peak current)	α7 IC_50_ μM (AUC)	α9α10 IC_50_ μM
**1a**	1.99 (1.78–2.24)	2.08 (1.10–3.93)	6.68 (5.62–7.76)
**2**	1.49 (1.23–1.78)	2.17 (1.32–3.58)	36.5 (17.0–77.6)
**6**	NA	NA	9.12 (7.76–10.72)
**25**	25.7 (21.9–30.2)	26.9 (20.4–35.5)	115 (41.7–316)
**26**	>100	>100	75.9 (61.7–93.3)
**27**	11.2 (9.77–12.9)	5.54 (4.27–7.18)	15.8 (11.7–21.4)
**28**	1.78 (1.45–2.29)	2.25 (1.78–2.85)	72.4 (31.6–207.0)
**29**	182 (129–257)	197 (112–347)	202 (144–288)
**30**	1.91 (1.48–2.45)	1.95 (1.63–2.34)	6.46 (5.49–7.49)
**31**	2.01 (1.70–2.34)	1.86 (1.22–2.81)	8.13 (6.31–13.2)
**33**	1.07 (0.89–1.29)	1.12 (0.62–2.03)	15.9 (11.8–21.4)

The summary of the test compound antagonist potency (IC_50_ values) is derived using the concentration response curves illustrated in Figure 1. Details of the protocols used are given in the Experimental Section and Figure 1 (legend). Please note that IC_50_ values at α7-nAChR were calculated using both peak current and area under the curve (AUC) approaches. Both approaches yielded similar values for all compounds tested. Confidence intervals (95% values) are provided in parentheses, which represent the 95% confidence interval of the mean value. “NA”, not applicable (i.e., agonist-induced function was not inhibited by the coapplication of the test compound even at 100 μM).

Except for **6**, which had no effect at the α7-subtype, all of the tested compounds were able to inhibit ACh activity at both the subtypes: **27** and **29** with almost identical potency at α7 and α9α10-nAChR, **26** with selectivity toward the α9α10-nAChR, and the remaining **1a**, **2**, **25**, **28**, **30**, **31**, and **33** with higher potency at the α7-nAChR subtype. As shown in Figure 1, some compounds were not able to produce complete inhibition of the nAChR function. In some cases, inhibitory concentration response curves reached a plateau of incomplete antagonism. In others, the maximum test compound concentration of 100 μM was insufficient to produce complete inhibition. However, complete or nearly complete inhibition was observed at both α7- and α9α10-nAChR subtypes for **27**, **30**, **31**, and **33**. Among these four compounds, **33**, the most potent α7-nAChR antagonist of the whole series (1.07 μM IC_50_), showed the highest α7- vs α9α10-nAChR selectivity. Importantly, none of the compounds produced biphasic inhibition of the α9α10-nAChR function. This indicates that in no case do any of the test compounds discriminate between the alternate α9α10-nAChR stoichiometries described in the prior paragraph as likely to be present under the experimental conditions used in this study.

We emphasize here that while sequential applications of test compounds at progressively higher concentrations are common practice, it could result in compounding of effects (in this case, antagonism) produced by previous applications. For this reason, four compounds of special interest were selected to examine their potential intrinsic agonist affinity at α7- and α9α10-nAChR and the ability to affect function induced by a subsequent application of an ACh control response. These were compound **6**, which exerted no inhibition of α7-nAChR but essentially full inhibition of α9α10-nAChR responses, and compounds **2**, **28**, and **33**, all exhibiting the highest and most selective α7-nAChR antagonist activity (∼1 μM IC_50_, 15–40-fold selectivity over α9α10-nAChR IC_50_). As for the preceding experiment, repeated applications of ACh (1 mM) were used to ensure the stability of functional responses and define an agonist positive control response. Subsequently, each compound of interest was applied at a single concentration of 100 μM (no ACh present) to oocytes expressing α9α10-nAChR or, excluding **6**, to oocytes expressing α7-nAChR. The 100 μM concentration was chosen since it matches the final concentration of the test compounds when they were coapplied with ACh in Figure 1, allowing outcomes to be compared directly. The application of a single concentration, in the absence of ACh, addresses the concern stated at the beginning of this paragraph that sequential applications of the test compounds could result in compounding of their effects.

**Figure 2:**
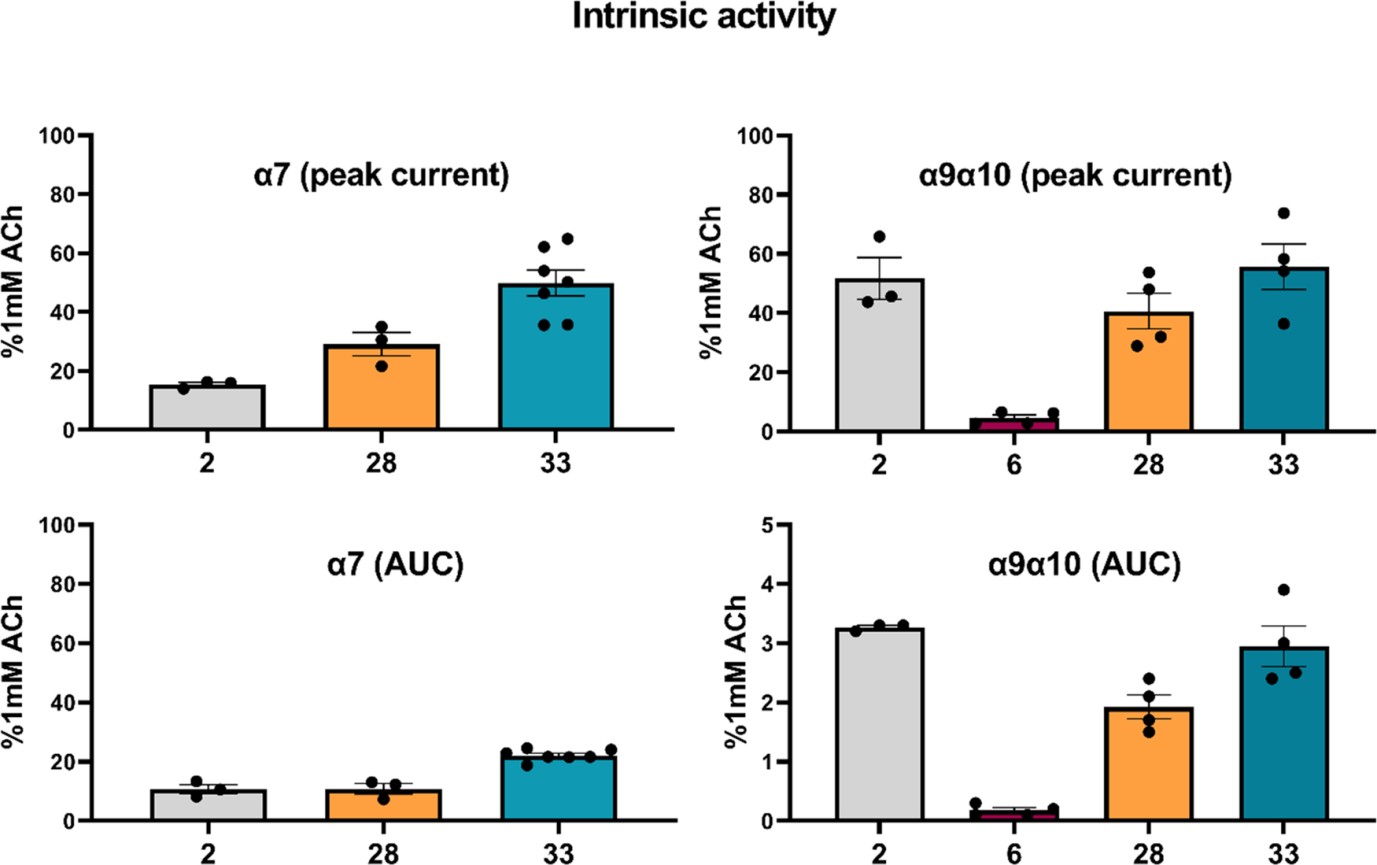
Partial agonism of human α7- or α9α10-nAChRs by compounds **2**, **6**, **28**, or **33** (applied alone). Compounds **2**, **6**, **28**, and **33** were selected (please see the test for criteria) to determine whether they were able to activate human α7- or α9α10-nAChR (intrinsic activity). Two-electrode voltage clamp protocols were similar to those used in Figure 1, including the use of an initial train of ACh (1 mM) control pulses to ensure the stability of responses and collect positive control data for a full agonist. After a further 1 min wash period, compounds of interest were applied for 1 s at 100 μM (the same as the highest concentration applied in Figure 1; in this case, test compounds were applied alone instead of coapplied with ACh). In this case, responses at both α7- and α9α10-nAChR were quantified in terms of both peak currents and AUC. For each individual oocyte, and for each method of quantification, responses when test compounds were coapplied were normalized to the mean of the magnitude of the final two positive control responses that preceded the introduction of the test compound. Each bar represents the mean response collected from three individual oocytes, with error bars representing the S.E.M. Points represent responses from individual oocytes.

Application of an individual 100 μM pulse of any of compounds **2**, **6**, **28**, or **33** resulted in the partial agonism of α9α10-nAChRs (efficacy of 5–55% of ACh control when responses were measured in terms of the peak current). Interestingly, as previously reported for some analogues of **1a** modified at the ammonium ethyl portion,^7^ all of the α9α10-nAChR functional responses induced by the test compounds were shorter-lasting than those evoked by the application of the ACh control. When responses were considered in terms of AUC, this resulted in efficacy being reduced to 0.2–3% of ACh control responses. Similar outcomes were found at α7-nAChR for compounds **2**, **28**, and **33** (15–50% efficacy compared to ACh control). When the intrinsic activity was assessed in terms of AUC, it was reduced somewhat (to 10–22% of the ACh control). This suggests that responses produced at α7-nAChR by the test compound were also somewhat truncated compared to those induced by ACh, albeit to a lesser extent than was seen at α9α10-nAChR. However, compound **6**, which did not affect ligand binding at the α7-subtype also, did not have an intrinsic activity at α7-nAChRs (Figure 2). Of interest, compound **1a** has also been reported recently to be an α7-nAChR partial agonist (response to a 100 μM application noted to be ≈40% of the 200 μM ACh control stimulation).^6^

**Figure 3:**
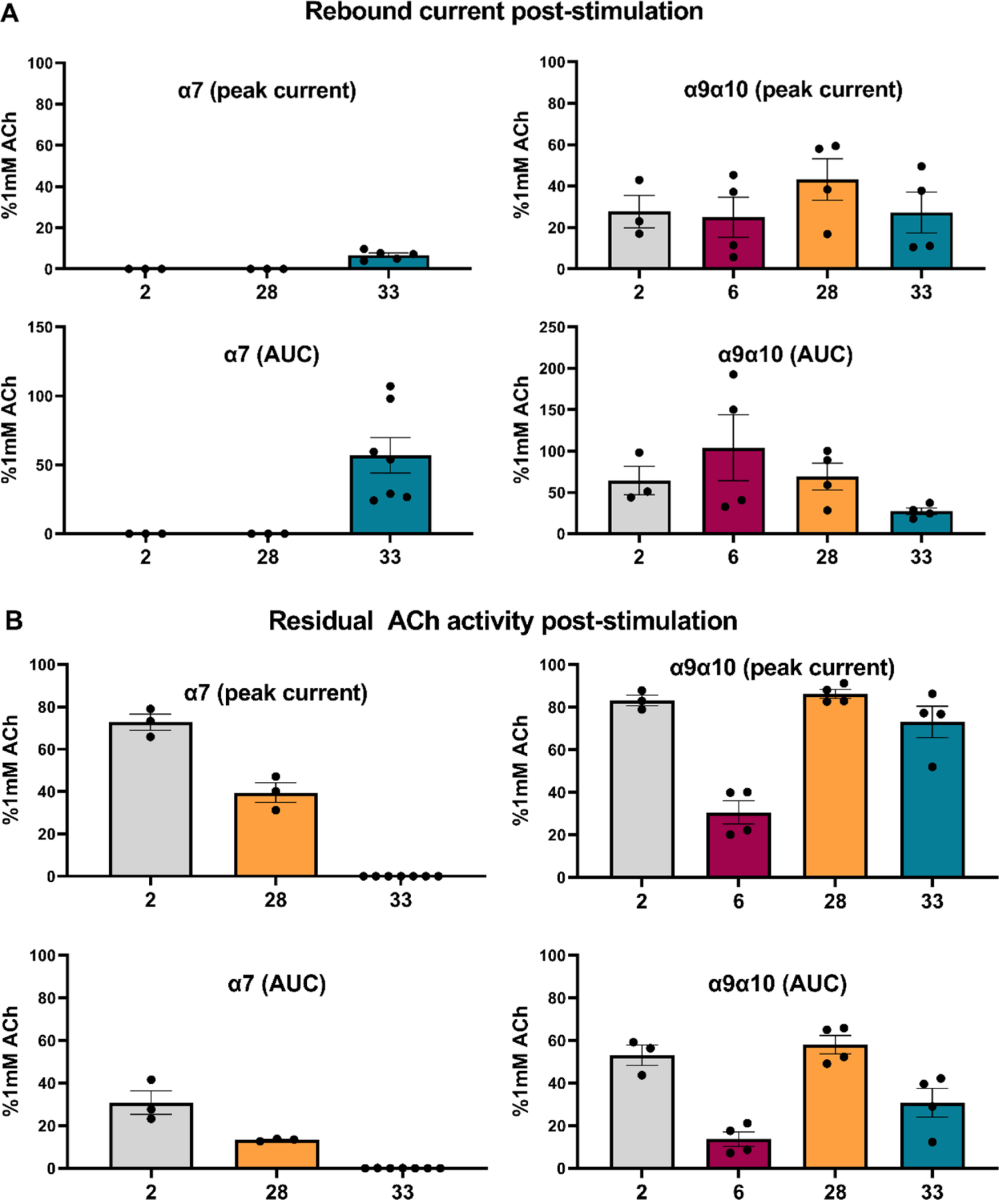
Illustration of rebound current magnitudes and residual ACh activity following the application of compounds of interest. (A) Magnitudes of rebound currents appearing after the cessation of test compound application were recorded from α7- and α9α10-nAChRs. As for Figure 2, responses at both α7- and α9α10-nAChR were quantified in terms of both peak currents and AUC. (B) Residual activation evoked by a final ACh control application (1 mM, 1 s) applied 1 min after stimulation with each compound of interest. In this case, too, responses at both nAChR subtypes were measured as both peak current and AUC and, in each case, normalized to the ACh control responses that preceded the application of the test compound. Each bar represents the mean response collected from three to seven individual oocytes, with error bars representing the S.E.M. Points represent responses from individual oocytes.

Further, at α9α10-nAChR, rebound currents were observed, subsequent to the recovery of the short-duration currents produced in response to the test compound application. These rebound currents lasted longer than initial currents evoked by the test compounds or even preceding the ACh control responses. This phenomenon is illustrated in example traces, shown in Supporting Information Data (pages 20–26). In contrast, at α7-nAChR, only compound **33** evoked a rebound current (example traces are also provided in the same section of Supporting Information Data). Figure 3A illustrates the size of the poststimulation rebound currents evoked by **2**, **6**, **28**, and **33** at α9α10-nAChRs and of **2**, **28**, and **33** at α7-nAChR. In each case, responses are normalized to the size of control responses previously evoked by ACh positive control responses. Responses are again presented in terms of both peak currents and AUC. As may be seen, peak currents attained during these rebound currents following test compound application varied between 25 and 40% of those produced by ACh control stimulations. However, when AUC was considered, rebound currents varied between 27 and 100% of control. This reflects the effects of the relatively slow onset and recovery of the rebound currents when compared to the initial responses to test compound application.

Responses were also measured for a final ACh (1 mM, 1 s) control stimulation, applied 1 min after test compound application to each oocyte. These final ACh control applications produced a response that was reduced (in some cases, much reduced) in amplitude (whether in terms of peak current or AUC) than the initial ACh control applications. In Figure 3B, we illustrate the residual activities induced by these concluding ACh (1 mM) applications, subsequent to the application of compounds **2**, **6**, **28**, and **33** to α9α10-nAChRs or compounds **2**, **28**, and **33** to α7-nAChRs. As illustrated in Figure 3B, **6** (which has no intrinsic efficacy at α7-nAChR) did, however, significantly block subsequent ACh-induced function at α9α10-nAChRs.

Moving to α7-nAChR responses, compound **33** (which has the highest α7-nAChR affinity and antagonist potency) was the only one in the series to produce an α7-nAChR rebound current (Figure 3A). This α7-nAChR rebound current induced by **33** was small in terms of peak amplitude, increased slowly, and was very slow to return to baseline. As a result of these slow response kinetics, the intrinsic activity was significantly higher when assessed as AUC than in terms of peak current (57 vs 7%, respectively). Notably, no distinct peak of function was induced by a subsequent control application of ACh; the block of subsequent ACh-induced α7-nAChR activity by compound **33** was thus essentially complete (Figure 3B).

**Figure 4:**
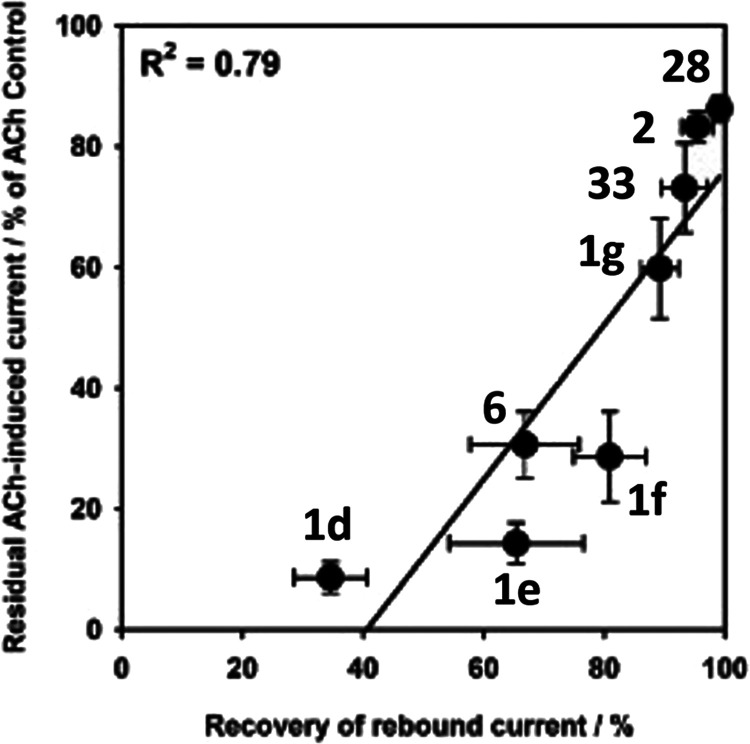
Relationship between residual ACh function after the application of test compounds to α9α10-nAChR and the extent to which the rebound currents that they induce are able to recover before the final ACh application is made (see the text for how these values were calculated). On the *Y*-axis, the mean amplitude ± S.E.M. of the residual ACh-induced current as % of the previously established ACh control amplitude; on the *X*-axis, the mean ± S.E.M. recovery of the rebound current as % of the peak rebound current.

We wished to examine if there was a correlation between the recovery of the rebound currents induced by test compounds at α9α10-nAChR and suppression of the final ACh control stimulation that follows the test compound application. These were calculated, respectively, as “recovery of rebound current” (the percentage by which the rebound current had returned to the prior baseline 1 min following application of the test compound; normalized for each individual oocyte to the peak amplitude of the rebound current over baseline) and “residual ACh-induced current” (i.e., the final ACh control stimulation applied 1 min following the application of the test compound, normalized for each oocyte as a percentage of the amplitude of the mean of the ACh control applications applied before the test compound was applied). Please refer to the Supporting Information Data (page 20) for an illustration of these terms. In our prior publication, we speculated that slow and incomplete recovery of the rebound current preceding the application of the final ACh control pulse could substantially suppress the functional response to the subsequent and final ACh control application.^7^ In Figure 4, we plot “residual ACh-induced current” (*y*-axis) against “recovery of rebound current” (*x*-axis) for the previously published compounds **1d**, **1e**, **1f**, **1g**, and **2** along with the new compounds **6**, **28**, and **33**. Please note that, in this figure, only current amplitude data could be used since our previous publication assessed only peak response, and not AUC, values. As can be seen, there is a strong correlation between incomplete recovery of rebound current when the final ACh control pulse is delivered and increased inhibition of ACh-induced currents at α9α10-nAChR. Compounds **2**, **28**, and **33** showed an almost complete recovery and the highest residual ACh-induced currents, whereas compounds **1d**, **1e**, and **1f** showed a largely incomplete recovery and the lowest residual ACh-induced currents. This confirms what we had speculated in our prior publication,^7^ reinforcing the suggestion that the longer the duration of the rebound current (i.e., the slower the disassociation of the compound of interest from the α9α10-nAChR), the greater the suppression of subsequent ACh-induced activity is.

## Discussion

**Chart 3:**
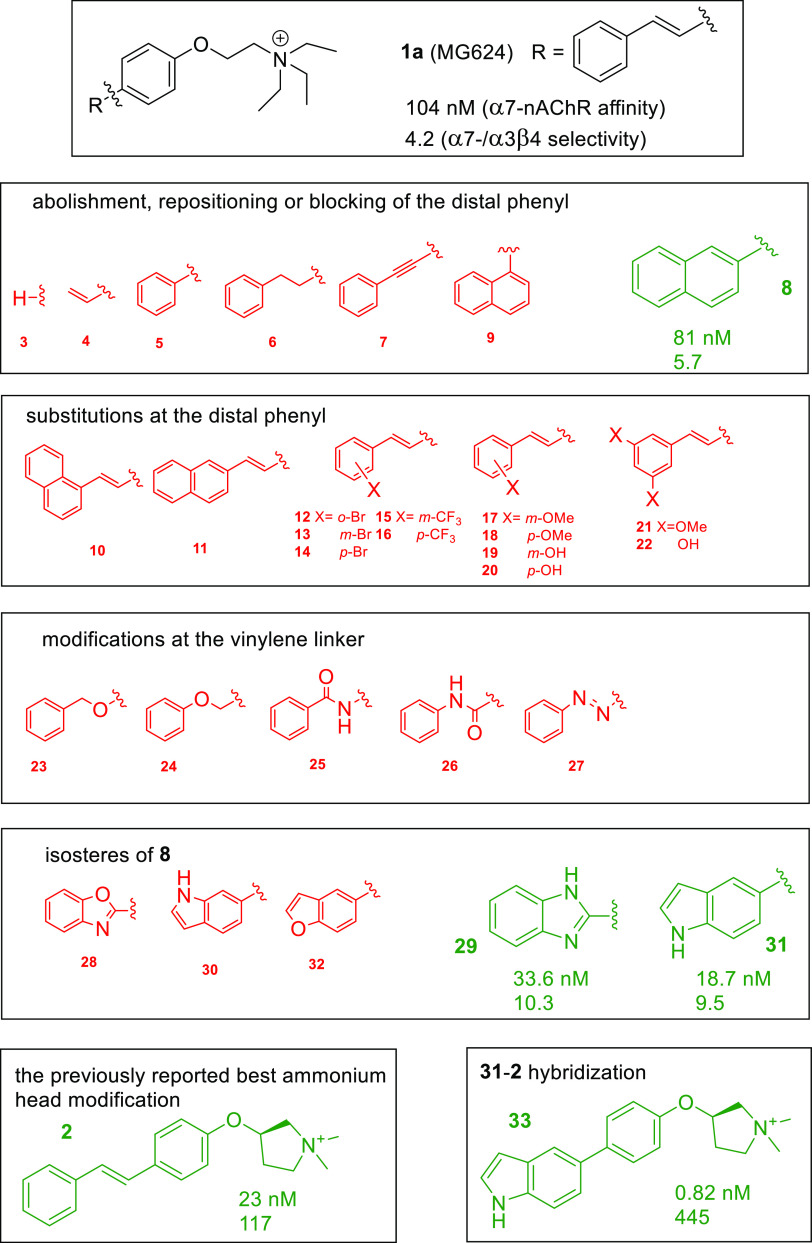
**1a** Analogues with Productive (in Green) and Unproductive (in Red) Modifications in Terms of α7-nAChR Affinity and α7- vs α3β4-nAChR Selectivity (Both Reported for the Ref (1)a and for the Improving Modifications)

We began by determining the α7-nAChR binding affinity and selectivity over α3β4-nAChR. Regardless of their electronic effects, all of the accomplished modifications of **1a** (*K*_i_ value at α7-nAChR = 104 nM) imply an increase of the steric bulk of the distal phenyl group (compounds **10**–**22**) resulting in significantly lower α7-nAChR affinities. These bulk-increasing modifications also lowered, and sometimes reversed, α7- vs α3β4-nAChR selectivity. Such outcomes indicate that the extension of the styryl residue of **1a** is a critical issue. Otherwise, within the set of compounds **3**–**9** (which abolished the distal phenyl or simply varied its positioning), the 2-naphthyl analogue **8** showed a profile of α7-nAChR affinities and α7- over α3β4-nAChR selectivities very similar to that of **1a**. This suggests that the coplanarity of vinylene and phenyl is a requisite of the active conformer of **1a**. Among the compounds modified at the vinylene linker (compounds **23**–**27**), moderate α7-nAChR affinities are shown only by **26** and **27**. Notably, these two compounds, having an amide and diazo linker, respectively, maintain the original styryl rigidity (unlike **23** and **24**, which have a flexible methyleneoxy linker). Further, unlike the other benzamide **25**, compounds **26** and **27** are superimposable to **1a** and to its intramolecular cyclized 2-naphthyl analogue **8**, respectively. These SARs are supported by the subsequent five compounds (**28**–**32**), which are all isosteres of **8**, thus rigidified analogues of **1a**, in which the 2-naphthyl of **8** is replaced by a heteroaromatic bicycle without extension with respect to the original styryl moiety but with additional interaction potential due to the presence of heteroatoms. Three of them (**28**, **30**, and **32**) show moderate α7-nAChR affinity and the other two, **29** and **31**, show high α7-nAChR affinity (33.6 and 18.7 nM *K*_i_, respectively). Compared to **1a** and **8**, benzimidazole **29** and indole **31** have not only significantly higher α7-nAChR affinity but also increased α7- vs α3β4-nAChR selectivity. In both, a critical role is played by NH, as indicated by the loss of α7-nAChR affinity resulting from its replacement with O (cf. **29** with **28** and **31** with **32**) or its repositioning (cf. **31** with **30**). Consistently with all of these observations, a great step forward is achieved by combining the two best modifications of **1a**, in terms of α7-nAChR affinity and selectivity, at the stilbene scaffold and at the 2-ammonium ethyl portion, respectively: the replacement of the stilbene scaffold with 4-(5-indolyl)phenyl (compound **31**, 18.7 nM *K*_i_) and the previously reported constraint of the 2-ammoniumethyloxy portion into (*R*)-3-pyrrolidiniumoxy substructure (compound **2**, 23 nM *K*_i_). The effects of these two modifications are synergic and the resulting hybrid **33** displays subnanomolar α7-nAChR affinity and very high α7- vs α3β4- and α4β2-nAChR selectivities. In Chart 3, all of the above structure–affinity relationships are summarized and visualized reproducing, for clarity, the same subdivision of the styryl modifications as in Chart 2 and representing the productive and the unproductive ones compared to **1a** in green and red, respectively.

**Figure 5:**
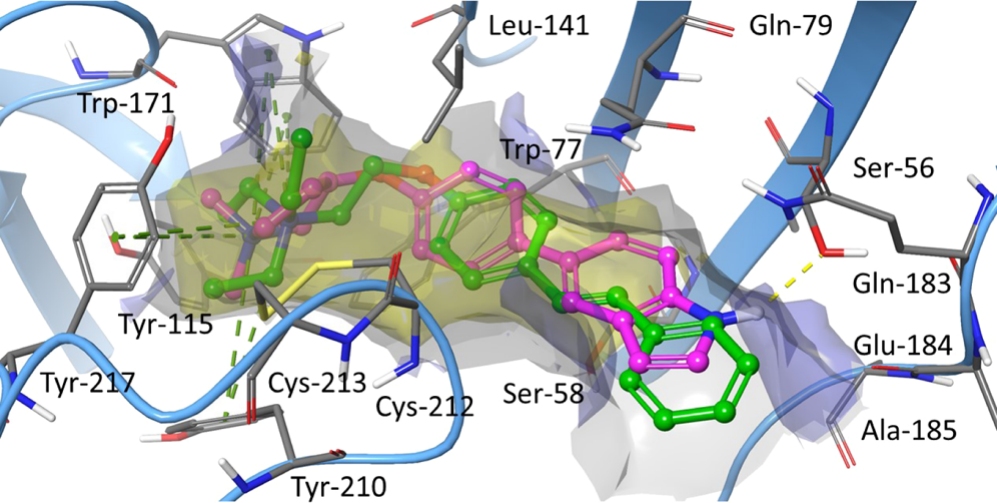
Proposed binding mode of **33** (pink) and **1a** (green) at the α7-nAChR orthosteric binding site (PDB ID: 7EKP). The receptor backbone is represented by sky-blue cartoons, and individual residues defining the binding site are colored in gray. π–cation interactions are shown as dashed green lines, while hydrogen bonds are shown as yellow dashed lines. The inner surface of the binding pocket is depicted in gray, lipophilic areas are in yellow, and hydrophilic areas where the H-bond donor is favored are in blue. Superimposition of the docking poses of **33** and **1a** reveals the critical H-bonding between the indole moiety of **33** and the distal hydrophilic area of the α7α7 binding site, plausibly responsible for 120 times higher affinity.

To further elucidate the structural determinants for so high an increase in affinity at the α7-nAChR subtype, the molecular docking of **1a** and **33** at the orthosteric binding pocket of the α7α7 dimer extracted and refined from the recently reported cryo-EM structure 7EKP was performed.^13^ As shown in Figure 5, both **33** and **1a** assume a similar binding pose at the α7α7 subunit interface, superimposable with EVP-6124, the ligand complexed with the receptor in the original cryo-EM (not shown). In detail, the permanently charged quaternary ammonium head of both **33** and **1a** is accommodated within the aromatic box formed by Tyr-115, Trp-171, Tyr-217, and Tyr-210, with which they establish π–cation interactions. Comparison between the shorter and more rigid N–O linker of **33** and the longer and flexible linker of **1a** highlights an important difference in how far the aromatic moiety protrudes into the binding pocket: whereas the indole ring of **33** is still embedded within it, the distal aromatic ring of **1a** extends out. As illustrated from the binding site analysis, both the *O*-linked styryl portion of **1a** and the biphenyl portion of **33** are sandwiched in a lipophilic and narrow area (in yellow) between Leu-141 and Gln-79 at the top and Trp-77 and Ser-58 at the bottom. Instead, the terminal pyrrole group of (*R*)-**33** is positioned at the hydrophilic entrance of the binding pocket, where H-bond donors are strongly preferred due to the presence of multiple H-bond acceptors on the target (such as the side chain of Ser-56 or the carbonyl of Glu-184). The additional H-bond network, together with a better fit in the binding pocket, is compatible with the 120 times increase of affinity from **1a** to **33**.

Also here, as for the previously reported analogues of **1a** modified at the ammonium ethyl residue,^7^*in vitro* functional activity at the α7 and α9α10-nAChRs was determined for a selection of analogues, 9 among the 31 initially tested for binding affinities. As explained above, the selection was centered on benzimidazole **29** and indoles **31** and **33**, having the best α7-nAChR profiles; some of their strictest analogues (**25**, **26**, **28**, and **30**) and, for the representativeness of the vinylene modification, compounds **6** and **27** were then recruited. According to such criteria, as in the previously reported selection of 12 **1a** analogues modified at the ammonium ethyl residue,^7^ compounds with modest or moderate α7-nAChR affinity (see **6**, **25**, **26**, and **27**) were tested for *in vitro* functional activity as well as compounds with good or high α7-nAChR affinity (**28**, **29**, **30**, **31**, and **33**). It is therefore significant that we obtained, applying selection criteria including a wide range of α7-nAChR affinities in both cases, divergent results for the two series of compounds (those made here vs in the preceding study^7^). Indeed, among the previously published **1a** analogues (those modified at the ethyl ammonium head), we found only compounds unable to produce 100% inhibition of the ACh-induced function at the α7-nAChR or even completely devoid of α7-nAChR antagonism, but all antagonizing ACh activity at α9α10-nAChR. In contrast, among the present **1a** analogues modified at the stilbene scaffold in the current study, only one compound, the 4-(2-phenylethyl)phenyl analogue **6**, was devoid of α7-nAChR antagonism; all of the other compounds inhibited ACh-induced function at both α7- and α9α10-nAChR subtypes and, in the case of the three indolyl analogues **30**, **31**, and **33**, produced 100% inhibition. Notably, compound **6** is, among the selected nine compounds, the one with the poorest α7-nAChR affinity, which could be imputed to the loss of that beneficial coplanarity suggested by the comparison of **8** with **1a**. Overall, the modifications at the ethyl ammonium portion of **1a** seem effective in impairing the interaction with the α7-nAChR, while α7- vs α9α10-nAChR selective antagonism can be achieved only by modifying both the stilbene scaffold and the ethyl ammonium head, as demonstrated by hybrid **33**, endowed with subnanomolar α7-nAChR affinity, 100% inhibition of ACh-induced function at the α7-nAChR, and good antagonist selectivity for α7- over α9α10-nAChR.

## Conclusions

If one considers the results obtained with the present modifications and those previously reported of **1a**, one can immediately see that we have found in our prior publication several **1a** analogues producing antagonism through a mechanism that we speculated was compatible with the open-channel block at α9α10-nAChR while being completely devoid of α7-nAChR antagonism. In contrast, the present study identified no **1a** analogue with the opposite profile (i.e., block of α7-nAChR without α9α10-nAChR antagonism). Against this trend, all of the compounds that behave as antagonists at both the receptor subtypes are more potent at α7- than at α9α10-nAChR, except **26** in the present series. However, a marked α7- vs α9α10-nAChR-selective antagonism remains elusive. Only **33** shows a significantly selective antagonism at the α7-nAChR together with 100% inhibition of ACh-induced function at both the receptor subtypes. As depicted in Figure 3, it is the only one of the tested compounds that produces a profound loss of subsequent ACh-induced function at the α7-nAChR subtype (Figure 3B) and the only one that also produces a measurable rebound current at this same subtype (Figure 3A). These features of **33** at α7-nAChR are similar to those that we have described for multiple structurally related (but α9α10-nAChR-selective) antagonists and previously noted to be compatible with an open-channel blocker mechanism. However, as noted in the Introduction section, further experimentation is required to draw a firm conclusion as to the precise mechanism by which these compounds exert antagonism.

Overall, these results show that making the α7- and α9α10-nAChR antagonist **1a** ineffective on one of the two subtypes or highly subtype-selective is not equally simple in both directions. Single modifications, such as the increase of the ammonium head bulkiness or rigidification of the ethylene linker (**1d**–**1g**),^7^ but also simple saturation of the vinylene bridge (**6**) are sufficient to profoundly or completely impair the effects at the α7-nAChR while maintaining the inhibition of ACh function at the α9α10-nAChR. On the other hand, we have not found single modifications of **1a** resulting in the exact opposite behavior. However, we were able to obtain a complete loss of residual ACh-induced function at the α7-nAChR while leaving almost unaltered the residual ACh-induced function at the other subtype by making modifications at both the portions of **1a**, the stilbene and the ethyl ammonium head. These modifications leading to **33** were suggested by the high α7-nAChR affinities of compounds **2** and **31**, modified at the ethyl ammonium head and at the stilbene, respectively.

We can thus note that, with regard to **1a** modifications, the α9α10-nAChR shows a wider tolerance for structural modifications than the α7-nAChR and this may account for the fact that differentiating α9α10-nAChR antagonism from α7-nAChR antagonism, using **1a** as a starting hit, is less difficult than the reverse outcome, for which a finer modulation of the molecular features of the hit is required.

There is a great interest in the physiological roles of α7- and α9α10-nAChR and their druggability for the development of optimized therapeutics.^14^ To this end, the production of ligands that can reliably discriminate functional effects mediated by α7- or α9α10-nAChR is absolutely critical. The identification of **33** and **1d** as selective antagonists, at one or the other receptor subtype, having strictly related structures and the same potential mechanism of action, provides a valuable pair of tools and a great aid to future work that will rationally generate new, even-more selective agents.

## Experimental Section

### Chemistry

All chemicals and solvents were used as received from commercial sources or prepared, as described in the literature. Flash chromatography purifications were performed using KP-Sil 32–63 μm 60 Å cartridges. Thin-layer chromatography (TLC) analyses were carried out on alumina sheets precoated with silica gel 60 F254 and visualized with UV light. The content of saturated aqueous solution of ammonia in eluent mixtures is given as v/v percentage. *R*_*f*_ values are given for guidance. ^1^H NMR spectra were recorded at 600, 400, 300, or 200 MHz, while ^13^C NMR spectra were recorded at 150, 100, or 75 MHz using FT-NMR spectrometers. Chemical shifts are reported in ppm relative to residual solvent (CHCl_3_, MeOH, or DMSO) as the internal standard. Melting points were determined by a Buchi Melting Point B-540 apparatus. Optical rotations were determined using a Jasco P-1010 polarimeter. Liquid chromatography–mass spectrometry (LC–MS) analysis was performed using an Agilent 1200 series solvent delivery system equipped with an autoinjector coupled to a PDA and an Agilent 6400 series triple quadrupole electrospray ionization detector. Gradients of 5% aqueous MeCN + 0.1% HCO_2_H (solvent A), and 95% aqueous MeCN + 0.05% HCO_2_H (solvent B) were employed. Purity was measured by analytical high-performance liquid chromatography (HPLC) on an UltiMate HPLC system (Thermo Scientific) consisting of an LPG-3400A pump (1 mL/min), a WPS-3000SL autosampler, and a DAD-3000D diode array detector using a Gemini-NX C18 column (4.6 mm × 250 mm, 3 μm, 110 Å); gradient elution 0–100% B (MeCN/H_2_O/TFA, 90:10:0.1) in solvent A (H_2_O/TFA, 100:0.1) over 20 min. Data were analyzed using Chromeleon Software v. 6.80. Purity is ≥ 95%, and retention times (*R*_t_) are reported.

### Method A

Under a nitrogen atmosphere, a suspension of the appropriate phenol (10 mmol, 1 equiv), K_2_CO_3_ (2.0–4 equiv), and KI (0.1 equiv) in the specified solvent (15 mL) was vigorously stirred at reflux temperature for 30 min. The appropriate alkylating agent (1.2–4.2 equiv) was added portionwise or dropwise, and the resulting mixture was refluxed overnight unless specified otherwise. The reaction mixture was cooled to room temperature, and the solid was removed by filtration. The filtrate was concentrated under vacuum, and the crude was purified as specified. The desired products **34**, **35**, **39**, **43**, **52**–**57**, **60**, **61**, **66**, **69**, **71**, **73**, **74**, **76**, **79**, **81**, **93**, **95**, **98**, **102**, and **103** were obtained as oils or solids in variable yields (23–100%).

### Method B

The appropriate tertiary amine (2.59 mmol, 1 equiv) was dissolved in the specified solvent (5 mL), and iodoethane (1.2–50 equiv) was added dropwise. The reaction mixture was vigorously stirred at the specified temperature for 1–24 h. The reaction was worked up and purified as specified. The desired compounds **3–6**, **8**–**14**, **16–18**, **23**–**32**, and **67** were obtained as solids in variable yields (20–100%).

### Method C

All of the solvents used were previously degassed. Under an inert atmosphere, the specified aryl bromide (1.3 mmol, 1 equiv) was dissolved in either 1,2-dimethoxyethane or a mixture toluene/EtOH 1:1 (5 mL). Upon the addition of a solution of Pd(PPh_3_)_4_ (0.35 equiv) in the same solvent (2 mL), the reaction mixture was stirred for 20 min. Afterward, a mixture of EtOH (2 mL)/2 M_aq_ Na_2_CO_3_ (4 mL) was added dropwise. When specified, TBAB (0.05 equiv) was also added. A solution of the appropriate boronic acid (1.1 equiv) in 1,2-dimethoxyethane (5 mL) was added dropwise, and the reaction mixture was refluxed overnight. Upon evaporation of the solvent under reduced pressure, the residue was diluted in DCM and filtered through a silica pad, and the solvent was evaporated under reduced pressure. The crude was purified as specified, providing compounds **78**, **80**, **94**, **96**, and **101** as oils or solids in moderate to high yields (42–92%).

### Method D

The appropriate alkyl halide (2.20 mmol, 1 equiv) was dissolved in a saturated solution of NaI in acetone (10 mL), and the reaction mixture was stirred at reflux temperature overnight. A 10% aqueous solution of Na2S2O5 (20 mL) was added, and the mixture was stirred for 1 h at room temperature. After evaporation of acetone under reduced pressure, the resulting aqueous suspension was extracted with diethyl ether twice. The organic layers were combined and washed with water and then brine. The organic phase was dried over anhydrous Na_2_SO_4_, filtered, and evaporated under reduced pressure. The desired products **36**, **44**, **62**, **63**, **75**, **77, 86**, and **90** were obtained as oils or solids in high yields (72–97%).

### Method E

Unless specified otherwise, a solution of the appropriate alkyl iodide (1.35 mmol, 1 equiv) and diethylamine (50 equiv) in toluene (10 mL) was heated at 60 °C for 3–4 h. Upon cooling to room temperature, the mixture was washed with water three times. The organic phase was dried over anhydrous Na_2_SO_4_, filtered, and the solvent was evaporated under reduced pressure. The crude was purified as specified, providing the desired compounds **37**, **45**, **87**, and **91** as oils or solids in high yields (84–100%).

### Method F

Under an inert atmosphere, a mixture of the appropriate aryl iodide (2.14 mmol, 1 equiv), Pd(OAc)_2_ (0.1 equiv), and anhydrous triethylamine (2.1 equiv) in CH_3_CN (5 mL) was stirred at room temperature for 30 min and then 4-vinylphenol **42** (1.1–1.5 equiv) was added. The resulting mixture was stirred at reflux temperature overnight. Upon cooling to room temperature, the reaction mixture was concentrated under reduced pressure. The residue was diluted with cold 10% aqueous HCl solution (10 mL) and extracted with EtOAc three times. The combined organic phases were dried over anhydrous Na_2_SO_4_, filtered, and the solvent was evaporated under reduced pressure. The crude was purified as specified, providing the desired compounds **46**–**51**, **58**, **59**, and **65** as oils or solids in low to modest yields (21–66%).

#### Synthesis of *N*,*N*,*N*-Triethyl-2-phenoxyethan-1-aminium Iodide (**3**)

Obtained from *N*,*N*-diethyl-2-phenoxyethan-1-amine **34** (500 mg, 2.59 mmol, 1 equiv) and iodoethane (8 equiv) in 1,2-dichloroethane (5 mL), according to Method B, overnight at room temperature. Upon rotary evaporation of the volatiles, the residue was dissolved in MeOH and diluted with diethyl ether. The suspension was filtered, and the solid was washed with diethyl ether. Trituration with diisopropyl ether/2-propanol provided the desired product **3** as a dark solid in a 48% yield. Mp = 79–83 °C. *R*_t_ (LC-MS) = 2.867 min. LC-MS (ESI): *m*/*z* calcd for C_14_H_24_NO [M]^+^ = 222.19, found 222.2. *R*_t_ (HPLC) = 9.28 min. ^1^H NMR (300 MHz, chloroform-*d*) δ 7.27 (t, *J* = 7.7 Hz, 2H), 7.03–6.86 (m, 3H), 4.50–4.40 (m, 2H), 4.01–3.87 (m, 2H), 3.55 (q, *J* = 7.2 Hz, 6H), 1.42 (t, *J* = 7.2 Hz, 9H). ^13^C NMR (75 MHz, chloroform-*d*) δ 157.0, 129.9, 122.2, 114.6, 62.0, 57.0, 54.8, 8.6.

#### Synthesis of *N*,*N*,*N*-Triethyl-2-(4-vinylphenoxy)ethan-1-aminium Iodide (**4**)

Obtained from *N*,*N*-diethyl-2-(4-vinylphenoxy)ethan-1-amine **45** (150 mg, 0.68 mmol, 1 equiv) and iodoethane (1.2 equiv) in DCM (2 mL) according to Method B, at reflux temperature for 2 h. Upon cooling, the suspension was filtered and the solid was recrystallized from diethyl ether, providing the desired product as a pale-yellow solid in a 43% yield. Mp = 100.1 °C. *R*_t_ (LC-MS) = 3.158 min. LC-MS (ESI): *m*/*z* calcd for C_24_H_30_NO [M]^+^ = 248.20, found 248.2. *R*_t_ (HPLC) = 11.01 min. ^1^H NMR (300 MHz, chloroform-*d*) δ 7.33 (d, *J* = 8.7 Hz, 2H), 6.90 (d, *J* = 8.7 Hz, 2H), 6.62 (dd, *J* = 17.6, 10.9 Hz, 1H), 5.60 (d, *J* = 17.6 Hz, 1H), 5.14 (d, *J* = 10.9 Hz, 1H), 4.49 (t, *J* = 4.6 Hz, 2H), 4.03–3.92 (m, 2H), 3.57 (q, *J* = 7.2 Hz, 6H), 1.44 (t, *J* = 7.2 Hz, 9H). ^13^C NMR (75 MHz, chloroform-*d*) δ 156.8, 135.9, 132.0, 127.8, 114.8, 112.7, 62.2, 57.1, 54.9, 8.7.

#### Synthesis of 2-([1,1′-Biphenyl]-4-yloxy)-*N*,*N*,*N*-triethylethan-1-aminium Iodide (**5**)

Obtained from 2-([1,1′-biphenyl]-4-yloxy)-*N*,*N*-diethylethan-1-amine **37** (680 mg, 2.52 mmol, 1 equiv) and iodoethane (4 equiv) in DCM (7 mL) according to Method B, at reflux temperature for 2 h. Upon cooling, the suspension was filtered and the solid was recrystallized from diethyl ether, providing the desired compound **5** as an off-white solid in an 80% yield. Mp = 167.4 °C. *R*_t_ (LC-MS) = 3.608 min. LC-MS (ESI): *m*/*z* calcd for C_20_H_28_NO [M]^+^ = 298.22, found 298.3. *R*_t_ (HPLC) = 12.63 min. ^1^H NMR (300 MHz, chloroform-*d*) δ 7.56–7.48 (m, 4H), 7.44–7.36 (m, 2H), 7.34–7.27 (m, 1H), 7.07–6.97 (m, 2H), 4.55 (t, *J* = 4.5 Hz, 2H), 4.09–4.01 (m, 2H), 3.60 (q, *J* = 7.2 Hz, 6H), 1.47 (t, *J* = 7.2 Hz, 9H). ^13^C NMR (75 MHz, chloroform-*d*) δ 156.6, 140.3, 135.4, 128.9, 128.6, 127.2, 126.9, 115.1, 62.4, 57.2, 55.0, 8.7.

#### Synthesis of *N*,*N*,*N*-Triethyl-2-(4-phenethylphenoxy)ethan-1-aminium Iodide (**6**)

Obtained from **73** (150 mg, 0.504 mmol) and iodoethane (4 equiv) in toluene (6 mL) at 90 °C, according to Method B. After 48 h, diethyl ether was added to the mixture and the solid was collected by filtration to give **6** as an off-white solid (96 mg, 42%). Mp = 122–123 °C. *R*_t_ (LC-MS) = 3.756 min. LC-MS (ESI): *m*/*z* calcd for C_22_H_32_NO^+^ [M]^+^ = 326.25, found 326.3. *R*_t_ (HPLC) = 13.56 min. ^1^H NMR (300 MHz, methanol-*d*_4_) δ 7.26–7.18 (m, 2H), 7.17–7.07 (m, 5H), 6.89 (d, *J* = 8.6 Hz, 2H), 4.44–4.35 (m, 2H), 3.78–3.70 (m, 2H), 3.48 (q, *J* = 7.2 Hz, 6H), 2.86 (s, 4H), 1.37 (t, *J* = 7.2 Hz, 9H). ^13^C NMR (75 MHz, methanol-*d*_4_) δ 157.1, 142.9, 136.5, 130.8, 129.6, 129.2, 126.8, 115.4, 62.6, 57.2, 54.9, 39.2, 38.1, 7.9.

#### Synthesis of *N*,*N*,*N*-Triethyl-2-(4-(phenylethynyl)phenoxy)ethan-1-aminium Iodide (**7**)

A solution of 1-(2-iodoethoxy)-4-(phenylethynyl)benzene **75** (125 mg, 0.36 mmol, 1 equiv) in toluene (3 mL) and triethylamine (3 mL) was stirred at room temperature overnight. The resulting suspension was filtered, and the solid was washed with EtOAc, providing the desired product **7** as an off-white solid in a 37% yield. Mp = 162.2 °C dec. *R*_t_ (LC-MS) = 3.787 min. LC-MS (ESI): *m*/*z* calcd for C_22_H_28_NO^+^ [M]^+^ = 322.22, found 322.2. *R*_t_ (HPLC) = 13.69 min. ^1^H NMR (600 MHz, chloroform-*d*) δ 7.52–7.46 (m, 4H), 7.36–7.30 (m, 3H), 6.94 (d, *J* = 8.8 Hz, 2H), 4.56 (t, *J* = 4.7 Hz, 2H), 4.12–4.05 (m, 2H), 3.60 (q, *J* = 7.2 Hz, 6H), 1.51–1.44 (m, 9H). ^13^C NMR (150 MHz, chloroform-*d*) δ 156.9, 133.5, 131.6, 128.5, 128.3, 123.4, 117.4, 114.8, 89.0, 88.9, 62.4, 57.2, 55.0, 8.7.

#### Synthesis of *N*,*N*,*N*-Triethyl-2-(4-(naphthalen-2-yl)phenoxy)ethan-1-aminium Iodide (**8**)

Obtained from *N*,*N*-diethyl-2-(4-(naphthalen-2-yl)phenoxy)ethan-1-amine **79** (33 mg, 0.1 mmol, 1 equiv) according to Method B, using ethyl iodide as a solvent (2 mL) for 30 min at reflux temperature. Upon cooling, the reaction mixture was diluted with diethyl ether and the resulting suspension was filtered. The solid was washed with DCM, providing the desired product **8** as a white solid in a 76% yield. Mp = 242 °C. *R*_t_ (LC-MS) = 3.864 min. LC-MS (ESI): *m*/*z* calcd for C_24_H_30_NO [M]^+^ = 348.23, found 348.3. *R*_t_ (HPLC) = 14.11 min. ^1^H NMR (300 MHz, methanol-*d*_4_) δ 8.04 (d, *J* = 1.9 Hz, 1H), 7.96–7.83 (m, 3H), 7.79–7.71 (m, 3H), 7.55–7.40 (m, 2H), 7.14 (d, *J* = 8.8 Hz, 2H), 4.56–4.47 (m, 2H), 3.84–3.76 (m, 2H), 3.51 (q, *J* = 7.2 Hz, 6H), 1.40 (t, *J* = 7.2 Hz, 9H). ^13^C NMR (75 MHz, methanol-*d*_4_) δ 158.6, 139.0, 136.1, 134.0, 129.6, 129.5, 129.1, 128.6, 127.4, 126.9, 126.1, 126.0, 124.4, 116.1, 62.7, 57.2, 55.0, 7.9.

#### Synthesis of *N*,*N*,*N*-Triethyl-2-(4-(naphthalen-1-yl)phenoxy)ethan-1-aminium Iodide (**9**)

Obtained from *N*,*N*-diethyl-2-(4-(naphthalen-1-yl)phenoxy)ethan-1-amine **81** (70 mg, 0.22 mmol, 1 equiv) according to Method B, using iodoethane as a solvent (2 mL), at reflux temperature, for 30 min. Upon cooling, the reaction mixture was diluted with diethyl ether and the resulting suspension was filtered. The solid was washed with DCM, providing the desired product **9** as a pale-yellow solid in a 54% yield. Mp = 178 °C. *R*_t_ (LC-MS) = 3.792 min. LC-MS (ESI): *m*/*z* calcd for C_24_H_30_NO [M]^+^ = 348.23, found 348.3. *R*_t_ (HPLC) = 13.97 min. ^1^H NMR (300 MHz, methanol-*d*_4_) δ 7.96–7.78 (m, 3H), 7.57–7.35 (m, 6H), 7.16 (d, *J* = 8.9 Hz, 2H), 4.59–4.49 (m, 2H), 3.87–3.77 (m, 2H), 3.53 (q, *J* = 7.2 Hz, 6H), 1.41 (t, *J* = 7.2 Hz, 9H). ^13^C NMR (75 MHz, methanol-*d*_4_) δ 158.3, 140.9, 135.7, 135.4, 133.0, 132.4, 129.4, 128.6, 127.9, 127.0, 126.8, 126.7, 126.4, 115.6, 62.8, 57.2, 55.0, 8.0.

#### Synthesis of (*E*)-*N*,*N*,*N*-Triethyl-2-(4-(2-(naphthalen-1-yl)vinyl)phenoxy)ethan-1-aminium Iodide (**10**)

Obtained from compound **69** (1.00 g, 2.89 mmol) and iodoethane (10 equiv) in EtOH according to Method B at 70 °C for 18 h. The mixture was concentrated under vacuum and purified by flash chromatography (DCM/MeOH 95:5) to give **10** as a pale-yellow solid in a 48% yield. Mp = 164.5–166.5 °C (crystallized from EtOH/MeOH 8:2). *R*_t_ (LC-MS) = 3.951 min. LC-MS (ESI): *m*/*z* calcd for C_26_H_32_NO [M]^+^ = 374.25, found 374.2. *R*_t_ (HPLC) = 14.66 min. ^1^H NMR (300 MHz, dimethylsulfoxide-*d*_6_) δ 8.45–8.36 (m, 1H), 8.01–7.91 (m, 2H), 7.86 (dd, *J* = 7.7, 2.6 Hz, 2H), 7.76 (d, *J* = 8.7 Hz, 2H), 7.69–7.48 (m, 3H), 7.26 (d, *J* = 16.1 Hz, 1H), 7.05 (d, *J* = 8.7 Hz, 2H), 4.45 (t, *J* = 4.7 Hz, 2H), 3.70 (t, *J* = 4.7 Hz, 2H), 3.40 (q, *J* = 7.0 Hz, 6H), 1.25 (t, *J* = 7.0 Hz, 9H). ^13^C NMR (100 MHz, dimethylsulfoxide-*d*_6_) δ 157.1, 134.4, 133.4, 130.8, 130.7, 130.7, 128.4, 128.2, 127.5, 126.1, 125.9, 125.8, 123.8, 123.0, 122.8, 114.8, 61.1, 55.2 52.9, 7.3.

#### Synthesis of (*E*)-*N*,*N*,*N*-Triethyl-2-(4-(2-(naphthalen-2-yl)vinyl)phenoxy)ethan-1-aminium Iodide (**11**)

Obtained from compound **71** (1.00 g, 2.89 mmol) and iodoethane (10 equiv) in EtOH according to Method B at 70 °C for 14 h. The mixture was concentrated under vacuum and purified by flash chromatography (DCM/MeOH 95:5). The product was crystallized from MeOH affording **11** as a white solid in a 24% yield. Mp = 223–227 °C. *R*_t_ (LC-MS) = 4.080 min. LC-MS (ESI): *m*/*z* calcd for C_26_H_32_NO [M]^+^ = 374.25, found 374.2. *R*_t_ (HPLC) = 14.77 min. ^1^H NMR (300 MHz, dimethylsulfoxide-*d*_6_) δ 8.01–7.95 (m, 1H), 7.94–7.81 (m, 4H), 7.70–7.60 (m, 2H), 7.55–7.43 (m, 2H), 7.39 (d, *J* = 16.5 Hz, 1H), 7.30 (d, *J* = 16.5 Hz, 1H), 7.10–6.99 (m, 2H), 4.44 (t, *J* = 4.8 Hz, 2H), 3.70 (t, *J* = 4.8 Hz, 2H), 3.40 (q, *J* = 7.1 Hz, 6H), 1.25 (t, *J* = 7.1 Hz, 9H). ^13^C NMR (100 MHz, dimethylsulfoxide-*d*_6_) δ 157.1, 134.9, 133.3, 132.4, 130.6, 128.4, 128.1, 127.8, 127.7, 127.5, 126.6, 126.4, 125.9, 125.8, 123.5, 114.9, 61.1, 55.2, 52.9, 7.3.

#### Synthesis of (*E*)-2-(4-(2-Bromostyryl)phenoxy)-*N*,*N*,*N*-triethylethan-1-aminium Iodide (**12**)

Obtained from (*E*)-2-(4-(2-bromostyryl)phenoxy)-*N*,*N*-diethylethan-1-amine **52** (21 mg, 0.56 mmol, 1 equiv) and iodoethane (10 equiv) in THF (5 mL), according to Method B, at reflux temperature, overnight. Upon cooling at room temperature, the reaction mixture was diluted with diethyl ether and the resulting suspension was filtered, providing the desired product **12** as a pale-yellow solid in a 100% yield. Mp = 133.3 °C. *R*_t_ (LC-MS) = 3.926 min. LC-MS (ESI): *m*/*z* calcd for C_22_H_29_BrNO [M]^+^ = 402.14, 404.14, found 402.1, 404.1. *R*_t_ (HPLC) = 14.53 min. ^1^H NMR (300 MHz, chloroform-*d*) δ 7.62 (dd, *J* = 7.8, 1.6 Hz, 1H), 7.55 (dd, *J* = 8.1, 1.2 Hz, 1H), 7.47 (d, *J* = 8.7 Hz, 2H), 7.35–7.25 (m, 2H), 7.14–7.03 (m, 1H), 6.99–6.91 (m, 3H), 4.57–4.49 (m, 2H), 4.05–3.96 (m, 2H), 3.57 (q, *J* = 7.2 Hz, 6H), 1.45 (t, *J* = 7.2 Hz, 9H). ^13^C NMR (75 MHz, chloroform-*d*) δ 157.0, 137.1, 133.1, 131.3, 130.5, 128.8, 128.5, 127.7, 126.7, 126.2, 124.1, 115.0, 62.3, 57.1, 54.9, 8.7.

#### Synthesis of (*E*)-2-(4-(3-Bromostyryl)phenoxy)-*N*,*N*,*N*-triethylethan-1-aminium Iodide (**13**)

Obtained from (*E*)-2-(4-(3-bromostyryl)phenoxy)-*N*,*N*-diethylethan-1-amine **53** (290 mg, 0.78 mmol, 1 equiv) and iodoethane (10 equiv) in THF (5 mL), according to Method B at reflux temperature, overnight. Upon cooling at room temperature, the reaction mixture was diluted with diethyl ether and the resulting suspension was filtered, providing the desired product **13** as a white solid in a 100% yield. Mp = 133.3 °C. *R*_t_ (LC-MS) = 3.985 min. LC-MS (ESI): *m*/*z* calcd for C_22_H_29_BrNO [M]^+^ = 402.14, 404.14, found 402.1, 404.1. *R*_t_ (HPLC) = 14.60 min. ^1^H NMR (300 MHz, chloroform-*d*) δ 7.59 (t, *J* = 1.8 Hz, 1H), 7.42 (d, *J* = 8.7 Hz, 2H), 7.39–7.30 (m, 2H), 7.18 (t, *J* = 7.8 Hz, 1H), 7.04–6.90 (m, 3H), 6.86 (d, *J* = 16.3 Hz, 1H), 4.57–4.46 (m, 2H), 4.02–3.94 (m, 2H), 3.56 (q, *J* = 7.2 Hz, 6H), 1.44 (t, *J* = 7.2 Hz, 9H). ^13^C NMR (75 MHz, chloroform-*d*) δ 156.9, 139.6, 131.1, 130.3, 129.9, 129.2, 129.1, 128.3, 125.9, 125.1, 122.9, 115.0, 62.3, 57.0, 54.9, 8.6.

#### Synthesis of (*E*)-2-(4-(4-Bromostyryl)phenoxy)-*N*,*N*,*N*-triethylethan-1-aminium Iodide (**14**)

Obtained from (*E*)-2-(4-(4-bromostyryl)phenoxy)-*N*,*N*-diethylethan-1-amine **54** (315 mg, 0.84 mmol, 1 equiv) and iodoethane (2 equiv) in THF (5 mL), according to Method B at reflux temperature, overnight. Upon cooling at room temperature, the reaction mixture was diluted with diethyl ether and the resulting suspension was filtered, providing the desired product **14** as a white solid in a 79% yield. Mp = 244.4 °C. *R*_t_ (LC-MS) = 3.950 min. LC-MS (ESI): *m*/*z* calcd for C_22_H_29_BrNO [M]^+^ = 402.14, 404.14, found 402.1, 404.1. *R*_t_ (HPLC) = 14.68 min. ^1^H NMR (300 MHz, dimethylsulfoxide-*d*_6_) δ 7.60 (d, *J* = 8.8 Hz, 2H), 7.55–7.53 (m, 4H), 7.27 (d, *J* = 16.5 Hz, 1H), 7.12 (d, *J* = 16.5 Hz, 1H), 7.02 (d, *J* = 8.8 Hz, 2H), 4.42 (t, *J* = 4.7 Hz, 2H), 3.69 (t, *J* = 4.7 Hz, 2H), 3.39 (q, *J* = 7.1 Hz, 6H), 1.23 (t, *J* = 7.1 Hz, 9H). ^13^C NMR (75 MHz, dimethylsulfoxide-*d*_6_) δ 157.2, 136.6, 131.6, 130.3, 128.8, 128.2, 128.0, 125.3, 120.1, 114.9, 61.1, 55.1, 52.9, 7.4.

#### Synthesis of (*E*)-*N*,*N*,*N*-Triethyl-2-(4-(3-(trifluoromethyl)styryl)phenoxy)ethan-1-aminium Iodide (**15**)

Obtained from **62** (47 mg, 0.112 mmol, 1 equiv) and triethylamine in toluene (1:1 *v*/*v*, 5 mL) at reflux for 16 h. Upon cooling to room temperature, diethyl ether was added and the solid was collected by filtration to give **15** as an off-white solid in a 32% yield. Mp = 178.6 °C. *R*_t_ (LC-MS) = 3.963 min. LC-MS (ESI): *m*/*z* calcd for C_23_H_29_F_3_NO [M]^+^ = 392.22, found 392.2. *R*_t_ (HPLC) = 14.72 min. ^1^H NMR (300 MHz, methanol-*d*_4_) δ 7.85–7.75 (m, 2H), 7.64–7.55 (m, 2H), 7.55–7.46 (m, 2H), 7.26 (d, *J* = 16.4 Hz, 1H), 7.14 (d, *J* = 16.4 Hz, 1H), 7.08–6.98 (m, 2H), 4.48 (t, *J* = 4.6 Hz, 2H), 3.83–3.74 (m, 2H), 3.50 (q, *J* = 7.2 Hz, 6H), 1.38 (t, *J* = 7.2 Hz, 9H). ^13^C NMR (75 MHz, methanol-*d*_4_) δ 158.9, 140.2, 132.3, 132.1 (q, *J* = 33.7 Hz), 131.0, 130.6 (q, *J* = 1.3 Hz), 130.5, 129.3, 126.6, 124.6 (q, *J* = 3.9 Hz), 123.9 (q, *J* = 3.8 Hz), 115.9, 62.7, 57.1, 55.0, 7.9.

#### Synthesis of (*E*)-*N*,*N*,*N*-Triethyl-2-(4-(4-(trifluoromethyl)styryl)phenoxy)ethan-1-aminium Iodide (**16**)

Obtained from (*E*)-*N*,*N*-diethyl-2-(4-(4-(trifluoromethyl)styryl)phenoxy)ethan-1-amine **55** (60 mg, 0.17 mmol, 1 equiv) and iodoethane (10 equiv) in THF (2 mL), according to Method B, at reflux temperature overnight. Upon cooling to room temperature, the reaction mixture was diluted with diethyl ether, and the resulting suspension was filtered, affording the desired compound **16** as a white solid in a 55% yield. Mp = 243.8 °C. *R*_t_ (LC-MS) = 3.999 min. LC-MS (ESI): *m*/*z* calcd for C_23_H_29_F_3_NO [M]^+^ = 392.22, found 392.3. *R*_t_ (HPLC) = 14.70 min. ^1^H NMR (300 MHz, dimethylsulfoxide-*d*_6_) δ 7.79 (d, *J* = 8.3 Hz, 2H), 7.71 (d, *J* = 8.3 Hz, 2H), 7.65 (d, *J* = 8.8 Hz, 2H), 7.41 (d, *J* = 16.5 Hz, 1H), 7.24 (d, *J* = 16.5 Hz, 1H), 7.04 (d, *J* = 8.8 Hz, 2H), 4.43 (t, *J* = 4.8 Hz, 2H), 3.69 (t, *J* = 4.8 Hz, 2H), 3.39 (q, *J* = 7.1 Hz, 6H), 1.24 (t, *J* = 7.1 Hz, 9H). ^13^C NMR (75 MHz, dimethylsulfoxide-*d*_6_) δ 157.5, 141.5, 130.8, 130.0, 128.3, 127.1 (q, *J* = 31.6 Hz), 126.7, 125.6 (q, *J* = 3.7 Hz), 125.0, 120.8 (q, *J* = 272.0 Hz), 115.0, 61.1, 55.1, 52.9, 7.3.

#### Synthesis of (*E*)-*N*,*N*,*N*-Triethyl-2-(4-(3-methoxystyryl)phenoxy)ethan-1-aminium Iodide (**17**)

Obtained from (*E*)-*N*,*N*-diethyl-2-(4-(3-methoxystyryl)phenoxy)ethan-1-amine **56** (20 mg, 0.02 mmol, 1 equiv) according to Method B, using iodoethane as a solvent (2 mL), at reflux temperature overnight. Upon cooling at room temperature, the reaction mixture was diluted with diethyl ether and the resulting suspension was filtered, affording the desired product **17** as a white solid in a 100% yield. Mp = 186 °C. *R*_t_ (LC-MS) = 3.736 min. LC-MS (ESI): *m*/*z* calcd for C_23_H_32_NO_2_ [M]^+^ = 354.24, found 354.3. *R*_t_ (HPLC) = 13.62 min. ^1^H NMR (300 MHz, methanol-*d*_4_) δ 7.55 (d, *J* = 8.8 Hz, 2H), 7.25 (t, *J* = 8.0 Hz, 1H), 7.17–7.04 (m, 4H), 7.00 (d, *J* = 8.8 Hz, 2H), 6.81 (ddd, *J* = 8.0, 2.5, 0.9 Hz, 1H), 4.52–4.40 (m, 2H), 3.82 (s, 3H), 3.80–3.74 (m, 2H), 3.49 (q, *J* = 7.2 Hz, 6H), 1.38 (t, *J* = 7.2 Hz, 9H). ^13^C NMR (75 MHz, methanol-*d*_4_) δ 161.5, 158.5, 140.4, 132.8, 130.6, 129.1, 129.0, 128.3, 120.0, 115.9, 114.0, 112.6, 62.6, 57.1, 55.7, 54.9, 7.9.

#### Synthesis of (*E*)-*N*,*N*,*N*-Triethyl-2-(4-(4-methoxystyryl)phenoxy)ethan-1-aminium Iodide (**18**)

Obtained from (*E*)-*N*,*N*-diethyl-2-(4-(4-methoxystyryl)phenoxy)ethan-1-amine **57** (100 mg, 0.31 mmol, 1 equiv) and iodoethane (10 equiv) in THF (5 mL), according to Method B, at reflux temperature, overnight. The reaction mixture was concentrated under vacuum, the residue was diluted with diethyl ether, and the resulting suspension was filtered, affording the desired compound **18** as a white solid in a 98% yield. Mp = 222.1 °C. *R*_t_ (LC-MS) = 3.723 min. LC-MS (ESI): *m*/*z* calcd for C_23_H_32_NO_2_ [M]^+^ = 354.24, found 354.3. *R*_t_ (HPLC) = 13.58 min. ^1^H NMR (300 MHz, methanol-*d*_4_) δ 7.50 (d, *J* = 8.8 Hz, 2H), 7.46 (d, *J* = 8.8 Hz, 2H), 7.02–6.97 (m, 4H), 6.90 (d, *J* = 8.8 Hz, 2H), 4.45 (t, *J* = 4.5 Hz, 2H), 3.80 (s, 3H), 3.79–3.74 (m, 2H), 3.49 (q, *J* = 7.2 Hz, 6H), 1.38 (t, *J* = 7.2 Hz, 9H). ^13^C NMR (75 MHz, methanol-*d*_4_) δ 160.7, 158.2, 133.3, 131.7, 128.63, 128.55, 128.0, 126.7, 115.8, 115.1, 62.6, 57.1, 55.7, 54.9, 7.9.

#### Synthesis of (*E*)-*N*,*N*,*N*-Triethyl-2-(4-(3-hydroxystyryl)phenoxy)ethan-1-aminium Iodide (**19**)

A solution of (*E*)-3-hydroxy-4′-(2-iodoethyloxy)stilbene **64** (85 mg, 0.23 mmol, 1 equiv) was dissolved in 3 mL of triethylamine and 3 mL of toluene and stirred at reflux temperature for 5 h. Upon cooling at room temperature, the suspension was filtered and washed with CH_3_CN obtaining the desired product **19** as a pale brown solid in a 23% yield. Mp = 196.3 °C. *R*_t_ (LC-MS) = 3.326 min. LC-MS (ESI): *m*/*z* calcd for C_22_H_30_NO_2_ [M]^+^ = 340.23, found 340.2. *R*_t_ (HPLC) = 11.97 min. ^1^H NMR (300 MHz, methanol-*d*_4_) δ 7.52 (d, *J* = 8.7 Hz, 2H), 7.15 (t, *J* = 7.8 Hz, 1H), 7.10–6.92 (m, 6H), 6.67 (dd, *J* = 7.8, 1.9 Hz, 1H), 4.50–4.39 (m, 2H), 3.80–3.71 (m, 2H), 3.48 (q, *J* = 7.2 Hz, 6H), 1.37 (t, *J* = 7.2 Hz, 9H). ^13^C NMR (75 MHz, methanol-*d*_4_) δ 158.8, 158.5, 140.4, 132.8, 130.6, 128.9, 128.8, 128.4, 119.1, 115.8, 115.5, 113.7, 62.6, 57.1, 54.9, 7.9.

#### Synthesis of (*E*)-*N*,*N*,*N*-Triethyl-2-(4-(4-hydroxystyryl)phenoxy)ethan-1-aminium Iodide (**20**)

A mixture of **67** (32 mg, 0.058 mmol) and an excess of a 1.25 M HCl solution in MeOH was stirred at reflux overnight. Upon cooling to room temperature, the resulting mixture was concentrated under vacuum to give **20** as a yellow solid in a 100% yield. Mp = 139.4–139.9 °C. *R*_t_ (LC-MS) = 3.301 min. LC-MS (ESI): *m*/*z* calcd for C_22_H_30_NO_2_ [M]^+^ = 340.23, found 340.3. *R*_t_ (HPLC) = 11.80 min. ^1^H NMR (300 MHz, methanol-*d*_4_) δ 7.54–7.43 (m, 2H), 7.41–7.32 (m, 2H), 7.04–6.86 (m, 4H), 6.82–6.71 (m, 2H), 4.44 (t, *J* = 4.6 Hz, 2H), 3.80–3.71 (m, 2H), 3.48 (q, *J* = 7.2 Hz, 6H), 1.38 (t, *J* = 7.2 Hz, 9H).^13^C NMR (75 MHz, methanol-*d*_4_) δ 158.3, 158.0, 133.4, 130.6, 128.7, 128.5, 128.3, 125.9, 116.5, 115.8, 62.6, 57.1, 54.9, 7.9.

#### Synthesis of (*E*)-2-(4-(3,5-Dihydroxystyryl)phenoxy)-*N*,*N*,*N*-triethylethan-1-aminium Iodide (**22**)

A solution of (*E*)-5-(4-(2-iodoethoxy)styryl)benzene-1,3-diol **77** (290 mg, 0.76 mmol, 1 equiv) was dissolved in 5 mL of triethylamine and 5 mL of toluene and stirred at reflux temperature for 5 h. Upon cooling at room temperature, the suspension was filtered and washed with CH_3_CN and EtOH providing the desired product **22** as a pale brown solid in a 12% yield. Mp = 236.5 °C. *R*_t_ (LC-MS) = 3.014 min. LC-MS (ESI): *m*/*z* calcd for C_22_H_30_NO_3_ [M]^+^ = 356.22, found 356.3. *R*_t_ (HPLC) = 10.52 min. ^1^H NMR (300 MHz, methanol-*d*_4_) δ 7.49 (d, *J* = 8.8 Hz, 2H), 7.06–6.95 (m, 3H), 6.88 (d, *J* = 16.3 Hz, 1H), 6.47 (d, *J* = 2.1 Hz, 2H), 6.19 (t, *J* = 2.1 Hz, 1H), 4.49–4.40 (m, 2H), 3.78–3.71 (m, 2H), 3.47 (q, *J* = 7.2 Hz, 6H), 1.37 (t, *J* = 7.2 Hz, 9H). ^13^C NMR (75 MHz, methanol-*d*_4_) δ 159.7, 158.4, 140.9, 132.8, 128.9, 128.6, 127.3, 115.8, 105.9, 102.9, 62.6, 57.1, 54.9, 7.9.

#### Synthesis of 2-(4-(Benzyloxy)phenoxy)-*N*,*N*,*N*-triethylethan-1-aminium Iodide (**23**)

Obtained from 2-(4-(benzyloxy)phenoxy)-*N*,*N*-diethylethan-1-amine **39** (280 mg, 0.94 mmol, 1 equiv) and iodoethane (4 equiv) in 1,2-dichloroethane (3 mL), according to Method B, at room temperature, overnight. The reaction mixture was concentrated under reduced pressure, the residue was triturated in diisopropyl ether/2-propanol, and then filtered, providing the desired compound **23** as a white solid in an 84% yield. Mp = 172.4–173.9 °C. *R*_t_ (LC-MS) = 3.515 min. LC-MS (ESI): *m*/*z* calcd for C_21_H_30_NO_2_ [M]^+^ = 328.23, found 328.3. *R*_t_ (HPLC) = 12.73 min. ^1^H NMR (300 MHz, methanol-*d*_4_) δ 7.47–7.25 (m, 5H), 7.00–6.90 (m, 4H), 5.03 (s, 2H), 4.40–4.33 (m, 2H), 3.76–3.69 (m, 2H), 3.47 (q, *J* = 7.2 Hz, 6H), 1.36 (t, *J* = 7.2 Hz, 9H). ^13^C NMR (75 MHz, methanol-*d*_4_) δ 155.2, 153.2, 138.8, 129.5, 128.8, 128.6, 117.1, 116.7, 71.6, 63.2, 57.3, 55.0, 8.0.

#### Synthesis of *N*,*N*,*N*-Triethyl-2-(4-(phenoxymethyl)phenoxy)ethan-1-aminium Iodide (**24**)

Obtained from *N*,*N*-diethyl-2-(4-(phenoxymethyl)phenoxy)ethan-1-amine **105** (100 mg, 0.33 mmol, 1 equiv) according to Method B, using iodoethane as a solvent (2 mL), at room temperature, overnight. The reaction mixture was diluted with diethyl ether, and the resulting suspension was filtered, providing the desired compound **24** as a white solid in a 50% yield. Mp = 153 °C. *R*_t_ (LC-MS) = 3.629 min. LC-MS (ESI): *m*/*z* calcd for C_21_H_30_NO_2_ [M]^+^ = 328.23, found 328.3. *R*_t_ (HPLC) = 12.69 min. ^1^H NMR (400 MHz, methanol-*d*_4_) δ 7.44–7.38 (m, 2H), 7.30–7.21 (m, 2H), 7.09–6.99 (m, 2H), 6.99–6.94 (m, 2H), 6.92 (td, *J* = 7.2, 1.1 Hz, 1H), 5.01 (s, 2H), 4.45 (t, *J* = 4.8 Hz, 2H), 3.79–3.73 (m, 2H), 3.48 (q, *J* = 7.2 Hz, 6H), 1.37 (t, *J* = 7.2 Hz, 9H). ^13^C NMR (100 MHz, methanol-*d*_4_) δ 160.2, 158.6, 132.2, 130.44, 130.43, 121.9, 116.0, 115.7, 70.5, 62.7, 57.2, 55.0, 8.0.

#### Synthesis of 2-(4-Benzamidophenoxy)-*N*,*N*,*N*-triethylethan-1-aminium Iodide (**25**)

Obtained from *N*-(4-(2-(diethylamino)ethoxy)phenyl)benzamide **91** (100 mg, 0.31 mmol, 1 equiv) according to Method B, using iodoethane (1 mL) as a solvent, at reflux temperature for 5 h. Upon cooling, the reaction mixture was diluted in DCM and the resulting suspension was filtered. The solid was washed repeatedly with DCM, providing the desired compound **25** as a white solid in a 46% yield. Mp = 202 °C. *R*_t_ (LC-MS) = 3.082 min. LC-MS (ESI): *m*/*z* calcd for C_21_H_29_N_2_O_2_ [M]^+^ = 341.22, found 341.2. *R*_t_ (HPLC) = 10.69 min. ^1^H NMR (300 MHz, dimethylsulfoxide-*d*_6_) δ 10.16 (s, 1H), 7.95 (d, *J* = 6.7 Hz, 2H), 7.72 (d, *J* = 9.0 Hz, 2H), 7.63–7.47 (m, 3H), 7.00 (d, *J* = 9.0 Hz, 2H), 4.39 (t, *J* = 4.8 Hz, 2H), 3.68 (t, *J* = 4.8 Hz, 2H), 3.38 (q, *J* = 7.1 Hz, 6H), 1.24 (t, *J* = 7.1 Hz, 9H). ^13^C NMR (75 MHz, dimethylsulfoxide-*d*_6_) δ 165.1, 153.6, 134.9, 133.0, 131.5, 128.4, 127.5, 121.9, 114.6, 61.2, 55.2, 52.9, 7.3.

#### Synthesis of *N*,*N*,*N*-Triethyl-2-(4-(phenylcarbamoyl)phenoxy)ethan-1-aminium Iodide (**26**)

Obtained from 4-(2-(diethylamino)ethoxy)-*N*-phenylbenzamide **87** (100 mg, 0.31 mmol, 1 equiv) according to Method B, using iodoethane (1 mL) as a solvent, at reflux temperature for 5 h. Upon cooling, the reaction mixture was diluted in DCM and the resulting suspension was filtered. The solid was washed repeatedly with DCM, providing the desired compound **26** as an off-white solid in a 46% yield. Mp = 192 °C. *R*_t_ (LC-MS) = 3.108 min. LC-MS (ESI): *m*/*z* calcd for C_21_H_29_N_2_O_2_ [M]^+^ = 341.22, found 341.3. *R*_t_ (HPLC) = 10.89 min. ^1^H NMR (300 MHz, dimethylsulfoxide-*d*_6_) δ 10.09 (d, *J* = 2.4 Hz, 1H), 8.01 (d, *J* = 8.8 Hz, 2H), 7.76 (d, *J* = 8.5 Hz, 2H), 7.40–7.26 (m, 2H), 7.20–7.05 (m, 3H), 4.50 (t, *J* = 4.8 Hz, 2H), 3.72 (t, *J* = 4.8 Hz, 2H), 3.40 (q, *J* = 7.2 Hz, 6H), 1.25 (t, *J* = 7.2 Hz, 9H). ^13^C NMR (75 MHz, dimethylsulfoxide-*d*_6_) δ 164.6, 160.0, 139.2, 129.6, 128.5, 127.7, 123.5, 120.4, 114.2, 61.3, 55.1, 52.9, 7.3.

#### Synthesis of (*E*)-*N*,*N*,*N*-Triethyl-2-(4-(phenyldiazenyl)phenoxy)ethan-1-aminium Iodide (**27**)

Obtained from **93** (218 mg, 733 mmol) and iodoethane (4 equiv) in DCM (2.5 mL) according to Method B at room temperature for 16 h. The reaction mixture was diluted with diethyl ether (5 mL). The suspension was stirred for 15 min, and then the solid was isolated by filtration. The solid was redissolved in the smallest amount of EtOH, diethyl ether was added, and the formed solid was isolated by filtration to give **27** as a yellow solid in a 32% yield. Mp = 152.2–155.0 °C. *R*_t_ (LC-MS) = 3.634 min. LC-MS (ESI): *m*/*z* calcd for C_20_H_28_N_3_O [M]^+^ = 326.22, found 326.2. *R*_t_ (HPLC) = 12.89 min. ^1^H NMR (300 MHz, methanol-*d*_4_) δ 7.99–7.91 (m, 2H), 7.91–7.82 (m, 2H), 7.59–7.42 (m, 3H), 7.23–7.14 (m, 2H), 4.56 (t, *J* = 4.6 Hz, 2H), 3.86–3.77 (m, 2H), 3.50 (q, *J* = 7.3 Hz, 6H), 1.39 (t, *J* = 7.3 Hz, 9H). ^13^C NMR (75 MHz, methanol-*d*_4_) δ 161.3, 154.0, 148.9, 131.9, 130.2, 125.8, 123.6, 116.2, 63.0, 57.1, 55.0, 8.0.

#### Synthesis of 2-(4-(Benzo[*d*]oxazol-2-yl)phenoxy)-*N*,*N*,*N*-triethylethan-1-aminium Iodide (**28**)

A solution of 2-(4-(benzo[*d*]oxazol-2-yl)phenoxy)-*N*,*N*-diethylethan-1-amine hydrochloride **107** (130 mg, 37 mmol, 1 equiv) in Na_2_CO_3_ 1 M (5 mL) was extracted with EtOAc (3 × 5 mL). The combined organic phases were dried over anhydrous Na_2_SO_4_, filtered, and the solvent was evaporated under reduced pressure. The resulting tertiary amine was reacted according to Method B, using iodoethane as a solvent (2 mL), at room temperature, overnight. The reaction mixture was diluted with diethyl ether, and the resulting suspension was filtered, providing the desired compound **28** as a white solid in a 90% yield. Mp = 213–218 °C (dec). *R*_t_ (LC-MS) = 3.386 min. LC-MS (ESI): *m*/*z* calcd for C_21_H_27_N_2_O_2_ [M]^+^ = 339.21, found 339.2. *R*_t_ (HPLC) = 12.29 min. ^1^H NMR (300 MHz, chloroform-*d*) δ 8.19 (d, *J* = 9.0 Hz, 2H), 7.74–7.69 (m, 1H), 7.57–7.52 (m, 1H), 7.35–7.30 (m, 2H), 7.10 (d, *J* = 9.0 Hz, 2H), 4.69–4.61 (m, 2H), 4.17–4.10 (m, 2H), 3.62 (q, *J* = 7.2 Hz, 6H), 1.50 (t, *J* = 7.2 Hz, 9H). ^13^C NMR (75 MHz, methanol-*d*_4_) δ 163.0, 160.5, 150.5, 141.4, 129.2, 125.0, 124.6, 120.1, 118.9, 115.0, 110.3, 61.5, 55.6, 53.6, 6.6.

#### Synthesis of 2-(4-(1*H*-Benzo[*d*]imidazol-2-yl)phenoxy)-*N*,*N*,*N*-triethylethan-1-aminium Iodide (**29**)

Obtained from 2-(4-(1*H*-benzo[*d*]imidazol-2-yl)phenoxy)-*N*,*N*-diethylethan-1-amine **108** (100 mg, 0.32 mmol, 1 equiv) and iodoethane (8 equiv) in 1,2-dichloroethane, according to Method B, at room temperature, overnight. The volatiles were removed under reduced pressure, and the residue was diluted with diethyl ether. The resulting suspension was filtered, and the desired product **29** was obtained as an off-white solid in a 50% yield. Mp = 176.2–180.1 °C. *R*_t_ (LC-MS) = 2.525 min. LC-MS (ESI): *m*/*z* calcd for C_21_H_28_N_3_O [M]^+^ = 338.22, found 338.2. *R*_t_ (HPLC) = 8.24 min. ^1^H NMR (300 MHz, methanol-*d*_4_) δ 8.09 (d, *J* = 8.9 Hz, 2H), 7.62 (dd, *J* = 6.1, 3.2 Hz, 2H), 7.30 (dd, *J* = 6.1, 3.2 Hz, 2H), 7.22 (d, *J* = 8.9 Hz, 2H), 4.61–4.49 (m, 2H), 3.86–3.77 (m, 2H), 3.51 (q, *J* = 7.2 Hz, 6H), 1.39 (t, *J* = 7.2 Hz, 9H). ^13^C NMR (75 MHz, methanol-*d*_4_) δ 161.0, 152.7, 139.1, 129.8, 124.4, 123.5, 116.5, 115.5, 63.0, 57.1, 55.0, 8.0.

#### Synthesis of 2-(4-(1*H*-Indol-6-yl)phenoxy)-*N*,*N*,*N*-triethylethan-1-aminium Iodide (**30**)

Obtained from 2-(4-(1*H*-indol-6-yl)phenoxy)-*N*,*N*-diethylethan-1-amine **95** (19 mg, 0.06 mmol, 1 equiv) and iodoethane (50 equiv) in THF (5 mL) according to Method B at reflux temperature overnight. Upon cooling, the reaction mixture was diluted with diisopropyl ether, and the resulting suspension was filtered, providing the desired compound **30** as an off-white solid in a 56% yield. Mp = 206.3–207.4 °C. *R*_t_ (LC-MS) = 3.464 min. LC-MS (ESI): *m*/*z* calcd for C_22_H_29_N_2_O [M]^+^ = 337.23, found 337.3. *R*_t_ (HPLC) = 12.51 min. ^1^H NMR (300 MHz, methanol-*d*_4_) δ 7.63 (d, *J* = 8.9 Hz, 2H), 7.60–7.53 (m, 2H), 7.28–7.21 (m, 2H), 7.07 (d, *J* = 8.9 Hz, 2H), 6.44 (dd, *J* = 3.1, 0.9 Hz, 1H), 4.52–4.42 (m, 2H), 3.82–3.72 (m, 2H), 3.49 (q, *J* = 7.2 Hz, 6H), 1.38 (t, *J* = 7.2 Hz, 9H). ^13^C NMR (150 MHz, methanol-*d*_4_) δ 157.8, 138.3, 137.9, 135.2, 129.3, 128.7, 126.3, 121.5, 119.5, 115.9, 110.1, 102.2, 62.7, 57.2, 55.0, 7.9.

#### Synthesis of 2-(4-(1*H*-Indol-5-yl)phenoxy)-*N*,*N*,*N*-triethylethan-1-aminium Iodide (**31**)

Obtained from **98** (40 mg, 0.13 mmol, 1 equiv) and iodoethane (50 equiv) in THF (5 mL) according to Method B at reflux temperature overnight. Upon cooling, the reaction mixture was diluted with diisopropyl ether, and the resulting suspension was filtered, providing **31** as an off-white solid in a 20% yield. Mp = 203 °C dec. *R*_t_ (LC-MS) = 3.414 min. LC-MS (ESI): *m*/*z* calcd for C_22_H_29_N_2_O [M]^+^ = 337.23, found 337.2. *R*_t_ (HPLC) = 12.32 min. ^1^H NMR (300 MHz, methanol-*d*_4_) δ 7.72 (dd, *J* = 1.8, 0.7 Hz, 1H), 7.65–7.56 (m, 2H), 7.43 (dt, *J* = 8.4, 0.8 Hz, 1H), 7.33 (dd, *J* = 8.5, 1.8 Hz, 1H), 7.25 (d, *J* = 3.1 Hz, 1H), 7.09–7.02 (m, 2H), 6.48 (dd, *J* = 3.1, 0.9 Hz, 1H), 4.50–4.44 (m, 2H), 3.82–3.67 (m, 2H), 3.50 (q, *J* = 7.2 Hz, 6H), 1.39 (t, *J* = 7.2 Hz, 9H). ^13^C NMR (75 MHz, methanol-*d*_4_) δ 157.6, 138.4, 133.1, 130.1, 129.3, 127.3, 126.3, 121.7, 119.2, 115.9, 112.4, 102.7, 62.8, 57.5, 55.2, 7.9.

#### Synthesis of 2-(4-(Benzofuran-5-yl)phenoxy)-*N*,*N*,*N*-triethylethan-1-aminium Iodide (**32**)

A solution of 2-(4-(benzofuran-5-yl)phenoxy)-*N*,*N*-diethylethan-1-amine hydrochloride (48 mg, 0.14 mmol, 1 equiv) in DCM was washed with a solution of 1 M NaOH and with brine. The organic phase was dried over anhydrous Na_2_SO_4_, filtered, and evaporated under vacuum to afford the corresponding free base. The resulting residue was reacted with iodoethane (50 equiv) in THF (5 mL) according to Method B at reflux temperature overnight. Upon cooling, the reaction mixture was diluted with diethyl ether and the resulting suspension was filtered, providing the desired compound **32** as an off-white solid in a 22% yield. Mp = 253 °C. *R*_t_ (LC-MS) = 3.731 min. LC-MS (ESI): *m*/*z* calcd for C_22_H_28_NO_2_ [M]^+^ = 338.21, found 338.2. *R*_t_ (HPLC) = 13.07 min. ^1^H NMR (300 MHz, methanol-*d*_4_) δ 7.80–7.78 (m, 1H), 7.77 (dd, *J* = 2.2, 0.8 Hz, 1H), 7.61 (d, *J* = 8.8 Hz, 2H), 7.56–7.47 (m, 2H), 7.09 (d, *J* = 8.9 Hz, 2H), 6.88 (dd, *J* = 2.2, 1.0 Hz, 1H), 4.53–4.45 (m, 2H), 3.82–3.76 (m, 2H), 3.50 (q, *J* = 7.2 Hz, 6H), 1.39 (t, *J* = 7.2 Hz, 9H). ^13^C NMR (75 MHz, methanol-*d*_4_) δ 158.2, 155.8, 147.1, 137.1, 136.8, 129.6, 129.5, 124.6, 120.2, 116.0, 112.3, 107.8, 62.7, 57.2, 55.0, 8.0.

#### Synthesis of (*R*)-3-(4-(1*H*-Indol-5-yl)phenoxy)-1,1-dimethylpyrrolidin-1-ium Iodide (**33**)

Compound **100** (65 mg, 0.22 mmol) was dissolved in THF (5 mL). Iodomethane (277 μL, 4.45 mmol) was added, and the reaction mixture was added at 40 °C for 16 h. Upon cooling to room temperature, diethyl ether was added and the solid was isolated by vacuum filtration, washed with diethyl ether, and dried to give **33** as a white solid in a 93% yield. Mp = 226.1–228.7 °C. [α]_D_^25^ = −9.86 (*c* 0.5, dimethylsulfoxide). *R*_t_ (LC-MS) = 3.205 min. LC-MS (ESI): *m*/*z* calcd for C_20_H_23_N_2_O [M]^+^ = 307.18, found 307.2. *R*_t_ (HPLC) = 11.51 min. ^1^H NMR (300 MHz, dimethylsulfoxide-*d*_6_) δ 11.11 (s, 1H), 7.75 (dd, *J* = 1.8, 0.8 Hz, 1H), 7.62 (d, *J* = 8.8 Hz, 2H), 7.45 (dt, *J* = 8.4, 0.8 Hz, 1H), 7.39–7.31 (m, 2H), 7.04 (d, *J* = 8.8 Hz, 2H), 6.46 (ddd, *J* = 3.0, 1.9, 0.8 Hz, 1H), 5.31 (d, *J* = 7.6 Hz, 1H), 3.93 (dd, *J* = 13.2, 5.9 Hz, 1H), 3.87–3.75 (m, 2H), 3.70–3.56 (m, 1H), 3.27 (s, 3H), 3.22 (s, 3H), 2.92–2.74 (m, 1H), 2.39–2.24 (m, 1H). ^13^C NMR (75 MHz, dimethylsulfoxide-*d*_6_) δ 154.9, 135.5, 135.2, 130.8, 128.2, 127.9, 126.0, 120.1, 117.6, 115.9, 111.7, 101.4, 74.9, 69.3, 64.1, 52.6, 52.4, 30.1.

#### Synthesis of *N*,*N*-Diethyl-2-phenoxyethan-1-amine (**34**)

Obtained from phenol (1.00 g, 10.63 mmol), K_2_CO_3_ (2.5 equiv), KI (0.1 equiv), and 2-(diethylamino)ethyl chloride hydrochloride (1.5 equiv) in acetone (15 mL) according to Method A. The crude was diluted with diethyl ether and washed three times with a 1 M NaOH solution. The organic phase was dried over anhydrous Na_2_SO_4_ and filtered. The filtrate was evaporated under vacuum, providing the desired product **34** as a colorless oil in a 75% yield. *R*_f_ = 0.6 (DCM/MeOH 95:5 + 0.5% NH_3(aq20%)_). ^1^H NMR (300 MHz, chloroform-*d*) δ 7.34–7.24 (m, 2H), 7.03–6.85 (m, 3H), 4.06 (t, *J* = 6.4 Hz, 2H), 2.89 (t, *J* = 6.4 Hz, 2H), 2.66 (q, *J* = 7.2 Hz, 4H), 1.08 (t, *J* = 7.2 Hz, 6H).

#### Synthesis of 4-(2-Bromoethoxy)-1,1′-biphenyl (**35**)

Obtained from 4-phenylphenol (2.00 g, 11.75 mmol, 1 equiv), K_2_CO_3_ (2.5 equiv), KI (0.1 equiv), and 1,2-dibromoethane (4.2 equiv), according to Method A, at reflux temperature for 48 h. The residue was suspended in chloroform (100 mL) and washed with an aqueous solution of 10% NaOH (2 × 20 mL). The organic layer was dried over anhydrous Na_2_SO_4_, filtered, and the solvent was evaporated under reduced pressure. The crude was purified by silica gel flash column chromatography (cyclohexane/EtOAc 95:5) providing the desired product **35** as a white powder in a 46% yield. *R*_f_ = 0.57 (cyclohexane/EtOAc 9:1) mp = 112 °C (coherent with the literature^15^). ^1^H NMR (300 MHz, chloroform-*d*) δ 7.56 (d, *J* = 8.4 Hz, 2H), 7.49 (d, *J* = 8.1 Hz, 2H), 7.40–7.45 (m, 2H), 7.30–7.38 (m, 1H), 6.92 (d, *J* = 8.1 Hz, 2H), 4.30 (t, *J* = 6.3 Hz, 2H) 3.65 (t, *J* = 6.3 Hz, 2H).

#### Synthesis of 4-(2-Iodoethoxy)-1,1′-biphenyl (**36**)

Obtained from 4-(2-bromoethoxy)-1,1′-biphenyl **35** (1.50 g, 5.44 mmol, 1 equiv) according to Method D, providing the desired compound **36** as a white solid in a 92% yield. *R*_f_ = 0.51 (cyclohexane/EtOAc 9:1). ^1^H NMR (300 MHz, chloroform-*d*) δ 7.56 (d, *J* = 8.4 Hz, 2H), 7.49 (d, *J* = 8.1 Hz, 2H), 7.40–7.45 (m, 2H), 7.30–7.38 (m, 1H), 6.92 (d, *J* = 8.1 Hz, 2H), 4.30 (t, *J* = 6.3 Hz, 2H) 3.45 (t, *J* = 6.3 Hz, 2H).

#### Synthesis of 2-([1,1′-Biphenyl]-4-yloxy)-*N*,*N*-diethylethan-1-amine (**37**)

Obtained from 4-(2-iodoethoxy)-1,1′-biphenyl **36** (900 mg, 2.79 mmol, 1 equiv) according to Method E at 60 °C for 4 h. The residue was purified by silica gel flash column chromatography (DCM/MeOH 98:2 + 0.5% NH_3(aq20%)_) providing the desired compound **37** as a pale-yellow oil in an 84% yield. *R*_f_ = 0.42 (DCM/MeOH 98:2 + 0.5% NH_3(aq20%)_). ^1^H NMR (300 MHz, chloroform-*d*) δ 7.56 (d, *J* = 8.4 Hz, 2H), 7.49 (d, *J* = 8.1 Hz, 2H), 7.40–7.45 (m, 2H), 7.30–7.38 (m, 1H), 6.92 (d, *J* = 8.1 Hz, 2H), 4.12 (t, *J* = 6.3 Hz, 2H), 2.93 (t, *J* = 6.3 Hz, 2H), 2.69 (q, *J* = 7.1 Hz, 4H), 1.09 (t, *J* = 7.1 Hz, 6H).

#### Synthesis of 4-(Benzyloxy)phenol (**38**)

Under a nitrogen atmosphere, a suspension of hydroquinone (1.00 g, 9.08 mmol, 1 equiv) and K_2_CO_3_ (0.62 g, 4.54 mmol, 0.5 equiv) in acetone (10 mL) was vigorously stirred at reflux temperature for 30 min. A solution of benzyl bromide (0.78 g, 4.54 mmol) in acetone (0.5 mL) was added dropwise, and the resulting mixture was refluxed overnight. The reaction mixture was cooled to room temperature, and the solid was removed by filtration. The filtrate was concentrated under vacuum, and the residue was diluted with EtOAc and washed with water. The organic phase was dried over anhydrous Na_2_SO_4_, filtered, and the filtrate was evaporated under vacuum. The crude was purified by silica gel flash chromatography (cyclohexane/EtOAc 85:15), providing the desired product **38** as a white solid in a 52% yield. *R*_f_ = 0.45 (cyclohexane/EtOAc 8:2). Mp = 123 °C (coherent with the literature^16^). ^1^H NMR (300 MHz, chloroform-*d*) δ 7.47–7.29 (m, 5H), 6.86 (d, *J* = 9.0 Hz, 2H), 6.76 (d, *J* = 9.0 Hz, 2H), 5.01 (s, 2H).

#### Synthesis of 2-(4-(Benzyloxy)phenoxy)-*N*,*N*-diethylethan-1-amine (**39**)

Obtained from 4-(benzyloxy)phenol **38** (250 mg, 1.25 mmol, 1 equiv), K_2_CO_3_ (2.5 equiv), KI (0.1 equiv), and 2-chloro-*N*,*N*-diethylethylamine hydrochloride (1.5 equiv) in acetone according to Method A. The crude was diluted with diethyl ether and washed three times with a 1 M NaOH solution. The organic phase was dried over anhydrous Na_2_SO_4_ and filtered. The filtrate was evaporated under vacuum, providing the desired product **39** as a colorless oil in a 75% yield. *R*_f_ = 0.58 (DCM/MeOH 95:5 + 0.5% NH_3(20% aq)_). ^1^H NMR (300 MHz, chloroform-*d*) δ 7.52–7.26 (m, 5H), 6.98–6.70 (m, 4H), 5.01 (s, 2H), 4.02 (t, *J* = 6.3 Hz, 2H), 2.87 (t, *J* = 6.3 Hz, 2H), 2.66 (q, *J* = 7.2 Hz, 4H), 1.08 (t, *J* = 7.2 Hz, 6H).

#### Synthesis of 4-Formylphenyl Acetate (**40**)

A solution of 4-hydroxybenzaldehyde (3.00 g, 24.6 mmol, 1 equiv) in pyridine (20 mL) was stirred at 0 °C for 30 min. Upon the dropwise addition of acetic anhydride (3.5 mL, 37 mmol, 1.5 equiv) for 30 min, the reaction mixture was warmed to room temperature and stirred until TLC showed full conversion. Afterward, the pH was adjusted to 7 by the dropwise addition of 1 M HCl (10 mL), and the product was extracted in diethyl ether. The combined organic phases were washed with 1 M HCl and 1 M NaOH and then dried over anhydrous Na_2_SO_4_, filtered under reduced pressure, and evaporated, providing the desired product **40** as a pale-yellow oil in an 80% yield. *R*_f_ = 0.43 (cyclohexane/EtOAc 9:1). ^1^H NMR (300 MHz, chloroform-*d*) δ 9.9 (s, 1H) 7.8 (d, *J* = 8.5 Hz, 2H) 7.30 (d, *J* = 8.5 Hz, 2H) 2.30 (s, 3H).

#### Synthesis of 4-Vinylphenyl Acetate (**41**)

Under an inert atmosphere, methyltriphenylphosphonium bromide (7.16 g, 20.04 mmol, 1 equiv) was added portionwise to a suspension of 4-formylphenyl acetate **40** (2.74 g, 16.7 mmol, 1.2 equiv) and K_2_CO_3_ (2.76 g, 20.04 mmol, 1.2 equiv) in anhydrous THF (35 mL). The reaction mixture was refluxed for 6 h and then concentrated under reduced pressure. The residue was diluted with diethyl ether and washed with water. The water layer was re-extracted with diethyl ether, and the combined organic phases were dried over anhydrous Na_2_SO_4_, filtered, and evaporated under reduced pressure. The crude was purified by silica gel flash column chromatography (cyclohexane/EtOAc 95:5), providing the desired compound **41** as a colorless oil in a 62% yield. *R*_f_ = 0.57 (cyclohexane/EtOAc 9:1). ^1^H NMR (300 MHz, chloroform-*d*) δ 7.42 (d, *J* = 8.7 Hz, 2H), 7.06 (d, *J* = 8.7 Hz, 2H), 6.71 (dd, *J* = 17.6, 10.9 Hz, 1H), 5.71 (dd, *J* = 17.6, 0.7 Hz, 1H), 5.25 (dd, *J* = 10.9, 0.7 Hz, 1H), 2.30 (s, 3H).

#### Synthesis of 4-Vinylphenol (**42**)

A solution of 4-vinylphenyl acetate **41** (3.00 g, 18.5 mmol, 1 equiv) in THF (30 mL) was cooled to 0 °C. A solution of 5 M NaOH (9 mL, 46.25 mmol, 2.5 equiv) was added dropwise for 5 min, and the reaction mixture was stirred at the same temperature for 4 h. The mixture was quenched for 15 min by the dropwise addition of cold 1.5 M HCl (30 mL) and then further diluted with 60 mL of cold water. The aqueous phase was extracted four times with diethyl ether, and the combined organic phases were dried over anhydrous Na_2_SO_4_, filtered, and concentrated by a rotary evaporator at 25 °C. The residue was taken in absolute EtOH (30 mL) and evaporated again at 25 °C, providing the desired compound **42** as a solid in a 100% yield. The compound was stored as an ethanolic solution at 0 °C to avoid polymerization. *R*_f_ = 0.45 (cyclohexane/EtOAc 8:2). Mp = 72–74 °C (coherent with the literature^17^). ^1^H NMR (300 MHz, chloroform-*d*) δ 7.30 (d, *J* = 8.5 Hz, 2H) 6.80 (d, *J* = 8.5 Hz, 2H) 6.70 (dd, *J* = 10.9, 17.6 Hz, 1H), 5.60 (d, *J* = 17.6 Hz, 1H) 5.10 (d, *J* = 10.9 Hz, 1H) 4.70 (s, OH, exchange with D_2_O).

#### Synthesis of 1-(2-Bromoethoxy)-4-vinylbenzene (**43**)

Obtained from 4-vinylphenol **42** (1.15 g, 9.6 mmol, 1 equiv), K_2_CO_3_ (2.5 equiv), KI (0.1 equiv), and dibromoethane (4.2 equiv) in anhydrous methyl ethyl ketone (20 mL), according to Method A, at reflux temperature for 48 h. The residue was resuspended in chloroform (100 mL) and washed with an aqueous solution of 10% NaOH. The organic layer was dried over anhydrous Na_2_SO_4_, filtered, and evaporated under reduced pressure. The crude was purified by silica gel flash column chromatography (cyclohexane/EtOAc 95:5) providing the desired product **43** as a pale-yellow oil in a 55% yield. *R*_f_ = 0.63 (cyclohexane/EtOAc 9:1). ^1^H NMR (300 MHz, chloroform-*d*) δ 7.39–7.30 (m, 2H), 6.92–6.82 (m, 2H), 6.66 (dd, *J* = 17.6, 10.9 Hz, 1H), 5.62 (d, *J* = 17.6 Hz, 1H), 5.14 (d, *J* = 10.9 Hz, 1H), 4.30 (t, *J* = 6.3 Hz, 2H), 3.63 (t, *J* = 6.3 Hz, 2H).

#### Synthesis of 1-(2-Iodoethoxy)-4-vinylbenzene (**44**)

Obtained from 1-(2-bromoethoxy)-4-vinylbenzene **43** (500 mg, 2.20 mmol, 1 equiv) according to Method D, providing the desired intermediate **44** as a pale-yellow oil in an 80% yield. *R*_f_ = 0.58 (cyclohexane/EtOAc 95:5). ^1^H NMR (300 MHz, chloroform-*d*) δ 7.35 (d, *J* = 8.9 Hz, 2H), 6.86 (d, *J* = 8.9 Hz, 2H), 6.66 (dd, *J* = 17.6, 10.9 Hz, 1H), 5.62 (d, *J* = 17.6 Hz, 1H), 5.14 (d, *J* = 10.9 Hz, 1H), 4.36–4.18 (m, 2H), 3.50–3.32 (m, 2H).

#### Synthesis of *N*,*N*-Diethyl-2-(4-vinylphenoxy)ethan-1-amine (**45**)

Obtained from 1-(2-iodoethoxy)-4-vinylbenzene **44** (370 mg, 1.35 mmol, 1 equiv) according to Method E, at 60 °C for 4 h, as a pale-yellow oil, in a 98% yield. *R*_f_ = 0.75 (DCM/MeOH 95:5 + 0.5% NH_3(aq. 20%)_). ^1^H NMR (300 MHz, chloroform-*d*) δ 7.33 (d, *J* = 8.7 Hz, 2H), 6.86 (d, *J* = 8.7 Hz, 2H), 6.65 (dd, *J* = 17.6, 10.9 Hz, 1H), 5.60 (d, *J* = 17.6 Hz, 1H), 5.12 (d, *J* = 10.9 Hz, 1H), 4.07 (t, *J* = 6.3 Hz, 2H), 2.90 (t, *J* = 6.3 Hz, 2H), 2.67 (q, *J* = 7.1 Hz, 4H), 1.09 (t, *J* = 7.1 Hz, 6H).

#### Synthesis of (*E*)-4-(2-Bromostyryl)phenol (**46**)

Obtained from 1-bromo-2-iodobenzene (500 mg, 1.77 mmol, 1 equiv) and 4-vinylphenol **42** (1.2 equiv) according to Method F. The crude was purified by silica gel flash column chromatography (from cyclohexane/EtOAc 9:1 to 7:3), providing the desired product **46** as a white solid in a 31% yield. *R*_f_ = 0.32 (cyclohexane/EtOAc 9:1). Mp = 115.3 °C. ^1^H NMR (300 MHz, chloroform-*d*) δ 7.64 (dd, *J* = 7.7, 1.6 Hz, 1H), 7.57 (dd, *J* = 7.9, 1.3 Hz, 1H), 7.45 (d, *J* = 8.6 Hz, 2H), 7.35–7.26 (m, 2H), 7.09 (ddd, *J* = 7.9, 7.3, 1.6 Hz, 1H), 6.98 (d, *J* = 16.2 Hz, 1H), 6.85 (d, *J* = 8.6 Hz, 2H), 5.02 (s, 1H).

#### Synthesis of (*E*)-4-(3-Bromostyryl)phenol (**47**)

Obtained from 1-bromo-3-iodobenzene (500 mg, 1.77 mmol, 1 equiv) and 4-vinylphenol **42** (1.2 equiv) according to Method F. The crude was purified by silica gel flash column chromatography (from cyclohexane/EtOAc 9:1 to 7:3), providing the desired product **47** as a pale-yellow solid in a 53% yield. *R*_f_ = 0.27 (cyclohexane/EtOAc 9:1). Mp = 133.3 °C (coherent with the literature^18^). ^1^H NMR (300 MHz, chloroform-*d*) δ 7.63 (t, *J* = 1.8 Hz, 1H), 7.40 (d, *J* = 8.3 Hz, 2H), 7.38–7.32 (m, 2H), 7.20 (t, *J* = 7.9 Hz, 1H), 7.04 (d, *J* = 16.3 Hz, 1H), 6.91–6.80 (m, 3H), 5.00 (s, 1H, exchanges with D_2_O).

#### Synthesis of (*E*)-4-(4-Bromostyryl)phenol (**48**)

Obtained from 1-bromo-4-iodobenzene (500 mg, 1.77 mmol, 1 equiv) and 4-vinylphenol **42** (1.2 equiv) according to Method F. The crude was purified by silica gel flash column chromatography (from cyclohexane/EtOAc 9:1 to 7:3), providing the desired product **48** as a white solid in a 66% yield. *R*_f_ = 0.29 (cyclohexane/EtOAc 9:1). Mp = 191.7 °C (coherent with the literature^19^). ^1^H NMR (300 MHz, chloroform-*d*) δ 7.48–7.30 (m, 6H), 7.03 (d, *J* = 16.4 Hz, 1H), 6.93–6.79 (m, 3H), 4.98 (s, 1H, exchanges with D_2_O).

#### Synthesis of (*E*)-4-(4-(Trifluoromethyl)styryl)phenol (**49**)

Obtained from 4-iodobenzotrifluoride (300 mg, 1.1 mmol, 1 equiv) and 4-vinylphenol **42** (1.2 equiv) according to Method F. The crude was purified by silica gel flash column chromatography (gradient from cyclohexane/EtOAc 9:1 to 7:3), providing the desired compound **49** as a white solid in a 54% yield. *R*_f_ = 0.84 (cyclohexane/EtOAc 8:2 + 1% triethylamine). Mp = 158.3–161.0 °C (coherent with the literature^20^). ^1^H NMR (300 MHz, chloroform-*d*) δ 7.62–7.54 (m, 4H), 7.44 (d, *J* = 8.8 Hz, 2H), 7.13 (d, *J* = 16.3 Hz, 1H), 6.97 (d, *J* = 16.3 Hz, 1H), 6.85 (d, *J* = 8.8 Hz, 2H), 4.88 (broad s, 1H, exchanges with D_2_O).

#### Synthesis of (*E*)-4-(3-Methoxystyryl)phenol (**50**)

Obtained from 1-iodo-3-methoxybenzene (200 mg, 0.86 mmol, 1 equiv) and 4-vinylphenol **42** (1.5 equiv) according to Method F. The crude was purified by silica gel flash column chromatography (DCM), providing product **50** as a beige solid in a 35% yield. *R*_f_ = 0.36 (cyclohexane/EtOAc 8:2). Mp = 117.6 °C (coherent with the literature^21^). ^1^H NMR (300 MHz, methanol-*d*_4_) δ 7.39 (d, *J* = 8.6 Hz, 2H), 7.22 (t, *J* = 7.9 Hz, 1H), 7.11–7.00 (m, 3H), 6.93 (d, *J* = 16.3 Hz, 1H), 6.81–6.73 (m, 3H), 3.81 (s, 3H).

#### Synthesis of (*E*)-4-(4-Methoxystyryl)phenol (**51**)

Obtained from 1-iodo-4-methoxybenzene (500 mg, 2.14 mmol, 1 equiv) and 4-vinylphenol **42** (1.5 equiv) according to Method F. The crude was purified by crystallization from MeOH, providing product **51** as a white solid in a 21% yield. *R*_f_ = 0.39 (cyclohexane/EtOAc 8:2). Mp = 136–138 °C (coherent with the literature^22^). ^1^H NMR (300 MHz, methanol-*d*_4_) δ 7.42 (d, *J* = 8.8 Hz, 2H), 7.35 (d, *J* = 8.8 Hz, 2H), 6.94–6.86 (m, 4H), 6.76 (d, *J* = 8.8 Hz, 2H), 3.80 (s, 3H).

#### Synthesis of (*E*)-2-(4-(2-Bromostyryl)phenoxy)-*N*,*N*-diethylethan-1-amine (**52**)

Obtained from (*E*)-4-(2-bromostyryl)phenol **46** (140 mg, 0.51 mmol, 1 equiv), K_2_CO_3_ (3 equiv), KI (0.1 equiv), and 2-chloro-*N*,*N*-diethylethylamine hydrochloride (1.5 equiv) in methyl ethyl ketone (10 mL) according to Method A. The residue was diluted with EtOAc, washed with water, and then extracted with 1 M HCl. The aqueous phase was basified with 1 M NaOH until pH 12 and then extracted with EtOAc (3 × 25 mL). The combined organic phases were dried over anhydrous Na_2_SO_4_, filtered, and the solvent was evaporated under reduced pressure providing the desired compound **52** as a yellow oil in a 100% yield. *R*_f_ = 0.24 (cyclohexane/EtOAc 9:1 + 1% triethylamine). ^1^H NMR (300 MHz, chloroform-*d*) δ 7.64 (dd, *J* = 7.9, 1.7 Hz, 1H), 7.57 (dd, *J* = 8.0, 1.3 Hz, 1H), 7.48 (d, *J* = 8.8 Hz, 2H), 7.36–7.26 (m, 2H), 7.09 (ddd, *J* = 8.0, 7.3, 1.7 Hz, 1H), 6.98 (d, *J* = 16.2 Hz, 1H), 6.91 (d, *J* = 8.8 Hz, 2H), 4.08 (t, *J* = 6.3 Hz, 2H), 2.89 (t, *J* = 6.3 Hz, 2H), 2.65 (q, *J* = 7.1 Hz, 4H), 1.08 (t, *J* = 7.1 Hz, 6H).

#### Synthesis of (*E*)-2-(4-(3-Bromostyryl)phenoxy)-*N*,*N*-diethylethan-1-amine (**53**)

Obtained from (*E*)-4-(3-bromostyryl)phenol **47** (240 mg, 0.87 mmol, 1 equiv), K_2_CO_3_ (3 equiv), KI (0.1 equiv), and 2-chloro-*N*,*N*-diethylethylamine hydrochloride (1.5 equiv) in methyl ethyl ketone (10 mL), according to Method A. The residue was diluted with EtOAc, washed with water, and then extracted with 1 M HCl. The aqueous phase was basified with 1 M NaOH until pH 12 and then extracted with EtOAc (3 × 25 mL). The combined organic phases were dried over anhydrous Na_2_SO_4_, filtered, and the solvent was evaporated under reduced pressure providing the desired compound **53** as a yellow oil in an 89% yield. *R*_f_ = 0.24 (cyclohexane/EtOAc 9:1 + 1% triethylamine). ^1^H NMR (300 MHz, chloroform-*d*) δ 7.63 (t, *J* = 1.8 Hz, 1H), 7.43 (d, *J* = 8.4 Hz, 2H), 7.40–7.32 (m, 2H), 7.20 (t, *J* = 7.8 Hz, 1H), 7.05 (d, *J* = 16.3 Hz, 1H), 6.94–6.83 (m, 3H), 4.08 (t, *J* = 6.3 Hz, 2H), 2.89 (t, *J* = 6.3 Hz, 2H), 2.65 (q, *J* = 7.1 Hz, 4H), 1.08 (t, *J* = 7.1 Hz, 6H).

#### Synthesis of (*E*)-2-(4-(4-Bromostyryl)phenoxy)-*N*,*N*-diethylethan-1-amine (**54**)

Obtained from (*E*)-4-(4-bromostyryl)phenol **48** (300 mg, 1.09 mmol, 1 equiv), K_2_CO_3_ (3 equiv), KI (0.1 equiv), and 2-chloro-*N*,*N*-diethylethylamine hydrochloride (1.2 equiv) in methyl ethyl ketone (10 mL), according to Method A. The residue was diluted with EtOAc, washed with water, and then extracted with 1 M HCl. The aqueous phase was basified with 1 M NaOH until pH 12 and then extracted with EtOAc (3 × 5 mL). The combined organic phases were dried over anhydrous Na_2_SO_4_, filtered, and the solvent was evaporated under reduced pressure providing the desired compound **54** as a yellow oil in an 80% yield. *R*_f_ = 0.24 (cyclohexane/EtOAc 9:1 + 1% triethylamine). ^1^H NMR (300 MHz, chloroform-*d*) δ 7.48–7.40 (m, 4H), 7.34 (d, *J* = 8.4 Hz, 2H), 7.04 (d, *J* = 16.3 Hz, 1H), 6.95–6.83 (m, 3H), 4.07 (t, *J* = 6.3 Hz, 2H), 2.88 (t, *J* = 6.3 Hz, 2H), 2.65 (q, *J* = 7.1 Hz, 4H), 1.08 (t, *J* = 7.1 Hz, 6H).

#### Synthesis of (*E*)-*N*,*N*-Diethyl-2-(4-(4-(trifluoromethyl)styryl)phenoxy)ethan-1-amine (**55**)

Obtained from (*E*)-4-(4-(trifluoromethyl)styryl)phenol **49** (150 mg, 0.57 mmol, 1 equiv), K_2_CO_3_ (3 equiv), KI (0.1 equiv), and 2-chloro-*N*,*N*-diethylethylamine hydrochloride (1.2 equiv) in methyl ethyl ketone (5 mL), according to Method A. The residue was diluted with EtOAc, washed with water, and then extracted with 1 M HCl. The aqueous phase was basified with 1 M NaOH until pH 12 and then extracted with EtOAc (3 × 20 mL). The combined organic phases were dried over anhydrous Na_2_SO_4_, filtered, and the solvent was evaporated under reduced pressure providing the desired compound **55** as a white solid in a 31% yield. *R*_f_ = 0.41 (DCM/EtOAc 9:1 + 1% triethylamine). Mp = 112.8 °C. ^1^H NMR (300 MHz, methanol-*d*_4_) δ 7.68 (d, *J* = 8.2 Hz, 2H), 7.60 (d, *J* = 8.2 Hz, 2H), 7.53 (d, *J* = 8.7 Hz, 2H), 7.26 (d, *J* = 16.4 Hz, 1H), 7.08 (d, *J* = 16.4 Hz, 1H), 6.95 (d, *J* = 8.7 Hz, 2H), 4.15 (t, *J* = 5.6 Hz, 2H), 3.02 (t, *J* = 5.6 Hz, 2H), 2.78 (q, *J* = 7.2 Hz, 4H), 1.14 (t, *J* = 7.2 Hz, 6H).

#### Synthesis of (*E*)-*N*,*N*-Diethyl-2-(4-(3-methoxystyryl)phenoxy)ethan-1-amine (**56**)

Obtained from (*E*)-4-(3-methoxystyryl)phenol **50** (60 mg, 0.27 mmol) K_2_CO_3_ (3 equiv), KI (0.1 equiv), and 2-chloro-*N*,*N*-diethylethylamine hydrochloride (1.2 equiv) in methyl ethyl ketone (5 mL), according to Method A. The residue was diluted with EtOAc, washed with water, and then extracted with 1 M HCl. The aqueous phase was basified with 1 M NaOH until pH 12 and then extracted with EtOAc (3 × 25 mL). The combined organic phases were dried over anhydrous Na_2_SO_4_, filtered, and the solvent was evaporated under reduced pressure providing the desired compound **56** as a yellow oil in a 23% yield. *R*_f_ = 0.31 (DCM/EtOAc 9:1 + 1% triethylamine). ^1^H NMR (300 MHz, chloroform-*d*) δ 7.44 (d, *J* = 8.7 Hz, 2H), 7.26 (t, *J* = 7.9 Hz, 1H), 7.13–6.95 (m, 4H), 6.90 (d, *J* = 8.8 Hz, 2H), 6.80 (dd, *J* = 7.9, 2.0 Hz, 1H), 4.10 (t, *J* = 6.2 Hz, 2H), 3.84 (s, 3H), 2.93 (t, *J* = 6.2 Hz, 2H), 2.69 (q, *J* = 7.1 Hz, 4H), 1.09 (t, *J* = 7.1 Hz, 6H).

#### Synthesis of (*E*)-*N*,*N*-Diethyl-2-(4-(4-methoxystyryl)phenoxy)ethan-1-amine (**57**)

Obtained from (*E*)-4-(4-methoxystyryl)phenol **51** (100 mg, 0.44 mmol, 1 equiv), K_2_CO_3_ (3 equiv), KI (0.1 equiv), and 2-chloro-*N*,*N*-diethylethylamine hydrochloride (1.2 equiv) in methyl ethyl ketone (5 mL) according to Method A. The residue was diluted with EtOAc, washed with water and then extracted with 1 M HCl. The aqueous phase was basified with 1 M NaOH until pH 12 and then extracted with EtOAc (3 × 15 mL). The combined organic phases were dried over anhydrous Na_2_SO_4_, filtered, and the solvent was evaporated under reduced pressure providing the desired compound **57** as a white solid in an 84% yield. *R*_f_ = 0.32 (DCM/EtOAc 9:1 + 1% triethylamine). Mp = 137.6 °C. ^1^H NMR (300 MHz, chloroform-*d*) δ 7.45–7.38 (m, 4H), 6.94–6.86 (m, 6H), 4.18 (t, *J* = 6.0 Hz, 2H), 3.82 (s, 3H), 3.02 (t, *J* = 6.0 Hz, 2H), 2.79 (q, *J* = 7.0 Hz, 4H), 1.17 (t, *J* = 7.0 Hz, 6H).

#### Synthesis of (*E*)-4-(3-(Trifluoromethyl)styryl)phenol (**58**)

Obtained from 3-iodobenzotrifluoride (1.0 g, 3.68 mmol, 1 equiv) and 4-vinylphenol **42** (1.2 equiv) according to Method F at reflux temperature overnight. The crude was purified by silica gel flash column chromatography (gradient from cyclohexane/EtOAc 9:1 to 7:3), providing the desired compound **58** as a pale-yellow solid in a 44% yield. *R*_f_ = 0.41 (cyclohexane/EtOAc 8:2). Mp = 119.1–123.0 °C. ^1^H NMR (300 MHz, chloroform-*d*) δ 7.75–7.69 (m, 1H), 7.67–7.60 (m, 1H), 7.51–7.39 (m, 4H), 7.11 (d, *J* = 16.3 Hz, 1H), 6.97 (d, *J* = 16.3 Hz, 1H), 6.90–6.80 (m, 2H), 4.80 (s, 1H).

#### Synthesis of (*E*)-4-(3-((2-Methoxyethoxy)methoxy)styryl)phenol (**59**)

Obtained from 1-iodo-3-((2-methoxyethoxy)methoxy)benzene **109** (870 mg, 2.82 mmol, 1 equiv) and 4-vinylphenol **42** (1.2 equiv) according to Method F. The crude was purified by silica gel flash column chromatography (gradient from cyclohexane/EtOAc 9:1 to 7:3), providing the desired product **59** as a yellow oil in a 65% yield. *R*_f_ = 0.25 (cyclohexane/EtOAc 9:1). ^1^H NMR (300 MHz, chloroform-*d*) δ 7.39 (d, *J* = 8.8 Hz, 2H), 7.30–7.17 (m, 2H), 7.18–7.09 (m, 2H), 7.04 (d, *J* = 16.5 Hz, 1H), 6.91 (d, *J* = 16.5 Hz, 1H), 6.84 (d, *J* = 8.8 Hz, 2H), 5.32 (s, 2H), 5.27 (s, 1H, exchanges with D_2_O), 3.90–3.84 (m, 2H), 3.63–3.57 (m, 2H), 3.41 (s, 3H).

#### Synthesis of (*E*)-1-(4-(2-Bromoethoxy)styryl)-3-(trifluoromethyl)benzene (**60**)

Obtained from **58** (40 mg, 0.15 mmol, 1 equiv), K_2_CO_3_ (3 equiv), KI (0.1 equiv), and 1,2-dibromoethane (30 μL, 0.315 mmol, 2.1 equiv) in methyl ethyl ketone (2 mL), according to Method A. The residue was purified by flash chromatography (cyclohexane/EtOAc gradient from 9:1 to 8:2) affording **60** as a colorless oil in an 89% yield. *R*_f_ = 0.37 (cyclohexane/EtOAc 8:2). ^1^H NMR (300 MHz, chloroform-*d*) δ 7.76–7.70 (m, 1H), 7.67–7.61 (m, 1H), 7.52–7.41 (m, 4H), 7.12 (d, *J* = 16.3 Hz, 1H), 6.99 (d, *J* = 16.3 Hz, 1H), 6.96–6.88 (m, 2H), 4.32 (t, *J* = 6.3 Hz, 2H), 3.66 (t, *J* = 6.3 Hz, 2H).

#### Synthesis of (*E*)-1-(4-(2-Bromoethoxy)styryl)-3-((2-methoxyethoxy)methoxy)benzene (**61**)

Obtained from (*E*)-4-(3-((2-methoxyethoxy)methoxy)styryl)phenol **59** (430 mg, 1.43 mmol, 1 equiv), K_2_CO_3_ (1.5 equiv), KI (0.1 equiv), and 1,2-dibromoethane (2.1 equiv) in methyl ethyl ketone (15 mL) according to Method A. The crude was purified by silica gel flash column chromatography (gradient from cyclohexane/EtOAc 9:1 to 7:3), providing the desired intermediate **61** as a yellow oil in a 26% yield. *R*_f_ = 0.39 (cyclohexane/EtOAc 8:2). ^1^H NMR (300 MHz, chloroform-*d*) δ 7.45 (d, *J* = 8.7 Hz, 2H), 7.29–7.23 (m, 1H), 7.21–7.11 (m, 2H), 7.05 (d, *J* = 16.4 Hz, 1H), 6.99–6.87 (m, 4H), 5.31 (s, 2H), 4.32 (t, *J* = 6.3 Hz, 2H), 3.89–3.79 (m, 2H), 3.65 (t, *J* = 6.3 Hz, 2H), 3.62–3.51 (m, 2H), 3.39 (s, 3H).

#### Synthesis of (*E*)-1-(4-(2-Iodoethoxy)styryl)-3-(trifluoromethyl)benzene (**62**)

Obtained from **60** (50 mg, 0.135 mmol, 1 equiv) according to Method D, providing the desired compound **62** as a colorless oil in an 83% yield. *R*_f_ = 0.40 (cyclohexane/EtOAc 8:2). ^1^H NMR (300 MHz, chloroform-*d*) δ 7.75–7.70 (m, 1H), 7.69–7.56 (m, 1H), 7.50–7.44 (m, 4H), 7.12 (d, *J* = 16.4 Hz, 1H), 6.99 (d, *J* = 16.4 Hz, 1H), 6.95–6.86 (m, 2H), 4.33–4.23 (m, 2H), 3.48–3.39 (m, 2H).

#### Synthesis of (*E*)-1-(4-(2-Iodoethoxy)styryl)-3-((2-methoxyethoxy)methoxy)benzene (**63**)

Obtained from (*E*)-1-(4-(2-bromoethoxy)styryl)-3-((2-methoxyethoxy)methoxy)benzene **61** (150 mg, 0.37 mmol, 1 equiv) according to Method D, providing the desired compound **63** as a yellow oil in a 77% yield. *R*_f_ = 0.39 (cyclohexane/EtOAc 8:2). ^1^H NMR (300 MHz, chloroform-*d*) δ 7.45 (d, *J* = 8.7 Hz, 2H), 7.29–7.26 (m, 1H), 7.21–7.09 (m, 2H), 7.05 (d, *J* = 16.2 Hz, 1H), 6.99–6.84 (m, 4H), 5.31 (s, 2H), 4.27 (dd, *J* = 7.4, 6.4 Hz, 2H), 3.87–3.82 (m, 2H), 3.61–3.55 (m, 2H), 3.43 (dd, *J* = 7.4, 6.4 Hz, 2H), 3.39 (s, 3H).

#### Synthesis of (*E*)-3-(4-(2-Iodoethoxy)styryl)phenol (**64**)

A mixture of (*E*)-3-(2-methoxyethoxymethyloxy)-4′-(2-iodoethyloxy)stilbene **63** (129 mg, 0.28 mmol, 1 equiv) and an excess of methanolic solution of 1.25 M HCl was stirred at 65 °C overnight. Upon cooling at room temperature, the resulting mixture was concentrated under reduced pressure, providing the desired product **64** as a pale-pink solid in an 82% yield. *R*_f_ = 0.29 (cyclohexane/EtOAc 8:2). Mp = 113.4 °C. ^1^H NMR (300 MHz, chloroform-*d*) δ 7.44 (d, *J* = 8.7 Hz, 2H), 7.25–7.18 (m, 1H), 7.09–6.99 (m, 2H), 6.99–6.87 (m, 4H), 6.72 (dd, *J* = 8.1, 2.4 Hz, 1H), 4.30–4.24 (m, 2H), 3.47–3.39 (m, 2H).

#### Synthesis of (*E*)-4-(4-((2-Methoxyethoxy)methoxy)styryl)phenol (**65**)

Obtained from 1-iodo-4-((2-methoxyethoxy)methoxy)benzene^23^ (750 mg, 2.43 mmol, 1 equiv) and 4-vinylphenol **42** (1.1 equiv) according to Method F. The crude was purified by silica gel flash column chromatography (cyclohexane/EtOAc gradient from 9:1 to 7:3), providing the desired product **65** as a white solid in a 65% yield. *R*_f_ = 0.4 (DCM/EtOAc 9:1). Mp = 113.4–114.1 °C. ^1^H NMR (300 MHz, chloroform-*d*) δ 7.45–7.33 (m, 4H), 7.07–6.98 (m, 2H), 6.92 (s, 2H), 6.86–6.77 (m, 2H), 5.28 (s, 2H), 4.98 (s, 1H), 3.89–3.79 (m, 2H), 3.62–3.53 (m, 2H), 3.39 (s, 3H).

#### Synthesis of (*E*)-*N*,*N*-Diethyl-2-(4-(4-((2-methoxyethoxy)methoxy)styryl)phenoxy)ethan-1-amine (**66**)

Obtained from **65** (200 mg, 0.67 mmol, 1 equiv), K_2_CO_3_ (3.0 equiv), KI (0.1 equiv), and 2-chloro-*N*,*N*-diethylethylamine hydrochloride (1.2 equiv) in methylethylketone (25 mL) according to Method A. The residue was diluted with water and extracted with EtOAc (3 × 15 mL). The organic phases were combined and extracted with 1 M HCl. The aqueous phase was basified with 1 M NaOH and extracted with EtOAc (3 × 30 mL). The organic phases were combined, dried over anhydrous Na_2_SO_4_, filtered, and the solvent was evaporated under vacuum to give **66** as a yellow oil in a 20% yield. *R*_f_ = 0.2 (DCM/EtOAc 9:1 + 1% triethylamine). ^1^H NMR (300 MHz, chloroform-*d*) δ 7.43 (d, *J* = 8.8 Hz, 2H), 7.42 (d, *J* = 8.8 Hz, 2H), 7.04 (d, *J* = 8.8 Hz, 2H), 6.93 (s, 2H), 6.88 (d, *J* = 8.8 Hz, 2H), 5.29 (s, 2H), 4.58–4.51 (m, 2H), 3.86–3.81 (m, 2H), 3.59–3.54 (m, 2H), 3.51–3.41 (m, 2H), 3.38 (s, 3H), 3.26 (q, *J* = 7.4 Hz, 4H), 1.47 (t, *J* = 7.4 Hz, 6H).

#### Synthesis of (*E*)-*N*,*N*,*N*-Triethyl-2-(4-(4-((2-methoxyethoxy)methoxy)styryl)phenoxy)ethan-1-aminium Iodide (**67**)

Obtained from **66** (50 mg, 0.13 mmol) and iodoethane (9.6 equiv) in THF (2 mL) according to Method B at reflux for 16 h. Upon cooling to room temperature, the reaction mixture was diluted with diethyl ether and the resulting suspension was filtered, affording **67** as a light-yellow solid in a 46% yield. Mp = 130.4–130.8 °C. ^1^H NMR (300 MHz, methanol-*d*_4_): δ 7.51 (d, *J* = 8.8 Hz, 2H), 7.46 (d, *J* = 8.8 Hz, 2H), 7.05–6.96 (m, 6H), 5.27 (s, 2H), 4.50–4.39 (m, 2H), 3.83–3.78 (m, 2H), 3.81–3.72 (m, 2H), 3.58–3.54 (m, 2H), 3.48 (q, *J* = 7.2 Hz, 6H), 3.33 (s, 3H), 1.37 (t, *J* = 7.2 Hz, 9H).

#### Synthesis of (*E*)-4-(2-(Naphthalen-1-yl)vinyl)phenol (**68**)

1-Naphthylmethyltriphenylphosphonium chloride (28.5 g, 64.9 mmol) was added to a solution of sodium (2.85 g, 124 mmol) in EtOH (100 mL) at *T* < 10 °C. A solution of 4-hydroxybenzaldehyde (7.93 g, 64.9 mmol) in EtOH (50 mL) was added to the previous mixture, and the reaction mixture was stirred at room temperature for 48 h. The mixture was concentrated under reduced pressure. The residue was taken in diethyl ether and 3 M HCl, and the phases were separated. The organic phase was washed with water, dried over anhydrous Na_2_SO_4_, filtered, and evaporated affording the crude product. Flash chromatography (cyclohexane/EtOAc from 90:10 to 85:15—second eluted isomer) and subsequent crystallization from cyclohexane/CHCl_3_ afforded compound **68** in a 53% yield. Mp = 135–138 °C. *R*_f_ = 0.3 (cyclohexane/EtOAc 8:2). ^1^H NMR (200 MHz, chloroform-*d*) δ 8.28–8.20 (m, 1H), 7.95–7.70 (m, 4H), 7.65–7.45 (m, 5H), 7.11 (d, *J* = 16.0 Hz, 1H), 7.00–6.80 (m, 2H), 5.15 (s, 1H).

#### Synthesis of (*E*)-*N*,*N*-Diethyl-2-(4-(2-(naphthalen-1-yl)vinyl)phenoxy)ethan-1-amine (**69**)

Obtained from **68** (2.00 g, 8.12 mmol), 2-chloro-*N*,*N*-diethylethylamine hydrochloride (1.2 equiv), and K_2_CO_3_ (2.24 g, 16.2 mmol) in acetone (30 mL) according to Method A at reflux for 4 h. Upon filtration, the filtrate was taken in diethyl ether and 2 M NaOH. The organic phase was separated and washed again with 2 M NaOH, water, dried over anhydrous Na_2_SO_4_, filtered, and concentrated under vacuum to give **69** as a yellow oil in an 80% yield. *R*_f_ = 0.8 (DCM/MeOH 98:2 + 1.0% NH_3(aq20%)_). ^1^H NMR (200 MHz, chloroform-*d*) δ 8.30–8.20 (m, 1H), 7.90–7.70 (m, 4H), 7.60–7.40 (m, 5H), 7.11 (d, *J* = 16.0 Hz, 1H), 7.06–6.86 (m, 2H), 4.19–3.99 (m, 2H), 2.99–2.88 (m, 2H), 2.80–2.55 (m, 4H), 1.22–0.99 (m, 6H).

#### Synthesis of (*E*)-4-(2-(Naphthalen-2-yl)vinyl)phenol (**70**)

2-Naphthylmethyltriphenylphosphonium bromide (36.77 g, 76 mmol) was added to a solution of sodium (3.49 g, 152 mmol) in EtOH (100 mL) at *T* < 10 °C. A solution of 4-hydroxybenzaldehyde (9.28 g, 76 mmol) in EtOH (50 mL) was added to the previous mixture, and the reaction mixture was stirred at room temperature for 72 h. The mixture was concentrated under reduced pressure. The residue was taken in diethyl ether and 3 M HCl, and the phases were separated. The organic phase was washed with water, dried over anhydrous Na_2_SO_4_, filtered, and evaporated affording the crude product. The crude product was suspended in diethyl ether, and the mixture was vigorously stirred for 30 min. The solid was isolated by filtration and purified by flash chromatography (cyclohexane/EtOAc 8:2) to give compound **70** in a 31% yield. Mp = 208–211 °C (coherent with the literature^20^). *R*_f_ = 0.55 (cyclohexane/EtOAc 1:1). ^1^H NMR (200 MHz, dimethylsulfoxide-*d*_6_) δ 9.68 (s, 1H), 8.05–7.80 (m, 5H), 7.60–7.45 (m, 4H), 7.37 (d, *J* = 16.0 Hz, 1H), 7.22 (d, *J* = 16.0 Hz, 1H), 6.95–6.74 (m, 2H).

#### Synthesis of (*E*)-*N*,*N*-Diethyl-2-(4-(2-(naphthalen-2-yl)vinyl)phenoxy)ethan-1-amine (**71**)

Obtained from **70** (2.00 g, 8.12 mmol), 2-chloro-*N*,*N*-diethylethylamine hydrochloride (1.2 equiv), and K_2_CO_3_ (2.24 g, 16.2 mmol) in acetone (30 mL) according to Method A at reflux for 4 h. Upon filtration, the residue was purified by flash column chromatography (DCM/MeOH 95:5) to give **71** as a yellow oil in a 53% yield. *R*_f_ = 0.75 (DCM/MeOH 98:2 + 1.0% NH_3(aq20%)_). ^1^H NMR (200 MHz, chloroform-*d*) δ 7.90–7.70 (m, 5H), 7.55–7.40 (m, 4H), 7.22 (d, *J* = 16.0 Hz, 1H), 7.12 (d, *J* = 16.0 Hz, 1H), 7.02–6.84 (m, 2H), 4.18–4.00 (m, 2H), 2.99–2.79 (m, 2H), 2.80–2.52 (m, 4H), 1.18–1.00 (m, 6H).

#### Synthesis of 4-Phenethylphenol (**72**)

Pd/C (0.60 g) was added to a solution of 4-hydroxystilbene (1.00 g, 5.09 mmol) in MeOH (50 mL), and the reaction mixture was stirred under a hydrogen atmosphere for 24 h. The reaction mixture was filtered through a short layer of celite, and the volatiles were evaporated under vacuum to give **72** as a white solid (1.00 g, 100%). Mp = 97.2–98.2 °C (coherent with the literature^24^). *R*_f_ = 0.5 (cyclohexane/EtOAc 7:3). ^1^H NMR (300 MHz, chloroform-*d*) δ 7.36–7.11 (m, 5H), 7.09–6.98 (m, 2H), 6.80–6.68 (m, 2H), 2.98–2.74 (m, 4H).

#### Synthesis of *N*,*N*-Diethyl-2-(4-phenethylphenoxy)ethan-1-amine (**73**)

Obtained from compound **72** (0.5 g, 2.52 mmol, 1 equiv), K_2_CO_3_ (4.0 equiv), KI (0.1 equiv), and 2-chloro-*N*,*N*-diethylethylamine hydrochloride (1.2 equiv) in methyl ethyl ketone (20 mL), according to Method A, at reflux temperature for 24 h. The residue was taken in 1 M HCl (100 mL) and washed with diethyl ether (50 mL). The water phase was basified with 3 M NaOH and extracted with EtOAc (3 × 50 mL). The combined organic phase was washed with brine, dried over anhydrous Na_2_SO_4_, filtered, and evaporated under reduced pressure. Purification by silica gel flash column chromatography (DCM/MeOH 95:5 + 0.5% NH_3(aq20%)_) afforded **73** as a colorless oil (0.62 g, 82%). *R*_f_ = 0.4 (DCM/MeOH 95:5 + 0.5% NH_3(aq20%)_). ^1^H NMR (300 MHz, chloroform-*d*) δ 7.32–7.24 (m, 2H), 7.23–7.14 (m, 3H), 7.11–7.04 (m, 2H), 6.86–6.78 (m, 2H), 4.22–4.04 (m, 2H), 3.07–2.93 (m, 2H), 2.92–2.84 (m, 4H), 2.84–2.67 (m, 4H), 1.39–0.92 (m, 6H).

#### Synthesis of 1-(2-Bromoethoxy)-4-(phenylethynyl)benzene (**74**)

Obtained from 4-(phenylethynyl)phenol (280 mg, 1.44 mmol, 1 equiv), K_2_CO_3_ (2.5 equiv), KI (0.1 equiv), and 1,2-dibromoethane (4.2 equiv) in methyl ethyl ketone (5 mL) according to Method A. The crude was purified by silica gel flash column chromatography (gradient from cyclohexane to cyclohexane/EtOAc 9:1), providing the desired product **74** as a white solid in a 66% yield. *R*_f_ = 0.56 (cyclohexane/EtOAc 9:1). Mp = 88.7 °C. ^1^H NMR (300 MHz, chloroform-*d*) δ 7.54–7.44 (m, 4H), 7.36–7.29 (m, 3H), 6.89 (d, *J* = 8.9 Hz, 2H), 4.31 (t, *J* = 6.3 Hz, 2H), 3.65 (t, *J* = 6.3 Hz, 2H).

#### Synthesis of 1-(2-Iodoethoxy)-4-(phenylethynyl)benzene (**75**)

Obtained from 1-(2-bromoethoxy)-4-(phenylethynyl)benzene **74** (118 mg, 0.39 mmol, 1 equiv) according to Method D, providing the desired compound **75** as a white solid in a 92% yield. *R*_f_ = 0.67 (cyclohexane/EtOAc 9:1). Mp = 108.3 °C. ^1^H NMR (300 MHz, chloroform-*d*) δ 7.56–7.41 (m, 4H), 7.39–7.28 (m, 3H), 6.88 (d, *J* = 9.0 Hz, 2H), 4.33–4.21 (m, 2H), 3.50–3.35 (m, 2H).

#### Synthesis of (*E*)-5-(4-(2-Chloroethoxy)styryl)benzene-1,3-diol (**76**)

Obtained from resveratrol (446 mg, 1.95 mmol, 1 equiv), K_2_CO_3_ (1.1 equiv), and 1-bromo-2-chloroethane (1.5 equiv) in DMF (2 mL) according to Method A, at 60 °C, overnight. The reaction mixture was concentrated under reduced pressure, diluted with EtOAc, and washed with 1 M HCl. The organic phase was dried over anhydrous Na_2_SO_4_, filtered, and the solvent was evaporated under reduced pressure. The crude was purified by silica gel flash chromatography providing the desired compound **76** as a white solid in a 40% yield. *R*_f_ = 0.2 (DCM/EtOAc). Mp = 161 °C (coherent with the literature^5^). ^1^H NMR (300 MHz, dimethylsulfoxide-*d*_6_) δ 9.17 (s, 2H, exchanges with D_2_O), 7.49 (d, *J* = 8.8 Hz, 2H), 7.04–6.81 (m, 4H), 6.37 (d, *J* = 2.1 Hz, 2H), 6.10 (t, *J* = 2.1 Hz, 1H), 4.28–4.20 (m, 2H), 3.95–3.87 (m, 2H).

#### Synthesis of (*E*)-5-(4-(2-Iodoethoxy)styryl)benzene-1,3-diol (**77**)

Obtained from (*E*)-5-(4-(2-chloroethoxy)styryl)benzene-1,3-diol **76** (230 mg, 0.79 mmol, 1 equiv), according to Method D, providing the desired compound **77** as a pale-yellow solid in a 97% yield. *R*_f_ = 0.2 (DCM/EtOAc). Mp = 138 °C. ^1^H NMR (300 MHz, dimethylsulfoxide-*d*_6_) δ 9.23 (s, 2H, exchanges with D_2_O), 7.51 (d, *J* = 8.2 Hz, 2H), 6.98–6.89 (m, 4H), 6.39 (d, *J* = 2.2 Hz, 2H), 6.12 (t, *J* = 2.2 Hz, 1H), 4.27 (t, *J* = 6.3 Hz, 2H), 3.52 (t, *J* = 6.3 Hz, 2H).

#### Synthesis of 4-(Naphthalen-2-yl)phenol (**78**)

Obtained from 2-bromonapthalene (273 mg, 1.24 mmol, 1 equiv), *p*-hydroxyphenyl boronic acid (1.1 equiv), Pd(PPh_3_)_4_ (0.35 equiv), and TBAB (0.05 equiv) in 1,2-dimethoxyethane, according to Method C. The crude was purified by silica gel flash column chromatography (toluene/EtOAc 95:5), providing the desired compound **78** as a white solid in a 92% yield. *R*_f_ = 0.48 (cyclohexane/EtOAc 9:1). Mp = 167 °C (coherent with the literature^25^). ^1^H NMR (300 MHz, chloroform-*d*) δ 8.01–7.96 (m, 1H), 7.92–7.82 (m, 3H), 7.71 (dd, *J* = 8.6, 1.9 Hz, 1H), 7.62 (d, *J* = 8.7 Hz, 2H), 7.53–7.43 (m, 2H), 6.96 (d, *J* = 8.7 Hz, 2H), 4.86 (s, 1H).

#### Synthesis of *N*,*N*-Diethyl-2-(4-(naphthalen-2-yl)phenoxy)ethan-1-amine (**79**)

Obtained from 4-(naphthalen-2-yl)phenol **78** (210 mg, 0.95 mmol, 1 equiv), K_2_CO_3_ (2.5 equiv), KI (0.1 equiv), and 2-chloro-*N*,*N*-diethylethylamine hydrochloride (1.2 equiv) in methyl ethyl ketone (10 mL) according to Method A. The crude was diluted with diethyl ether and washed three times with a 1 M NaOH solution. The organic phase was dried over anhydrous Na_2_SO_4_ and filtered. The filtrate was evaporated under vacuum, providing the desired product **79** as a white solid in a 27% yield. *R*_f_ = 0.29 (DCM/MeOH 95:5 + 0.5% NH_3(20% aq)_). Mp = 51 °C. ^1^H NMR (300 MHz, chloroform-*d*) δ 8.01–7.97 (m, 1H), 7.92–7.83 (m, 3H), 7.72 (dd, *J* = 8.5, 1.9 Hz, 1H), 7.66 (d, *J* = 8.9 Hz, 2H), 7.55–7.42 (m, 2H), 7.03 (d, *J* = 8.9 Hz, 2H), 4.12 (t, *J* = 6.4 Hz, 2H), 2.93 (t, *J* = 6.4 Hz, 2H), 2.68 (q, *J* = 7.1 Hz, 4H), 1.10 (t, *J* = 7.1 Hz, 6H).

#### Synthesis of 4-(Naphthalen-1-yl)phenol (**80**)

Obtained from 1-bromonapthalene (158 mg, 0.76 mmol, 1 equiv), p-hydroxyphenyl boronic acid (1.1 equiv), Pd(PPh_3_)_4_ (0.35 equiv), and TBAB (0.05 equiv) in 1,2-dimethoxyethane according to Method C. The crude was purified by silica gel flash column chromatography (toluene/EtOAc 95:5), providing the desired product **80** as a white solid in a 50% yield. *R*_f_ = 0.48 (cyclohexane/EtOAc 7:3). Mp = 91 °C (coherent with the literature^26^). ^1^H NMR (300 MHz, chloroform-*d*) δ 8.00–7.87 (m, 2H), 7.86 (d, *J* = 8.2 Hz, 1H), 7.58–7.34 (m, 6H), 6.99 (d, *J* = 8.8 Hz, 2H), 5.22 (s, 1H, exchanges with D_2_O).

#### Synthesis of *N*,*N*-Diethyl-2-(4-(naphthalen-1-yl)phenoxy)ethan-1-amine (**81**)

Obtained from 4-(naphthalen-1-yl)phenol **80** (84 mg, 0.38 mmol, 1 equiv), K_2_CO_3_ (4 equiv), KI (0.1 equiv), and 2-chloro-*N*,*N*-diethylethylamine hydrochloride (1.2 equiv) in methyl ethyl ketone (5 mL), according to Method A, at reflux temperature, overnight. The residue was diluted in diethyl ether and washed with water. The organic phase was dried over anhydrous Na_2_SO_4_, filtered, and the solvent was evaporated under reduced pressure. The crude was purified by silica gel flash column chromatography (cyclohexane/EtOAc 7:3 + 0.5% triethylamine), providing the desired compound **81** as a pale-yellow oil in a 46% yield. *R*_f_ = 0.20 (cyclohexane/EtOAc 7:3 + 0.5% triethylamine). ^1^H NMR (300 MHz, chloroform-*d*) δ 7.95–7.78 (m, 3H), 7.54–7.36 (m, 6H), 7.03 (d, *J* = 8.6 Hz, 2H), 4.16 (t, *J* = 6.5 Hz, 2H), 2.96 (t, *J* = 6.5 Hz, 2H), 2.70 (q, *J* = 7.0 Hz, 4H), 1.12 (t, *J* = 7.0 Hz, 6H).

#### Synthesis of 4-Acetoxybenzoic Acid (**82**)

A solution of 4-hydroxybenzoic acid (1.00 g, 7.24 mmol, 1 equiv) in acetic anhydride (4 mL, 36.28 mmol, 5 equiv) and 96% of sulfuric acid (0.1 mL) was heated at 80 °C for 2 h. After cooling to 0 °C, the mixture was slowly diluted with water and precipitation of a white solid occurred. The suspension was filtered, and the solid was washed with water three times, affording the desired compound **82** as a white solid in a 97% yield. *R*_f_ = 0.47 (cyclohexane/EtOAc 9:1). Mp = 199–201 °C (coherent with the literature^27^). ^1^H NMR (300 MHz, chloroform-*d*) δ 8.15 (d, *J* = 7.6 Hz, 2H), 7.32–7.10 (m, 2H), 2.47–2.15 (s, 3H).

#### Synthesis of 4-(Phenylcarbamoyl)phenyl Acetate (**83**)

The reaction was performed under inert conditions. A catalytic amount of DMF (5 drops) was added dropwise to a suspension of 4-acetoxybenzoic acid **82** (350 mg, 1.94 mmol, 1 equiv) in DCM (15 mL). The mixture was cooled to 0 °C, and oxalyl chloride (493 mg, 3.88 mmol, 2 equiv) was added dropwise. The reaction mixture was stirred at room temperature for 2 h and then concentrated under reduced pressure to obtain a crude containing the activated acyl chloride, which was directly dissolved in DCM (10 mL) under a nitrogen atmosphere. The mixture was cooled to 0 °C, and a solution of aniline (542 mg, 5.82 mmol, 3 equiv) in DCM (3 mL) was added dropwise to the mixture. The resulting suspension was stirred for 1 h at room temperature and then washed with a 1 M HCl aqueous solution. The organic layer was dried over anhydrous Na_2_SO_4_, filtered, and the solvent was evaporated under reduced pressure. The crude was purified through silica gel flash column chromatography (toluene/EtOAc 9:1), affording the desired compound **83** as a white solid in a 96% yield. *R*_f_ = 0.67 (toluene/EtOAc 1:1). Mp = 168 °C (coherent with the literature^28^). ^1^H NMR (300 MHz, dimethylsulfoxide-*d*_6_) δ 10.25 (s, 1H), 8.00 (d, *J* = 8.7 Hz, 2H), 7.76 (d, *J* = 8.3 Hz, 2H), 7.41–7.24 (m, 4H), 7.10 (t, *J* = 7.5 Hz, 1H), 2.31 (s, 3H).

#### Synthesis of 4-Hydroxy-*N*-phenylbenzamide (**84**)

An aqueous solution of 1 M NaOH (3 mL) was added to a suspension of 4-(phenylcarbamoyl)phenyl acetate **83** (520 mg, 2.04 mmol, 1 equiv) in MeOH (5 mL). The resulting solution was stirred at room temperature for 30 min. The reaction mixture was diluted with water (5 mL), the pH was adjusted to 6 by the dropwise addition of 1 M HCl, and the product was extracted with EtOAc. The organic phase was dried over anhydrous Na_2_SO_4_, filtered, and the solvent was evaporated under reduced pressure, affording the desired compound **84** as a white powder in a 100% yield. *R*_f_ = 0.47 (cyclohexane/EtOAc 1:1). Mp = 203 °C (coherent with the literature^29^). ^1^H NMR (300 MHz, dimethylsulsoxide-*d*_6_) δ 10.07 (s, 1H), 9.96 (s, 1H), 7.85 (d, *J* = 8.7 Hz, 2H), 7.75 (dd, *J* = 8.6, 1.2 Hz, 2H), 7.33 (t, *J* = 8.6 Hz, 2H), 7.06 (tt, *J* = 7.2, 1.2 Hz, 1H), 6.86 (d, *J* = 8.7 Hz, 2H).

#### Synthesis of 4-(2-Chloroethoxy)-*N*-phenylbenzamide (**85**)

A suspension of 4-hydroxy-*N*-phenylbenzamide **84** (420 mg, 1.97 mmol, 1 equiv) and Cs_2_CO_3_ (1.2 equiv) in DMF (10 mL) was stirred at room temperature for 30 min. 1-Chloro-2-bromoethane (1.5 equiv) was added dropwise, and the resulting mixture was stirred at 60 °C overnight. The suspension was diluted with diethyl ether, washed with 1 M NaOH, and the organic phase was dried over anhydrous Na_2_SO_4_, filtered, and the solvent was evaporated under reduced pressure, providing the desired compound **85** as a pale-yellow powder in a 39% yield. *R*_f_ = 0.30 (cyclohexane/EtOAc 1:1). Mp = 174 °C. ^1^H NMR (300 MHz, chloroform-*d*) δ 7.85 (d, *J* = 8.7 Hz, 2H), 7.74 (s, 1H), 7.63 (d, *J* = 7.6 Hz, 2H), 7.37 (t, *J* = 7.6 Hz, 2H), 7.14 (t, *J* = 7.6 Hz, 1H), 7.00 (d, *J* = 8.7 Hz, 2H), 4.30 (t, *J* = 5.8 Hz, 2H), 3.85 (t, *J* = 5.8 Hz, 2H).

#### Synthesis of 4-(2-Iodoethoxy)-*N*-phenylbenzamide (**86**)

Obtained according to Method D from 4-(2-chloroethoxy)-*N*-phenylbenzamide **85** (220 mg, 0.80 mmol, 1 equiv), providing the desired intermediate **86** as a white powder in an 84% yield. *R*_f_ = 0.35 (cyclohexane/EtOAc 1:1). Mp = 172 °C. ^1^H NMR (300 MHz, chloroform-*d*) δ 7.85 (d, *J* = 8.7 Hz, 2H), 7.71 (s, 1H), 7.62 (d, *J* = 8.4 Hz, 2H), 7.37 (m, 2H), 7.14 (t, *J* = 7.0 Hz, 1H), 6.98 (d, *J* = 8.7 Hz, 2H), 4.32 (t, *J* = 6.7 Hz, 2H), 3.45 (t, *J* = 6.7 Hz, 2H).

#### Synthesis of 4-(2-(Diethylamino)ethoxy)-*N*-phenylbenzamide (**87**)

Obtained from 4-(2-iodoethoxy)-*N*-phenylbenzamide **86** (100 mg, 0.27 mmol, 1 equiv), according to Method E, using diethylamine as a solvent (3 mL), at reflux temperature for 3 h. The reaction mixture was concentrated under reduced pressure, and the residue was diluted in diethyl ether. The organic phase was extracted with 1 M HCl, the pH of the aqueous phase was adjusted to 9 with NH_3(aq 30%)_, and the product was re-extracted with EtOAc. The organic phase was dried over anhydrous Na_2_SO_4_, filtered, and the solvent was evaporated under reduced pressure, providing the desired compound **87** as a pale brown oil in a 100% yield. *R*_f_ = 0.60 (DCM/MeOH 9:1 + 0.5% NH_3(aq30%)_). ^1^H NMR (300 MHz, chloroform-*d*) δ 7.84 (d, *J* = 8.8 Hz, 2H), 7.77 (s, 1H), 7.63 (dd, *J* = 7.5, 1.2 Hz, 2H), 7.42–7.29 (m, 2H), 7.14 (tt, *J* = 7.1, 1.2 Hz, 1H), 6.98 (d, *J* = 8.8 Hz, 2H), 4.19 (t, *J* = 6.0 Hz, 2H), 2.99 (t, *J* = 6.0 Hz, 2H), 2.75 (q, *J* = 7.1 Hz, 4H), 1.13 (t, *J* = 7.1 Hz, 6H).

#### Synthesis of *N*-(4-Hydroxyphenyl)benzamide (**88**)

A suspension of 4-aminophenol (546 mg, 5 mmol, 1 equiv) and sodium octyl sulfate (0.02 equiv) was warmed under stirring in water (20 mL) until a clear solution was obtained. Afterward, a solution of benzoic anhydride (1 equiv) in CH_3_CN (5 mL) was added dropwise and the reaction mixture was stirred at room temperature for 15 min. The solution was concentrated under vacuum, and the resulting brown suspension was filtered. The crude was triturated in chloroform and filtered, providing the desired compound **88** as an off-white solid in a 99% yield. *R*_f_ = 0.47 (cyclohexane/EtOAc 1:1). Mp = 216 °C (coherent with the literature^30^). ^1^H NMR (300 MHz, dimethylsulfoxide-*d*_6_) δ 9.98 (s, exchanges with D_2_O, 1H), 9.21 (s, NH, 1H), 7.91 (d, *J* = 8.8 Hz, 2H), 7.61–7.41 (m, 5H), 6.72 (d, *J* = 8.8 Hz, 2H).

#### Synthesis of *N*-(4-(2-Chloroethoxy)phenyl)benzamide (**89**)

A suspension of *N*-(4-hydroxyphenyl)benzamide **88** (860 mg, 4 mmol, 1 equiv) and Cs_2_CO_3_ (1.2 equiv) in DMF (20 mL) was stirred at room temperature for 30 min. 1-Chloro-2-bromoethane (1.5 equiv) was added dropwise, and the resulting mixture was stirred at 60 °C overnight. The suspension was diluted with diethyl ether, washed with 1 M NaOH, and the organic phase was dried over anhydrous Na_2_SO_4_, filtered, and the solvent was evaporated under reduced pressure, providing the desired compound **89** as a pale-yellow solid in a 19% yield. *R*_f_ = 0.30 (cyclohexane/EtOAc 7:3). Mp = 177 °C. ^1^H NMR (300 MHz, chloroform-*d*) δ 7.92–7.79 (m, 2H), 7.71 (s, 1H), 7.59–7.45 (m, 5H), 6.94 (d, *J* = 9.0 Hz, 2H), 4.24 (t, *J* = 5.9 Hz, 2H), 3.82 (t, *J* = 5.9 Hz, 2H).

#### Synthesis of *N*-(4-(2-Iodoethoxy)phenyl)benzamide (**90**)

Obtained according to Method D from *N*-(4-(2-chloroethoxy)phenyl)benzamide **89** (230 mg, 0.83 mmol, 1 equiv), providing the desired compound **90** as a white powder in a 72% yield. *R*_f_ = 0.35 (cyclohexane/EtOAc 7:3). Mp = 173 °C. ^1^H NMR (300 MHz, chloroform-*d*) δ 7.86 (d, *J* = 8.3 Hz, 2H), 7.70 (s, 1H), 7.60–7.43 (m, 5H), 6.92 (d, *J* = 8.3 Hz, 2H), 4.26 (t, *J* = 7.3 Hz, 2H), 3.42 (t, *J* = 7.3 Hz, 2H).

#### Synthesis of *N*-(4-(2-(Diethylamino)ethoxy)phenyl)benzamide (**91**)

Obtained from *N*-(4-(2-iodoethoxy)phenyl)benzamide **90** (100 mg, 0.27 mmol, 1 equiv), according to Method E, using diethylamine as a solvent (3 mL), at reflux temperature for 3 h. The reaction mixture was concentrated under reduced pressure, and the residue was diluted in diethyl ether. The organic phase was extracted with 1 M HCl, the pH of the aqueous phase was adjusted to 9 with NH_3(aq30%)_, and the product was extracted again with EtOAc. The organic phase was dried over anhydrous Na_2_SO_4_, filtered, and the solvent was evaporated under reduced pressure, providing the desired compound **91** as a pale brown oil in a 100% yield. *R*_f_ = 0.60 (DCM/MeOH 9:1 + 0.5% NH_3(30%aq)_). ^1^H NMR (300 MHz, chloroform-*d*) δ 7.86 (d, *J* = 8.9 Hz, 2H), 7.71 (s, 1H), 7.57–7.44 (m, 5H), 6.92 (d, *J* = 8.9 Hz, 2H), 4.11 (t, *J* = 6.1 Hz, 2H), 2.94 (t, *J* = 6.1 Hz, 2H), 2.71 (q, *J* = 7.1 Hz, 4H), 1.12 (t, *J* = 7.1 Hz, 6H).

#### Synthesis of (*E*)-4-(Phenyldiazenyl)phenol (**92**)

Aniline (490 μL, 5.37 mmol) was dissolved in H_2_O (2.5 mL) and 37% HCl (1.3 mL, 16.11 mmol) at 0 °C. A solution of NaNO_2_ (370 mg, 5.37 mmol) in H_2_O (2.5 mL) was added dropwise at the same temperature and stirred for 15 min. A solution of phenol (505 mg, 5.37 mmol) in EtOH (2 mL) was added, and the resulting mixture was stirred for 1 h at room temperature. A saturated solution of NaHCO_3_ was added up to pH 7, and stirring was continued for 30 min. The formed solid was isolated by vacuum filtration, washed with water, and dried to give **92** as a brown solid in a 92% yield. Mp = 149 °C dec (coherent with the literature^31^). *R*_f_ = 0.65 (cyclohexane/EtOAc 7:3). ^1^H NMR (300 MHz, chloroform-*d*) δ 7.92–7.83 (m, 4H), 7.56–7.40 (m, 3H), 7.00–6.91 (m, 2H).

#### Synthesis of (*E*)-*N*,*N*-Diethyl-2-(4-(phenyldiazenyl)phenoxy)ethan-1-amine (**93**)

Obtained from **92** (0.84 g, 4.24 mmol), K_2_CO_3_ (4 equiv), KI (0.1 equiv), and 2-chloro-*N*,*N*-diethylethylamine hydrochloride (1.2 equiv) in methyl ethyl ketone (30 mL), according to Method A, at reflux temperature, overnight. The residue was obtained by filtration, and evaporation was taken in 1 M HCl (30 mL) and washed with diethyl ether (2 × 20 mL). NaOH (2 M) was added to the water phase up to pH 13, and then extraction with EtOAc (3 × 30 mL) was performed. The combined organic layer was washed with brine (30 mL), dried over anhydrous Na_2_SO_4_, filtered, and evaporated. Purification by silica gel flash chromatography (DCM/MeOH gradient from 0 to 30% MeOH) afforded **93** as a dark red oil in a 68% yield. *R*_f_ = 0.80 (DCM/MeOH + 1% NH_3(30%aq)_). ^1^H NMR (300 MHz, chloroform-*d*) δ 7.98–7.82 (m, 4H), 7.55–7.39 (m, 3H), 7.06–6.97 (m, 2H), 4.16 (t, *J* = 6.2 Hz, 2H), 2.94 (t, *J* = 6.2 Hz, 2H), 2.69 (q, *J* = 7.2 Hz, 4H), 1.11 (t, *J* = 7.2 Hz, 6H).

#### Synthesis of 4-(1*H*-Indol-6-yl)phenol (**94**)

Obtained from 6-bromoindole (300 mg, 0.15 mmol, 1 equiv), *p-*hydroxyphenyl boronic acid (2 equiv), and Pd(PPh_3_)_4_ (0.35 equiv) in EtOH/toluene 1:1 (5 mL) according to Method C, at reflux temperature overnight. The crude was purified by silica gel flash column chromatography (cyclohexane/EtOAc 7:3), followed by recrystallization from diisopropyl ether, providing the desired product **94** as a pink solid in a 42% yield. *R*_f_ = 0.26 (cyclohexane/EtOAc 7:3). Mp = 94 °C. ^1^H NMR (300 MHz, chloroform-*d*) δ 8.20 (s, 1H), 7.67 (d, *J* = 8.1 Hz, 1H), 7.57–7.49 (m, 3H), 7.33 (dd, *J* = 8.2, 1.7 Hz, 1H), 7.27–7.19 (m, 1H), 6.91 (d, *J* = 8.6 Hz, 2H), 6.57 (ddd, *J* = 3.2, 2.0, 1.0 Hz, 1H), 4.76 (s, 1H).

#### Synthesis of 2-(4-(1*H*-Indol-6-yl)phenoxy)-*N*,*N*-diethylethan-1-amine (**95**)

Obtained from 4-(1*H*-indol-6-yl)phenol **94** (52 mg, 0.25 mmol, 1 equiv), K_2_CO_3_ (4 equiv), KI (0.1 equiv), and 2-chloro-*N*,*N*-diethylethylamine hydrochloride (1.2 equiv) in methyl ethyl ketone (5 mL), according to METHOD A, at reflux temperature, overnight. The residue was diluted with EtOAc, washed with water, and then extracted with 1 M HCl. The pH of the water phase was adjusted to 9 with 1 M NaOH and further extracted with EtOAc (3 × 10 mL). The organic layer was dried over anhydrous Na_2_SO_4_, filtered, and the solvent was evaporated under reduced pressure, providing the desired compound **95** as a red oil in a 25% yield. ^1^H NMR (300 MHz, chloroform-*d*) δ 8.30 (s, 1H), 7.67 (dt, *J* = 8.2, 0.8 Hz, 1H), 7.60–7.49 (m, 3H), 7.34 (dd, *J* = 8.2, 1.6 Hz, 1H), 7.22 (dd, *J* = 3.1, 2.4 Hz, 1H), 6.98 (d, *J* = 8.8 Hz, 2H), 6.56 (ddd, *J* = 3.1, 2.0, 0.8 Hz, 1H), 4.14 (t, *J* = 6.2 Hz, 2H), 2.96 (t, *J* = 6.2 Hz, 2H), 2.72 (q, *J* = 7.2 Hz, 4H), 1.12 (t, *J* = 7.2 Hz, 6H).

#### Synthesis of 4-(1-Tosyl-1*H*-indol-5-yl)phenol (**96**)

Obtained from 5-bromo-1-tosyl-1*H*-indole (316 mg, 0.90 mmol, 1 equiv), *p-*hydroxyphenyl boronic acid (2 equiv), Pd(PPh_3_)_4_ (0.08 equiv), and TBAB in EtOH/toluene 1:3 (5 mL) according to Method C, at reflux temperature for 5 h. The crude was purified by silica gel flash column chromatography (cyclohexane/EtOAc 8:2), affording **96** as a pale-yellow oil in a 69% yield. *R*_f_ = 0.4 (cyclohexane/EtOAc 7:3). ^1^H NMR (300 MHz, chloroform-*d*) δ 8.01 (dt, *J* = 8.6, 0.8 Hz, 1H), 7.83–7.74 (m, 2H), 7.65 (dd, *J* = 1.9, 0.7 Hz, 1H), 7.57 (d, *J* = 3.7 Hz, 1H), 7.53–7.37 (m, 3H), 7.25–7.19 (m, 2H), 6.94–6.84 (m, 2H), 6.68 (dd, *J* = 3.7, 0.8 Hz, 1H), 5.09 (broad s, 1H), 2.34 (s, 3H).

#### Synthesis of 4-(1*H*-Indol-5-yl)phenol (**97**)

To a solution of **96** (135 mg, 0.317 mmol) in MeOH (15 mL), KOH (108 mg, 1.92 mmol) was added. The resulting mixture was stirred at reflux for 3 h. KOH (190 mg, 3.39 mmol) was added, and stirring was continued at the same temperature for 3 h. The solvent was evaporated, and the residue was taken in EtOAc and washed with 1 M HCl. The organic phase was washed with 1 M NaHCO_3_, brine, dried over anhydrous Na_2_SO_4_, filtered, and the solvent was concentrated under vacuum. Purification by silica gel flash chromatography (cyclohexane/EtOAc gradient from 8:2 to 1:1) provided **97** as an orange oil in a 90% yield. *R*_f_ = 0.2 (cyclohexane/EtOAc 7:3). ^1^H NMR (300 MHz, chloroform-*d*) δ 8.17 (s, 1H), 7.80 (dt, *J* = 1.6, 0.9 Hz, 1H), 7.57–7.49 (m, 2H), 7.47–7.35 (m, 2H), 7.23 (dd, *J* = 3.2, 2.4 Hz, 1H), 6.95–6.88 (m, 2H), 6.60 (ddd, *J* = 3.1, 2.0, 0.8 Hz, 1H), 4.99 (s, 1H).

#### Synthesis of 2-(4-(1*H*-Indol-5-yl)phenoxy)-*N*,*N*-diethylethan-1-amine (**98**)

Obtained from **97** (128 mg, 0.25 mmol, 1 equiv), K_2_CO_3_ (4.1 equiv), KI (0.1 equiv), and 2-chloro-*N*,*N*-diethylethylamine hydrochloride (1.2 equiv) in methyl ethyl ketone (5 mL), according to Method A, at reflux temperature, overnight. The residue was diluted with EtOAc, washed with water, and then extracted with 1 M HCl. The pH of the water phase was adjusted to 9 with 1 M NaOH, and the product was extracted with EtOAc (3 × 10 mL). The organic layer was dried over anhydrous Na_2_SO_4_, filtered, and the solvent was evaporated under reduced pressure, providing **98** as a yellow oil in a 26% yield. ^1^H NMR (300 MHz, chloroform-*d*) δ 8.24 (s, 1H), 7.80 (dt, *J* = 1.6, 0.8 Hz, 1H), 7.62–7.51 (m, 2H), 7.49–7.36 (m, 2H), 7.25–7.23 (m, 1H), 7.02–6.92 (m, 2H), 6.59 (ddd, *J* = 3.1, 2.0, 0.8 Hz, 1H), 4.25 (t, *J* = 5.9 Hz, 2H), 3.09 (t, *J* = 5.9 Hz, 2H), 2.87 (q, *J* = 7.3 Hz, 4H), 1.22 (t, *J* = 7.3 Hz, 6H).

#### Synthesis of *tert*-Butyl (*R*)-3-(4-(1-Tosyl-1*H*-indol-5-yl)phenoxy)pyrrolidine-1-carboxylate (**99**)

Under a nitrogen atmosphere, **96** (513 mg, 1.41 mmol) and *tert*-butyl (*S*)-3-hydroxypyrrolidine-1-carboxylate (264 mg, 1.41 mmol) were dissolved in anhydrous THF (5 mL). A solution of triphenylphosphine (444 mg, 1.69 mmol) was added, and the resulting mixture was cooled at −10 °C. A solution of DIAD (332 μL, 1.69 mmol) in anhydrous THF (10 mL) was added dropwise. At the end of addition, the mixture was stirred at reflux overnight. The solvent was evaporated, and the residue was taken in diethyl ether (200 mL), and washed with water and brine. The organic layer was dried over anhydrous Na_2_SO_4_, filtered, and the solvent was concentrated under reduced pressure. Purification by two-column chromatography (first: DCM/EtOAc gradient from 0 to 10% EtOAc; second: toluene/EtOAc 9:1) provided **99** as an off-white solid in a 33% yield. ^1^H NMR (300 MHz, chloroform-*d*) δ 8.01 (dt, *J* = 8.7, 0.8 Hz, 1H), 7.82–7.75 (m, 2H), 7.69–7.63 (m, 1H), 7.57 (d, *J* = 3.6 Hz, 1H), 7.54–7.44 (m, 2H), 7.25–7.14 (m, 3H), 6.97–6.88 (m, 2H), 6.68 (dd, *J* = 3.7, 0.8 Hz, 1H), 4.97–4.85 (m, 1H), 3.71–3.46 (m, 4H), 2.35 (s, 3H), 2.28–2.03 (m, 2H), 1.47 (s, 9H).

#### Synthesis of (*R*)-5-(4-((1-Methylpyrrolidin-3-yl)oxy)phenyl)-1*H*-indole (**100**)

Under a nitrogen atmosphere, a solution of **99** (140 mg, 0.267 mmol) in anhydrous THF (5 mL) was added dropwise to a suspension of LiAlH_4_ (60 mg, 1.58 mmol) at −10 °C. At the end of addition, the reaction mixture was refluxed for 5 h. Upon cooling to 0 °C, water was added and the insoluble mixture was removed by filtration on celite. The solvent was evaporated, the residue was taken in EtOAc, and the organic layer was washed with a saturated solution of Na_2_CO_3_ and brine. The organic phase was dried over anhydrous Na_2_SO_4_, filtered, and the solvent was evaporated under reduced pressure. Purification by flash column chromatography (DCM/MeOH gradient from 0 to 30% MeOH) provided **100** as an off-white solid in an 86% yield. Mp = 146.0–148.4 °C. ^1^H NMR (300 MHz, chloroform-*d*) δ 8.17 (s, 1H), 7.80 (dt, *J* = 1.6, 0.8 Hz, 1H), 7.59–7.50 (m, 2H), 7.48–7.37 (m, 2H), 7.25–7.22 (m, 1H), 7.00–6.86 (m, 2H), 6.59 (ddd, *J* = 3.1, 2.0, 0.8 Hz, 1H), 4.93–4.82 (m, 1H), 2.90–2.81 (m, 3H), 2.56–2.44 (m, 1H), 2.42 (s, 3H), 2.39–2.29 (m, 1H), 2.05 (dddd, *J* = 13.7, 8.1, 6.0, 2.5 Hz, 1H).

#### Synthesis of 4-(Benzofuran-5-yl)phenol (**101**)

Obtained from 5-bromobenzofurane (200 mg, 1.02 mmol, 1 equiv) and *p-*hydroxyphenyl boronic acid (1.1 equiv) according to Method C. The crude was purified by silica gel flash column chromatography (cyclohexane/EtOAc 9:1), providing the desired product **101** as a white solid in an 80% yield. *R*_f_ = 0.2 (cyclohexane/EtOAc 9:1). Mp = 194 °C. ^1^H NMR (300 MHz, chloroform-*d*) δ 7.73 (dd, *J* = 1.9, 0.8 Hz, 1H), 7.64 (d, *J* = 2.2 Hz, 1H), 7.56–7.41 (m, 4H), 6.92 (d, *J* = 8.8 Hz, 2H), 6.80 (dd, *J* = 2.2, 0.8 Hz, 1H), 4.70 (broad s, 1H).

#### Synthesis of 2-(4-(Benzofuran-5-yl)phenoxy)-*N*,*N*-diethylethan-1-amine Hydrochloride (**102**)

Obtained from 4-(benzofuran-5-yl)phenol **101** (170 mg, 0.81 mmol, 1 equiv), K_2_CO_3_ (4 equiv), KI (0.1 equiv), and 2-chloro-*N*,*N*-diethylethylamine hydrochloride (1.2 equiv), in methyl ethyl ketone (5 mL), according to Method A, at reflux temperature, overnight. The residue was diluted with EtOAc, washed with water, and then extracted with 1 M HCl. The pH of the water phase was adjusted to pH 9 with 1 M NaOH and further extracted with EtOAc (3 × 10 mL). The organic layer was dried over anhydrous Na_2_SO_4_, filtered, and the solvent was evaporated under reduced pressure. The residue was taken in diethyl ether (3 mL) and warmed until complete dissolution. A solution of 2 M HCl in diethyl ether (0.5 mL) was added dropwise, and the resulting suspension was filtered, providing the desired compound **102** as a white solid in a 65% yield. Mp = 213 °C. ^1^H NMR (300 MHz, methanol-*d*_4_) δ 7.81–7.74 (m, 2H), 7.65–7.57 (m, 2H), 7.57–7.47 (m, 2H), 7.16–7.07 (m, 2H), 6.88 (s, 1H), 4.48–4.36 (m, 2H), 3.70–3.58 (m, 2H), 3.45–3.35 (m, 4H), 1.40 (t, *J* = 7.4 Hz, 6H).

#### Synthesis of 4-(2-(Diethylamino)ethoxy)benzaldehyde (**103**)

Obtained from *p-*hydroxybenzaldehyde (2.00 g, 16.38 mmol, 1 equiv), K_2_CO_3_ (2.5–4 equiv), and KI (0.1 equiv) and 2-chloro-*N*,*N*-diethylethylamine hydrochloride (1.2 equiv) in methyl ethyl ketone (100 mL), according to Method A, at reflux temperature, overnight. The residue was dissolved in HCl and washed with diethyl ether. The aqueous phase was basified to pH 10 with 1 M NaOH and extracted with EtOAc (3 × 10 mL). The organic phase was dried over anhydrous Na_2_SO_4_, filtered, and the solvent was concentrated under reduced pressure. The crude was purified by silica gel flash column chromatography (DCM/MeOH 9:1 + 1% NH_3(aq30%)_), providing the desired compound **103** as a colorless oil in a 68% yield. *R*_f_ = 0.36 (diisopropyl ether/2-propanol 85:15 + 1% NH_3(aq 30%)_). ^1^H NMR (300 MHz, chloroform-*d*) δ 9.88 (s, 1H), 7.82 (d, *J* = 8.8 Hz, 2H), 7.00 (d, *J* = 8.8 Hz, 2H), 4.12 (t, *J* = 6.2 Hz, 2H), 2.90 (t, *J* = 6.2 Hz, 2H), 2.65 (q, *J* = 7.1 Hz, 4H), 1.07 (t, *J* = 7.1 Hz, 6H).

#### Synthesis of (4-(2-(Diethylamino)ethoxy)phenyl)methanol (**104**)

A solution of 4-(2-(diethylamino)ethoxy)benzaldehyde **103** (1.92 g, 8.68 mmol, 1 equiv) in MeOH (20 mL) was treated with NaBH_4_ (2 equiv) and stirred at room temperature for 3 h. Afterward, the solvent was evaporated under reduced pressure, and the residue was diluted with water (10 mL) and extracted with EtOAc (3 × 20 mL). The organic phase was dried over anhydrous Na_2_SO_4_, filtered, and the solvent was evaporated under reduced pressure. The crude was purified by silica gel flash column chromatography (gradient from DCM to DCM/MeOH 9:1 + 1%NH_3(aq20%)_), providing the desired product **104** as a colorless oil in a 45% yield. *R*_f_ = 0.67 (DCM/MeOH 9:1 + 1%NH_3(aq20%)_). ^1^H NMR (300 MHz, chloroform-*d*) δ 7.27 (d, *J* = 8.2 Hz, 2H), 6.88 (d, *J* = 8.2 Hz, 2H), 4.61 (s, 2H), 4.05 (t, *J* = 6.3 Hz, 2H), 2.89 (t, *J* = 6.3 Hz, 2H), 2.66 (q, *J* = 7.2 Hz, 4H), 1.08 (t, *J* = 7.2 Hz, 6H).

#### Synthesis of *N*,*N*-Diethyl-2-(4-(phenoxymethyl)phenoxy)ethan-1-amine (**105**)

Under an inert atmosphere, a solution of (4-(2-(diethylamino)ethoxy)phenyl)methanol **104** (0.77 g, 3.45 mmol, 1 equiv), PPh_3_ (1.2 equiv), and phenol (1 equiv) in THF (12 mL) was cooled to 0 °C and DEAD (1.2 equiv) was added dropwise. The reaction mixture was stirred at room temperature overnight. The volatiles were evaporated under reduced pressure, and the residue was purified by silica gel flash column chromatography (gradient from DCM to DCM/MeOH 9:1 + 1.5% NH_3(aq20%)_), providing the desired compound **105** as an off-white solid in a 20% yield. Mp = 54.0–57.8 °C. *R*_f_ = 0.6 (DCM/MeOH + 1% NH_3(aq20%)_). ^1^H NMR (300 MHz, chloroform-*d*) δ 7.35 (d, *J* = 8.8 Hz, 2H), 7.35–7.26 (m, 2H), 7.03–6.93 (m, 3H), 6.92 (d, *J* = 8.8 Hz, 2H), 4.98 (s, 2H), 4.07 (t, *J* = 6.3 Hz, 2H), 2.90 (t, *J* = 6.3 Hz, 2H), 2.66 (q, *J* = 7.1 Hz, 4H), 1.09 (t, *J* = 7.1 Hz, 6H).

#### Synthesis of (*E*)-2-((4-(2-(Diethylamino)ethoxy)benzylidene)amino)phenol (**106**)

To a refluxed solution of 4-(2-(diethylamino)ethoxy)benzaldehyde **103** (700 mg, 3.16 mmol, 1 equiv) in EtOH, *o*-aminophenol (1 equiv) was added. The reaction mixture was stirred at reflux temperature for 1 h. The solvent was evaporated under reduced pressure, providing the desired compound **106** as a brown oil, which was used in the next step without further purification. *R*_f_ = 0.6 (diisopropyl ether/2-propanol 85:15 + 1% NH_3(aq 20%)_). ^1^H NMR (300 MHz, chloroform-*d*) δ 8.62 (s, 1H), 7.91–7.83 (m, 2H), 7.31–7.24 (m, 1H), 7.17 (ddd, *J* = 8.1, 7.3, 1.5 Hz, 1H), 7.05–6.96 (m, 3H), 6.89 (ddd, *J* = 8.0, 7.4, 1.4 Hz, 1H), 4.15 (t, *J* = 6.2 Hz, 2H), 2.93 (t, *J* = 6.2 Hz, 2H), 2.69 (q, *J* = 7.1 Hz, 4H), 1.10 (t, *J* = 7.1 Hz, 6H).

#### Synthesis of 2-(4-(Benzo[*d*]oxazol-2-yl)phenoxy)-*N*,*N*-diethylethan-1-amine Hydrochloride (**107**)

A solution of (*E*)-2-((4-(2-(diethylamino)ethoxy)benzylidene)amino)phenol **106** (987 mg, 3.16 mmol) and Pb(OAc)_4_ (1.5 equiv) in EtOH was refluxed for 1 h. The reaction mixture was concentrated under reduced pressure, and the residue was dissolved in MeOH (2 mL) and cooled to 0 °C. Under vigorous stirring, a methanolic solution of 4 M HCl (0.2 mL) was added dropwise. After 30 min, the reaction mixture was diluted with diethyl ether, and the resulting suspension was filtered, affording the desired product **107** as a white solid in a 30% yield. Mp = 192–193 °C. ^1^H NMR (300 MHz, methanol-*d*_4_) δ 8.23 (d, *J* = 9.0 Hz, 2H), 7.73–7.68 (m, 1H), 7.68–7.63 (m, 1H), 7.42–7.37 (m, 2H), 7.23 (d, *J* = 9.0 Hz, 2H), 4.54–4.44 (m, 2H), 3.72–3.63 (m, 2H), 3.38 (q, *J* = 7.3 Hz, 4H), 1.40 (t, *J* = 7.3 Hz, 6H).

#### Synthesis of 2-(4-(1*H*-Benzo[*d*]imidazol-2-yl)phenoxy)-*N*,*N*-diethylethan-1-amine (**108**)

A solution of 4-(2-(diethylamino)ethoxy)benzaldehyde **103** (610 mg, 2.76 mmol, 1 equiv) in EtOH (3 mL) was heated to reflux temperature, and *o-*phenylenediamine (300 mg, 2.76 mmol, 1 equiv) and Pb(OAc)_4_ were added. The reaction mixture was stirred at reflux temperature overnight. Upon cooling, the volatiles were removed under reduced pressure, and the residue was purified by silica gel flash column chromatography (EtOAc/2-propanol gradient from 0 to 5% 2-propanol + 3% NH_3(aq20%)_). Product **108** was obtained as a light brown solid in a 44% yield. *R*_f_ = 0.15 (diisopropyl ether/2-propanol 85:15 + 1% NH_3(aq20%)_). Mp = 189.3–192.1 °C (coherent with the literature^32^). ^1^H NMR (300 MHz, chloroform-*d*) δ 7.98 (d, *J* = 8.8 Hz, 2H), 7.67–7.57 (m, 2H), 7.25–7.21 (m, 2H), 6.94 (d, *J* = 8.8 Hz, 2H), 4.16 (t, *J* = 5.9 Hz, 2H), 2.99 (t, *J* = 5.9 Hz, 2H), 2.77 (q, *J* = 7.2 Hz, 4H), 1.14 (t, *J* = 7.2 Hz, 6H).

#### Synthesis of 1-Iodo-3-(2-methoxyethoxymethyloxy)benzene (**109**)

Under a nitrogen atmosphere at 0 °C, MEMCl (0.44 mL, 3.82 mmol) was added dropwise to a solution of 3-iodophenol (600 mg, 2.73 mmol) and DIPEA (0.84 mL, 4.84 mmol) in DCM (4 mL). Upon stirring at 35 °C for 5 h, the mixture was quenched with a saturated NH_4_Cl solution at 0 °C. The aqueous layer was extracted with EtOAc (3 × 10 mL). The combined organic layers were washed with 1 M HCl, saturated NaHCO_3_ solution, and brine, and the organic phase was dried over anhydrous Na_2_SO_4_, filtered, and the solvent was evaporated under vacuum obtaining **109** as a pale-yellow oil in a 96% yield. *R*_f_ = 0.5 (cyclohexane/EtOAc 9:1). ^1^H NMR (300 MHz, chloroform-*d*): δ 7.45–7.39 (m, 1H), 7.36–7.29 (m, 1H), 7.05–6.94 (m, 2H), 5.24 (s, 2H), 3.86–3.75 (m, 2H), 3.63–3.49 (m, 2H), 3.38 (s, 3H).

### Biological Assays

All methods are the same as those used in our recent publication.^7^ We provide brief outlines of these approaches next.

### Binding Affinity to α7, α3β4, and α4β2 Nicotinic Receptors

For (±)-[^3^H]epibatidine (specific activity of 56–60 Ci/mmol; Perkin Elmer, Boston, MA), saturation binding studies were carried out on membrane homogenates. These were prepared from either SH-EP1 cells stably transfected with α3- and β4-nAChR subunit cDNAs^8^ or HEK 293 cells stably transfected with the α4 and β2 cDNAs (generous gift of Dr. Jon Lindstrom).^9^

For saturation experiments, the membrane homogenate aliquots were incubated overnight at 4 °C with 0.01–5 nM concentrations of (±)-[^3^H]epibatidine. Nonspecific binding was determined in parallel by adding 100 nM unlabeled epibatidine (Sigma-Aldrich) to the incubation solutions, as described previously.^33^ At the end of the incubation, the samples were filtered on a GFC filter soaked in 0.5% polyethylenimine and washed with 10 mL of ice-cold phosphate-buffered saline (PBS) and the filters were counted in a β counter.

For [^125^I]-αBungarotoxin ([^125^I]αBgtx) (specific activity 200–213 Ci/mmol, Perkin Elmer, Boston, MA), saturation binding studies were carried out on a membrane homogenate prepared from SH-SY5Y cells transfected with human α7 cDNA, as described previously.^5^ Aliquots of the membrane homogenates were incubated overnight with 0.1–10.0 nM concentrations of [^125^I]Bgtx at rt. Nonspecific binding was determined in parallel by including in the assay mixture 1 μM of unlabeled αBgtx (Sigma-Aldrich). After incubation, the samples were filtered as described for (±)-[^3^H]epibatidine binding.

For competition studies, the inhibition of [^3^H]epibatidine and [^125^I] αBgtx binding was measured by incubating the membranes transfected with the appropriate subtype with increasing concentrations of the compounds (1 nM to 1 mM) 5 min followed by overnight incubation at 4 °C, with 0.1 nM of [^3^H]epibatidine for the α4β2 subtype or 0.25 nM of [^3^H]epibatidine for the α3β4 subtype or at rt with 2–3 nM of [^125^I]αBgtx in the case of the α7-subtype. At the end of the incubation time, the samples were processed as described for the saturation studies.

[^3^H]epibatidine binding was determined by liquid scintillation counting in a β counter, and [^125^I] αBgtx binding was determined by direct counting in a γ counter. Saturation binding data were evaluated by one-site competitive binding curve-fitting procedures using GraphPad Prism version 6 (GraphPad Software, CA). In the saturation binding assay, the maximum specific binding (*B*_max_) and the equilibrium binding constant (*K*_d_) values were calculated using one-site—specific binding with the Hill slope—model. *K*_i_ values were obtained by fitting three independent competition binding experiments, each performed in duplicate for each compound on each subtype. Inhibition constants (*K*_i_) were estimated by reference to the *K*_d_ of the radioligand, according to the Cheng–Prusoff equation and are expressed as nM values.

### Two-Electrode Voltage Clamp (TEVC) Recording of α7- and α9α10-nAChR Functions

For functional pharmacology studies, two-electrode voltage clamp recordings were performed, using human nAChR subunits heterologously expressed in *X. laevis* oocytes. Approaches were closely related to those previously detailed.^10^ Briefly, *X. laevis* oocytes were purchased from Ecocyte Bioscience US (Austin, TX), and the incubation temperature was 13 °C. Harvesting of oocytes from *X. laevis* by EcoCyte follows the guidelines of the National Institute of Health’s Office of Laboratory Animal Welfare and was authorized under IACUC number #1019-1 (valid through December 2022). Injections of nAChR subunit mRNA were made using glass micropipettes (outer diameter ≈40 μm, resistance 2–6 MΩ), and mRNA was injected in a total volume of 40 nL. For α7-nAChR, 1.25 ng of α7-nAChR subunit mRNA was injected per oocyte along with 0.125 ng of NACHO mRNA to improve functional expression.^34^ For α9α10-nAChR, a total of 10 ng of nAChR subunit mRNA was injected using α9 to α10 cRNAs in a 9:1 ratio by mass.

TEVC recordings were made in oocyte saline solution (82.5 mM NaCl, 2.5 mM KCl, 5 mM HEPES, 1.8 mM CaCl_2_·_2_H_2_O, and 1 mM MgCl_2_·_6_H_2_O, pH 7.4) and were performed at room temperature (20 °C). One week after injection, oocytes were voltage-clamped (−70 mV; Axoclamp 900A amplifier, Molecular Devices, Sunnyvale, CA). Recordings were sampled at 10 kHz (low-pass Bessel filter, 40 Hz; high-pass filter, DC) and saved to disk (Clampex v10.2; Molecular Devices). To ensure the quality of recordings, oocytes with leak currents (*I*_leak_) > 50 nA were discarded without being recorded. In all cases, initial control stimulations (ACh, 1 mM, applied for 1 s) were performed, with a 60 s washout (no drug) between control stimulations (total of five stimulations). This allowed us to define a 100% response control and to ascertain that run-down or desensitization was not occurring due to repeated ACh stimulation.

For antagonist concentration response curves, test compounds were applied simultaneously with 1 mM ACh, starting with the lowest concentration of the test compound and increasing in half-log steps to a maximum concentration of 100 μM. The standard 1 min spacing between stimulation was maintained. Data for each oocyte were normalized by expressing the peak function in the presence of test compounds as % of the control function (the mean peak function measured across the initial control stimulations was defined as 100% for each oocyte). IC_50_ values were calculated from these normalized nAChR-mediated currents through nonlinear least-squares curve fitting (GraphPad Prism 5.0; GraphPad Software, Inc., La Jolla, CA).

The intrinsic agonist efficacy of test compounds was measured by applying them (alone at 100 μM, 1 s application time, no ACh coapplication) 1 min following the last initial control stimulation. The peak function following the addition of the test compound was normalized for each oocyte in the same way just described for antagonist concentration curves. The same normalization was applied to the peak of any rebound current observed during the 60 s washout period following the application of the test compound and to the peak function induced by a final control application of ACh (1 mM, 1 s application time).

### Computational Modeling

Compounds **1a** and **33** were drawn with the two-dimensional (2D) sketch editor of Maestro and prepared for docking using Ligprep, with default settings. The dimeric α7α7 interface containing EVP-6124 was extracted from the cryo-EM of the full-length structure of the human α7-nAChR (7EKP) and prepared with the Protein Preparation Wizard according to default settings. Compound **33** was docked using the Induced Fit Protocol of Schrodinger,^35^ selecting the current ligand (EVP-6124) as the docking centroid, Glide XP redocking, and a scaling factor of 1.0, to avoid excessive deformation of the binding site. The best-scoring pose according to the IFD score and the XP GScore also respected the best-known conserved ligand−α7-nAChR interaction, by placing the positively charged nitrogen within the aromatic box and was therefore selected. Compound **1a** was docked using Glide XP docking with default settings, with a grid centered on ligand **33**, and the best-scored pose according to the XP GScore was selected. The binding site analysis was performed using Sitemap, centered on **33** and default settings.

## Supporting Information Available

^1^H NMR and ^13^C NMR of the final compounds; HPLC traces of key final compounds (**6**, **28**, and **33**); example traces of two-electrode voltage clamp recordings (PDF)
Molecular formula strings (CSV)

## Abbreviations Used

*B*_max_: maximum specific binding
cryo-EM: cryogenic electron microscopy
DIAD: diisopropyl azodicarboxylate
eq: equivalent
*K*_d_: equilibrium binding constant
*K*_i_: inhibition constant
nAChR: nicotinic acetylcholine receptor
*R*_t_: retention time
S.E.M.: standard error of mean
