# Supplementary material for: Subnanomolar Affinity and Selective Antagonism at α7 Nicotinic Receptor by Combined Modifications of 2-Triethylammonium Ethyl Ether of 4-Stilbenol (MG624)
Source: J Med Chem. 2022 Dec 16;66(1):306–32. doi: 10.1021/acs.jmedchem.2c01256 (PMC9841521; doi:10.1021/acs.jmedchem.2c01256)
Supplement: Supplementary file 1 — jm2c01256_si_001.pdf [file jm2c01256_si_001.pdf]

Supporting Information

Sub-nanomolar affinity and selective antagonism at α7 nicotinic receptor by combined modifications of 2-triethylammonium ethyl ether of 4-stilbenol (MG624)

Francesco Bavo, [†,#] *Marco Pallavicini,* [†]*Susanna Pucci,* [‡,°] *Rebecca Appiani,* [†] *Alessandro Giraudo,* [†] *Hyoungil Oh,* [•] *Dana L. Kneisley,* [•] *Brek Eaton,* [ǂ] *Linda Lucero,* [ǂ] *Cecilia Gotti,*[‡] *Francesco Clementi,* [‡] *Paul Whiteaker,* [•] *and Cristiano Bolchi*[*,†]

[†] Dipartimento di Scienze Farmaceutiche, Università degli Studi di Milano, via Mangiagalli 25, I-20133 Milano, Italy

[#] Department of Drug Design and Pharmacology, University of Copenhagen, DK-2100 Copenhagen, Denmark

[‡] Institute of Neuroscience, CNR, via Vanvitelli 32, I-20129 Milano, Italy

[°] NeuroMi Milan Center for Neuroscience, University of Milano Bicocca, piazza Ateneo Nuovo 1, I-20126 Milano, Italy

[ǂ] Division of Neurobiology, Barrow Neurological Institute, Phoenix, AZ 85013, USA

[•] Department of Pharmacology and Toxicology, Medical College of Virginia Campus, Virginia Commonwealth University, Richmond, VA 23298, USA

Corresponding author's email address: cristiano.bolchi@unimi.it

Index

^{1}H NMR and ^{13}C NMR spectra of final compounds .... **Error! Bookmark not defined.**
N,N,N-triethyl-2-phenoxyethan-1-aminium iodide (**3**). .... **Error! Bookmark not defined.**
N,N,N-triethyl-2-(4-vinylphenoxy)ethan-1-aminium iodide (**4**). .... **Error! Bookmark not defined.**
2-([1,1'-biphenyl]-4-yloxy)-N,N,N-triethylethan-1-aminium iodide (**5**). .... **Error! Bookmark not defined.**
N,N,N-triethyl-2-(4-phenethylphenoxy)ethan-1-aminium iodide (**6**). .... **Error! Bookmark not defined.**
N,N,N-triethyl-2-(4-(phenylethynyl)phenoxy)ethan-1-aminium iodide (**7**). .... **Error! Bookmark not defined.**
N,N,N-triethyl-2-(4-(naphthalen-2-yl)phenoxy)ethan-1-aminium iodide (**8**). .... **Error! Bookmark not defined.**
N,N,N-triethyl-2-(4-(naphthalen-1-yl)phenoxy)ethan-1-aminium iodide (**9**). .... **Error! Bookmark not defined.**
(E)-N,N,N-triethyl-2-(4-(2-(naphthalen-1-yl)vinyl)phenoxy)ethan-1-aminium iodide (**10**). .... **Error! Bookmark not defined.**
(E)-N,N,N-triethyl-2-(4-(2-(naphthalen-2-yl)vinyl)phenoxy)ethan-1-aminium iodide (**11**). .... **Error! Bookmark not defined.**
(E)-2-(4-(2-bromostyryl)phenoxy)-N,N,N-triethylethan-1-aminium iodide (**12**). .... **Error! Bookmark not defined.**
(E)-2-(4-(3-bromostyryl)phenoxy)-N,N,N-triethylethan-1-aminium iodide (**13**). .... **Error! Bookmark not defined.**
(E)-2-(4-(4-bromostyryl)phenoxy)-N,N,N-triethylethan-1-aminium iodide (**14**). .... **Error! Bookmark not defined.**
(E)-N,N,N-triethyl-2-(4-(3-(trifluoromethyl)styryl)phenoxy)ethan-1-aminium iodide (**15**) ..... **Error! Bookmark not defined.**
(E)-N,N,N-triethyl-2-(4-(4-(trifluoromethyl)styryl)phenoxy)ethan-1-aminium iodide (**16**) ..... **Error! Bookmark not defined.**
(E)-N,N,N-triethyl-2-(4-(3-methoxystyryl)phenoxy)ethan-1-aminium iodide (**17**) ... **Error! Bookmark not defined.**
(E)-N,N,N-triethyl-2-(4-(4-methoxystyryl)phenoxy)ethan-1-aminium iodide (**18**) ... **Error! Bookmark not defined.**
(E)-N,N,N-triethyl-2-(4-(3-hydroxystyryl)phenoxy)ethan-1-aminium iodide (**19**) ..... **Error! Bookmark not defined.**
(E)-N,N,N-triethyl-2-(4-(4-hydroxystyryl)phenoxy)ethan-1-aminium iodide (**20**) ..... **Error! Bookmark not defined.**
(E)-2-(4-(3,5-dihydroxystyryl)phenoxy)-N,N,N-triethylethan-1-aminium iodide (**22**) .... **Error! Bookmark not defined.**
2-(4-(benzyloxy)phenoxy)-N,N,N-triethylethan-1-aminium iodide (**23**) .... **Error! Bookmark not defined.**
N,N,N-triethyl-2-(4-(phenoxymethyl)phenoxy)ethan-1-aminium iodide (**24**) .... **Error! Bookmark not defined.**
2-(4-benzamidophenoxy)-N,N,N-triethylethan-1-aminium iodide (**25**). .... **Error! Bookmark not defined.**
N,N,N-triethyl-2-(4-(phenylcarbamoyl)phenoxy)ethan-1-aminium iodide (**26**) .... **Error! Bookmark not defined.**
(E)-N,N,N-triethyl-2-(4-(phenyldiazenyl)phenoxy)ethan-1-aminium iodide (**27**) ...... **Error! Bookmark not defined.**
2-(4-(benzo[d]oxazol-2-yl)phenoxy)-N,N,N-triethylethan-1-aminium iodide (**28**) ... **Error! Bookmark not defined.**
2-(4-(1H-benzo[d]imidazol-2-yl)phenoxy)-N,N,N-triethylethan-1-aminium iodide (**29**). .... **Error! Bookmark not defined.**
2-(4-(1H-indol-6-yl)phenoxy)-N,N,N-triethylethan-1-aminium iodide (**30**). .... **Error! Bookmark not defined.**
2-(4-(1H-indol-5-yl)phenoxy)-N,N,N-triethylethan-1-aminium iodide (**31**). .... **Error! Bookmark not defined.**
2-(4-(benzofuran-5-yl)phenoxy)-N,N,N-triethylethan-1-aminium iodide (**32**). .... **Error! Bookmark not defined.**
(R)-3-(4-(1H-indol-5-yl)phenoxy)-1,1-dimethylpyrrolidin-1-ium iodide (**33**). .... **Error! Bookmark not defined.**
HPLC analysis of key final compounds .... **Error! Bookmark not defined.**
N,N,N-triethyl-2-(4-phenethylphenoxy)ethan-1-aminium iodide (**6**). .... **Error! Bookmark not defined.**
2-(4-(benzo[d]oxazol-2-yl)phenoxy)-N,N,N-triethylethan-1-aminium iodide (**28**) ... **Error! Bookmark not defined.**
(R)-3-(4-(1H-indol-5-yl)phenoxy)-1,1-dimethylpyrrolidin-1-ium iodide (**33**). .... **Error! Bookmark not defined.**
Example traces of two-electrode voltage-clamp recordings (data for Figs 2 and 3). 20
Compound 2, α9α10-nAChR 20
Compound 6, α9α10-nAChR 21
Compound 28, α9α10-nAChR 22
Compound 33, α9α10-nAChR 23
Compound 2, α7-nAChR 24
Compound 28, α7-nAChR 25

Compound 33, α7-nAChR……..26

^{1}H NMR and ^{13}C NMR spectra of final compounds

*N,N,N-triethyl-2-phenoxyethan-1-aminium iodide (**3**).*

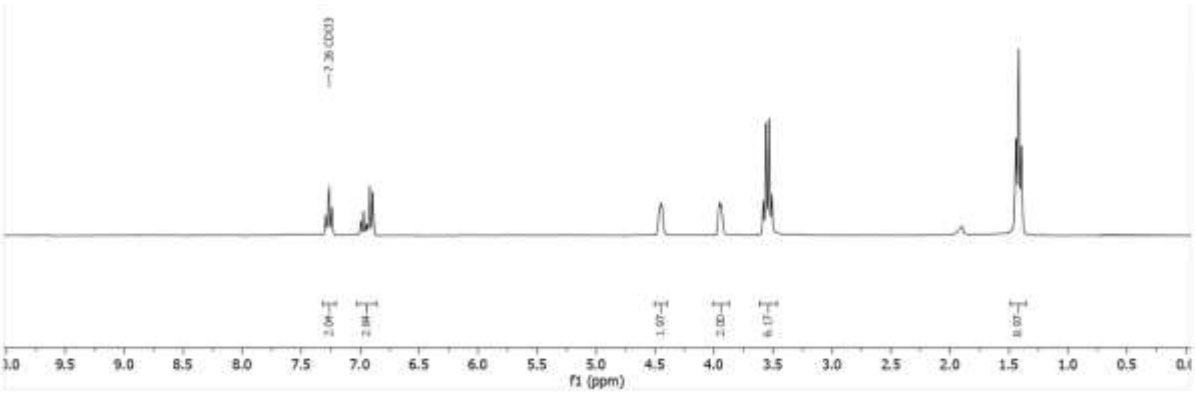

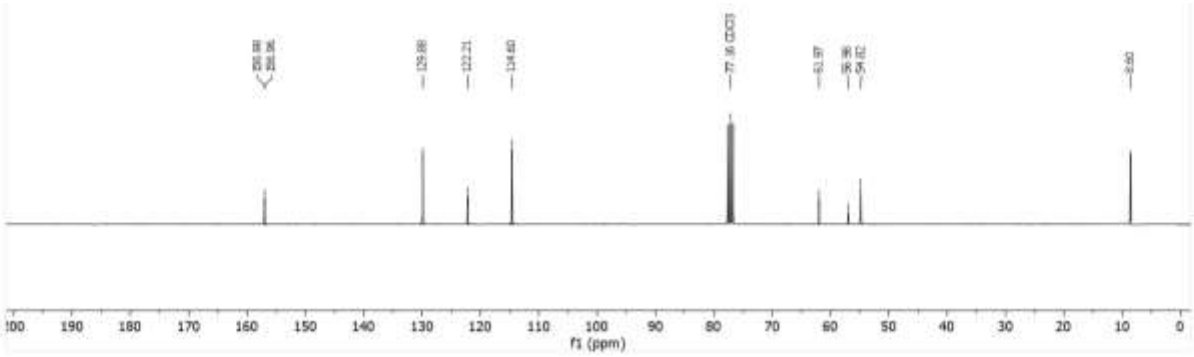

*N,N,N-triethyl-2-(4-vinylphenoxy)ethan-1-aminium iodide (**4**).*

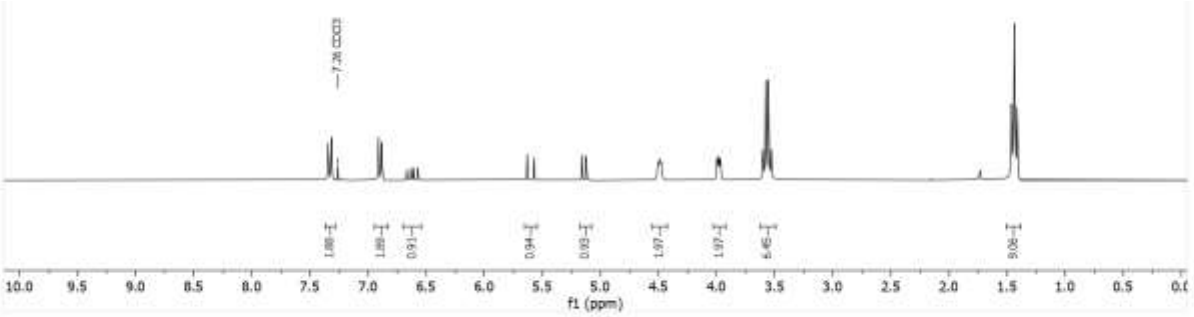

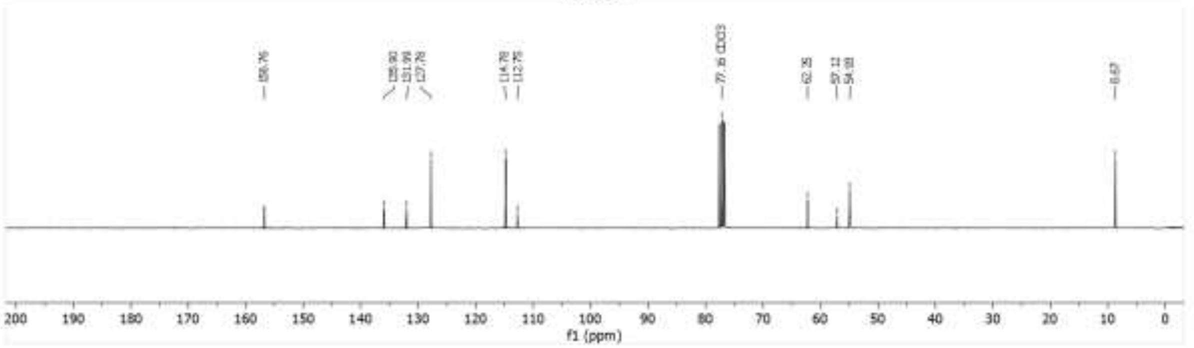

*2-([1,1'-biphenyl]-4-yloxy)-N,N,N-triethylethan-1-aminium iodide (**5**).*

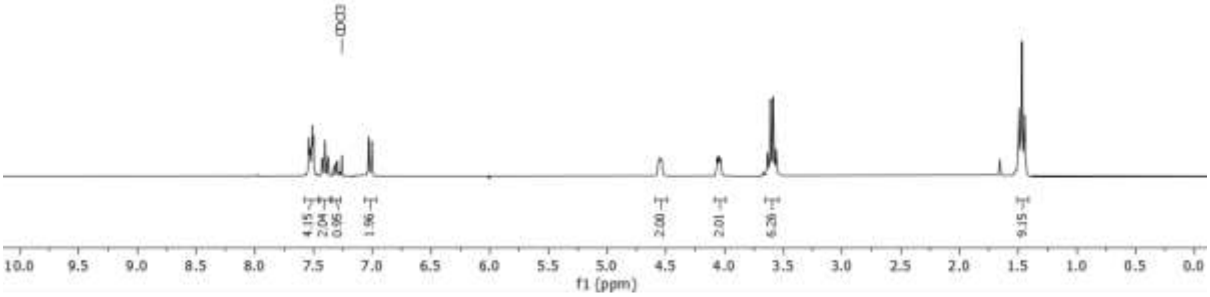

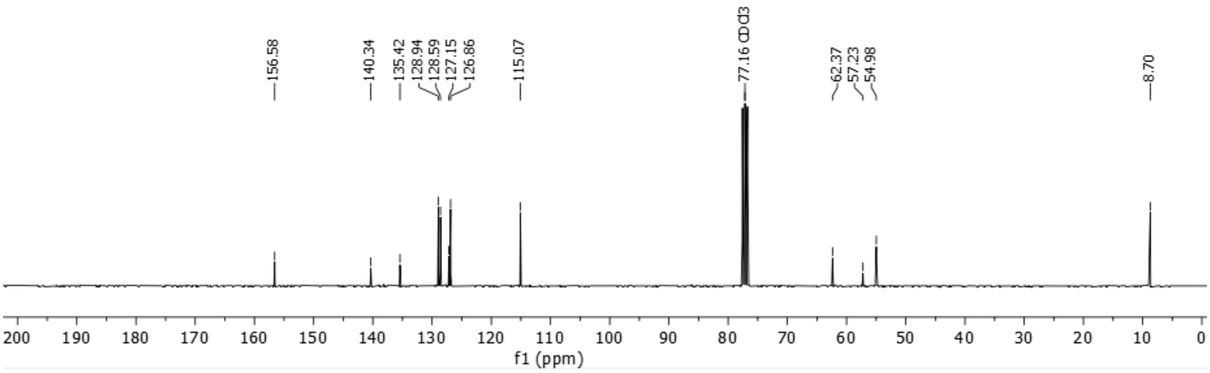

*N,N,N-triethyl-2-(4-phenethylphenoxy)ethan-1-aminium iodide (**6**).*

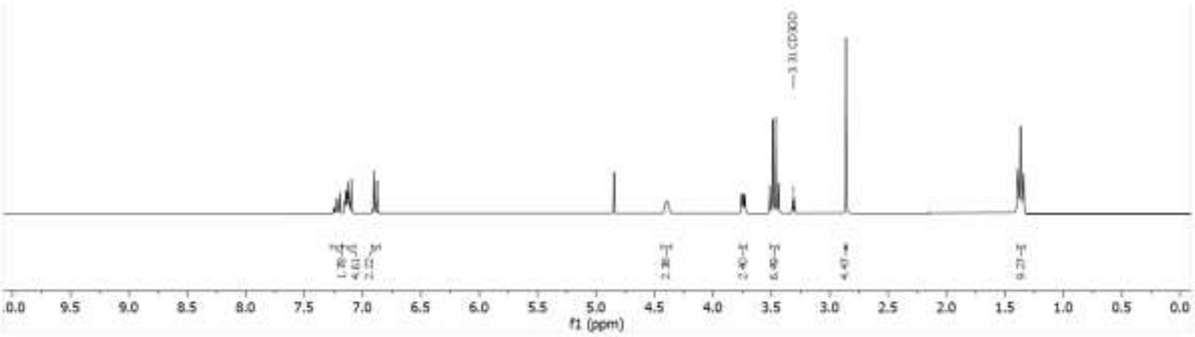

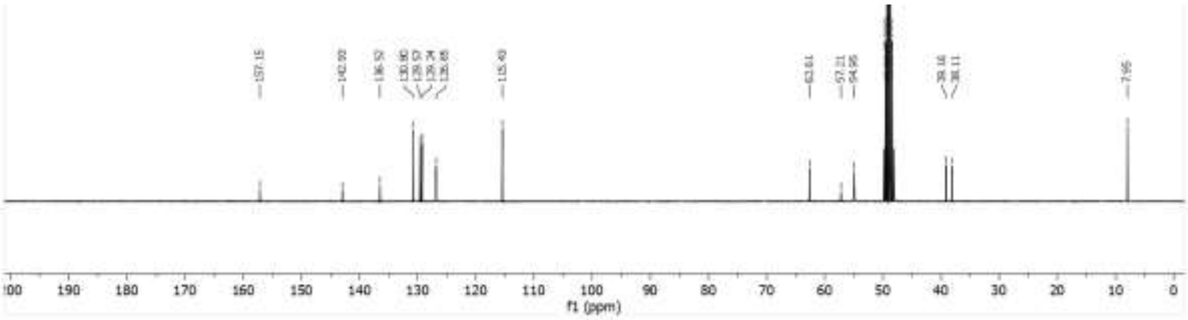

N,N,N-triethyl-2-(4-(phenylethynyl)phenoxy)ethan-1-aminium iodide (7).

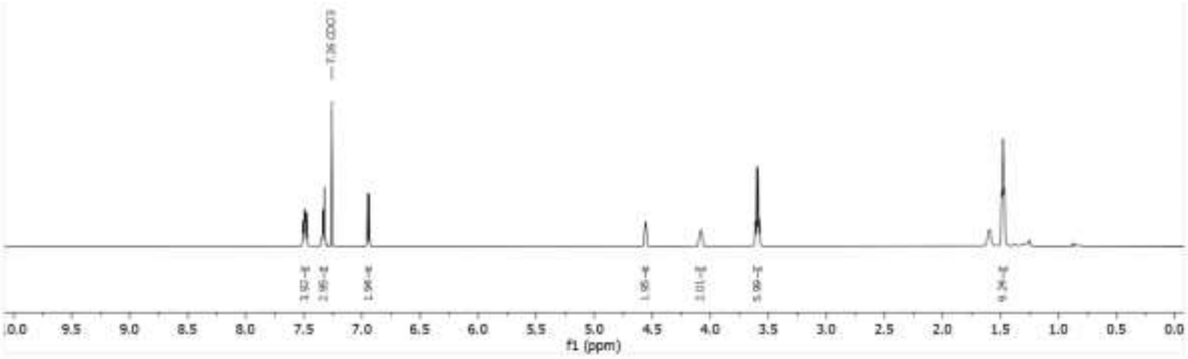

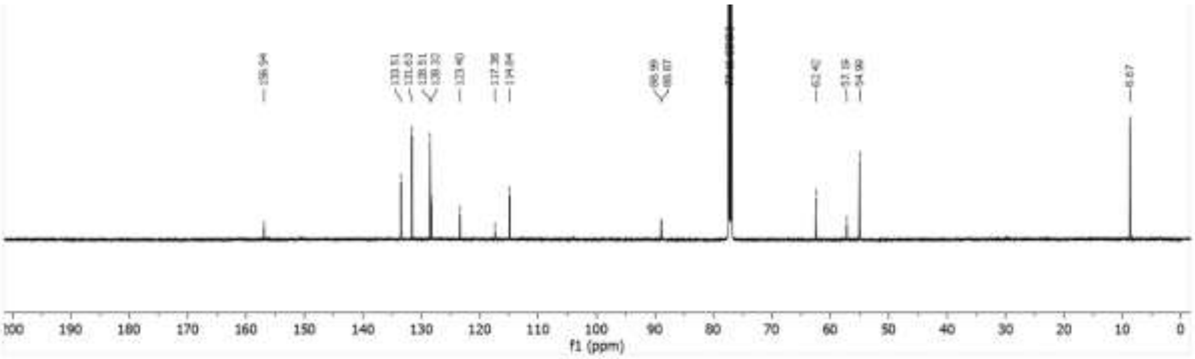

*N,N,N-triethyl-2-(4-(naphthalen-2-yl)phenoxy)ethan-1-aminium iodide (**8**).*

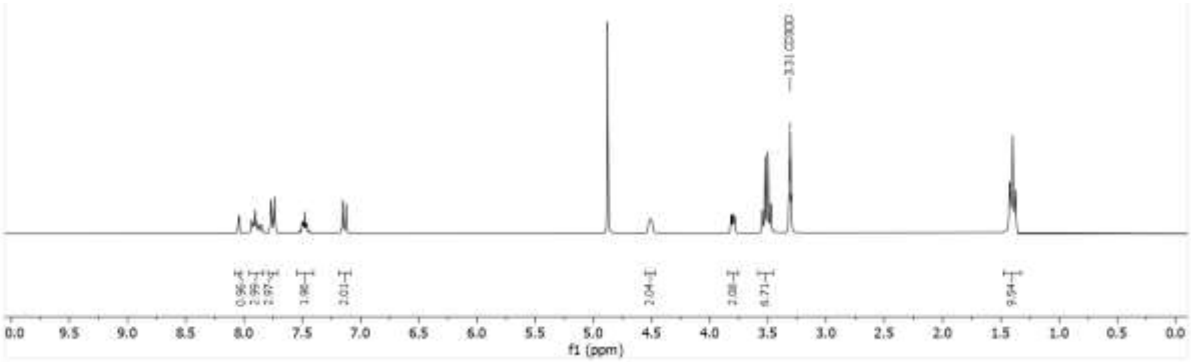

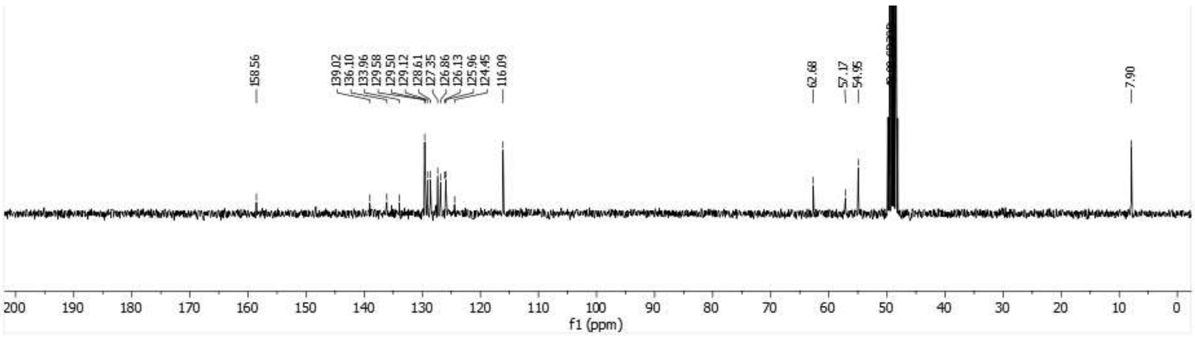

*N,N,N-triethyl-2-(4-(naphthalen-1-yl)phenoxy)ethan-1-aminium iodide (**9**).*

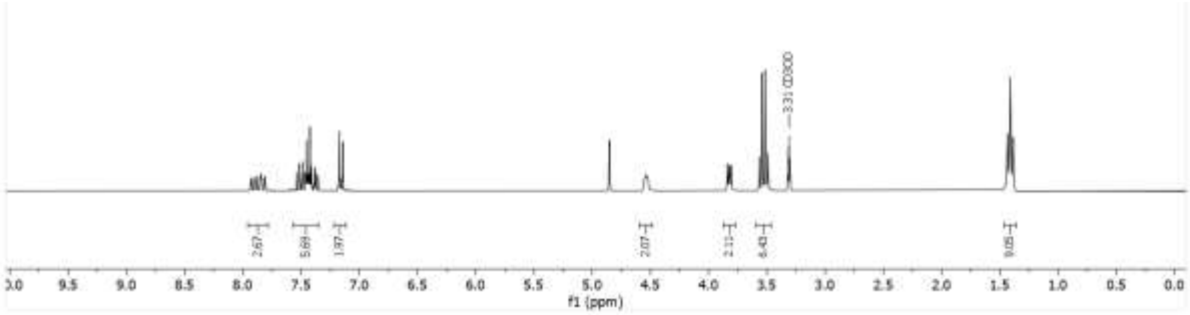

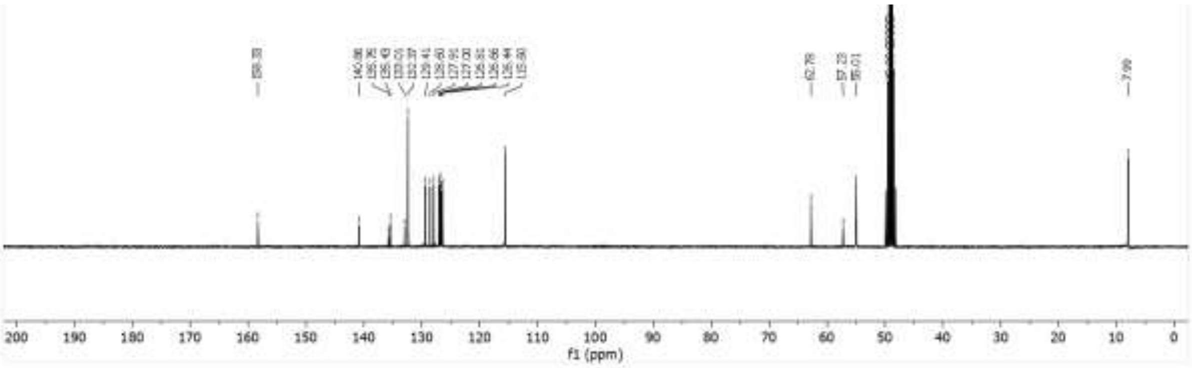

*(E)-N,N,N-triethyl-2-(4-(2-(naphthalen-1-yl)vinyl)phenoxy)ethan-1-aminium iodide (**10**).*

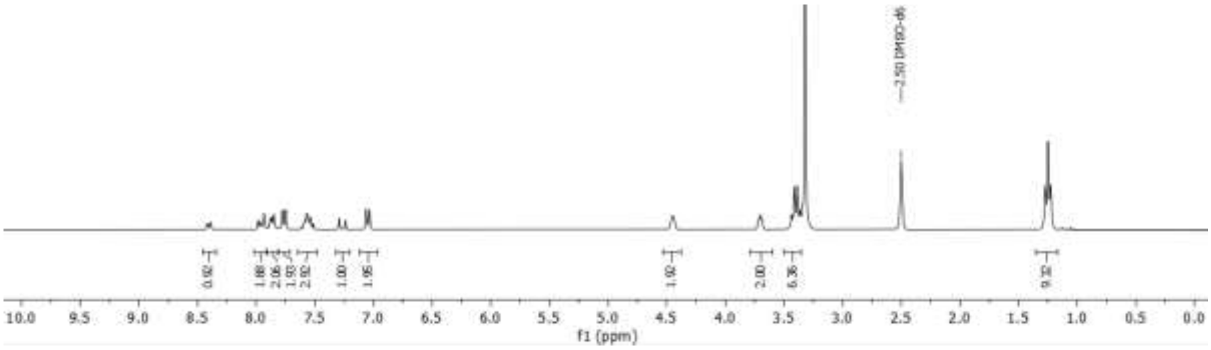

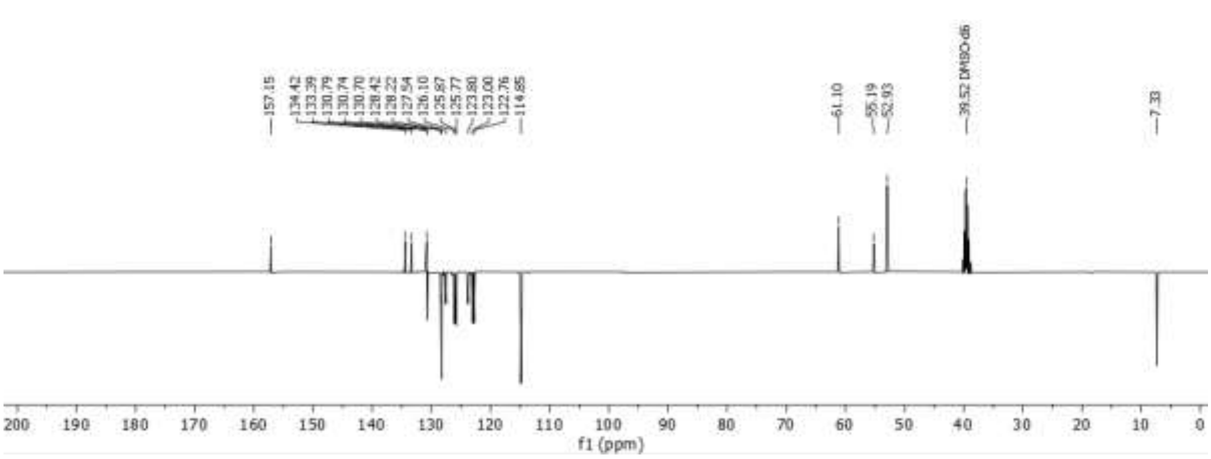

*(E)-N,N,N-triethyl-2-(4-(2-(naphthalen-2-yl)vinyl)phenoxy)ethan-1-aminium iodide (**11**).*

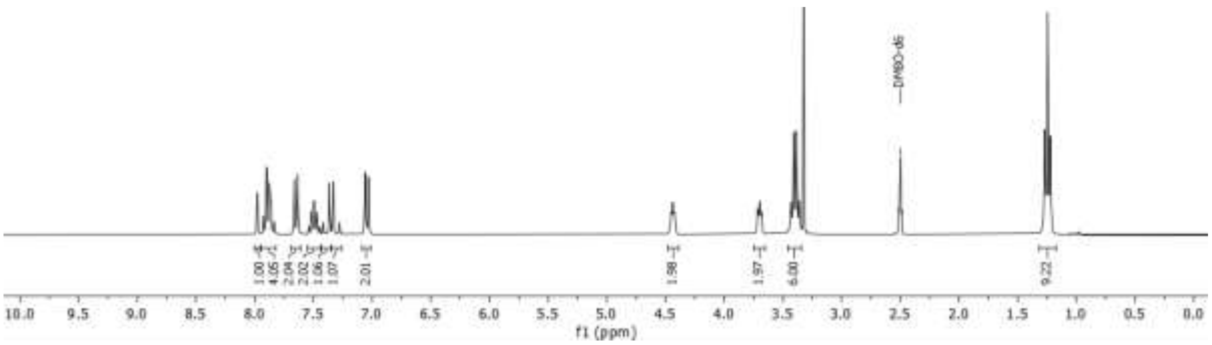

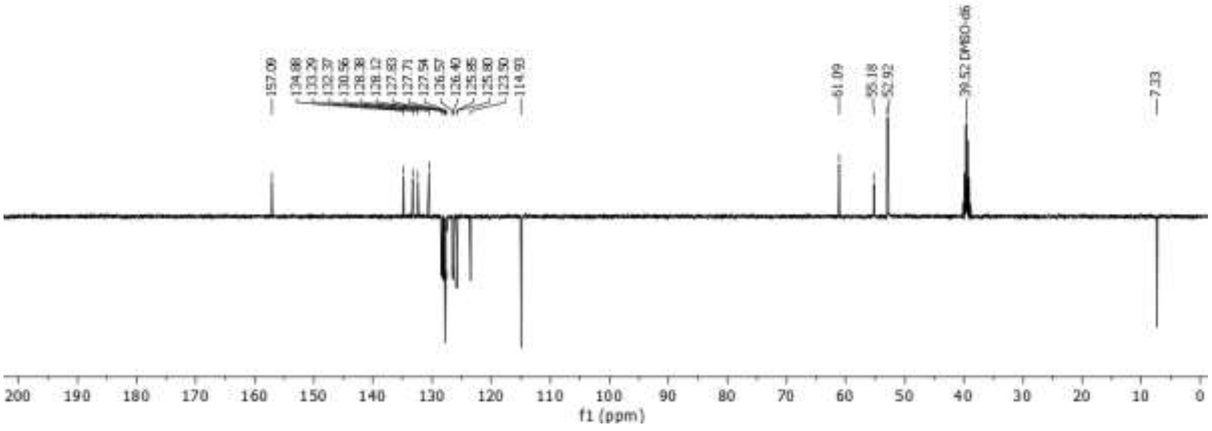

*(E)-2-(4-(2-bromostyryl)phenoxy)-N,N,N-triethylethan-1-aminium iodide (**12**).*

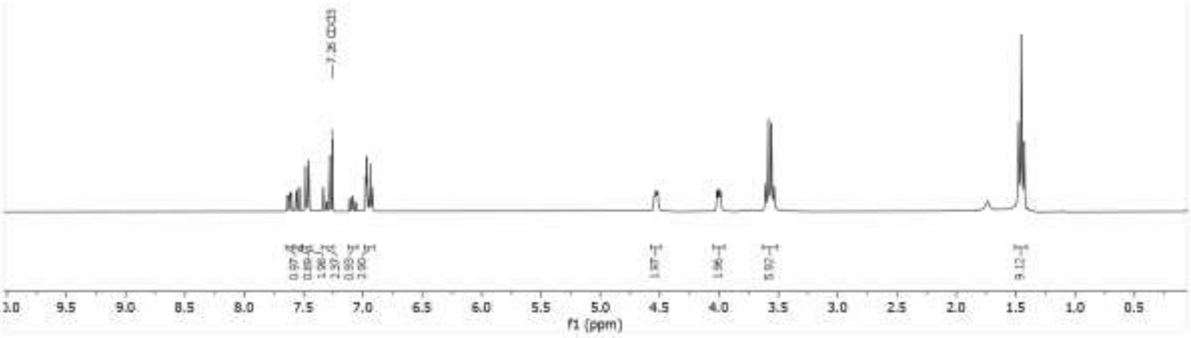

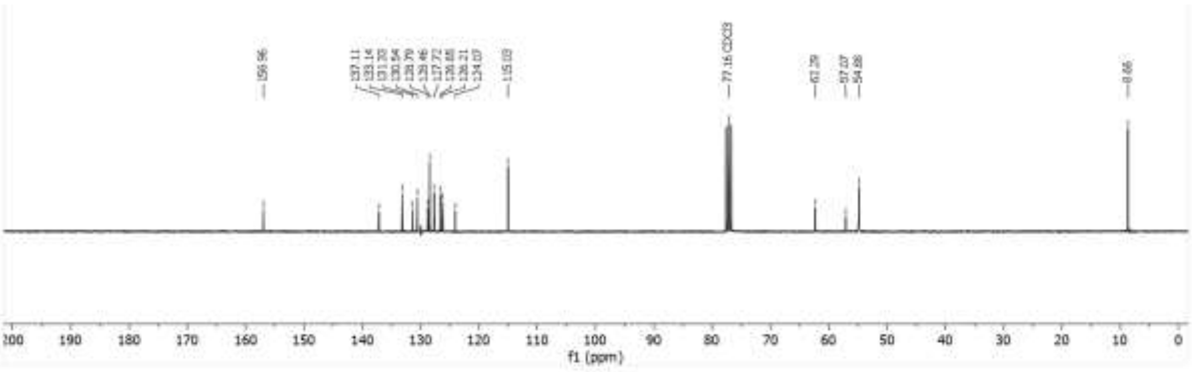

*(E)-2-(4-(3-bromostyryl)phenoxy)-N,N,N-triethylethan-1-aminium iodide (**13**).*

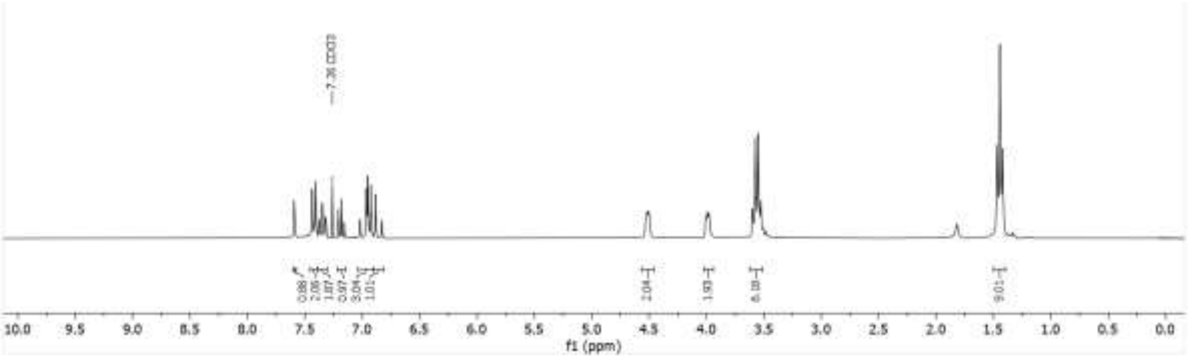

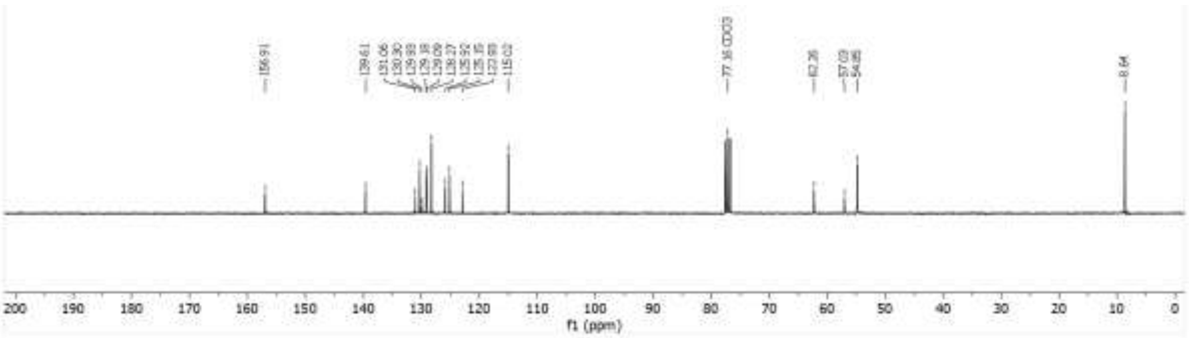

*(E)-2-(4-(4-bromostyryl)phenoxy)-N,N,N-triethylethan-1-aminium iodide (**14**).*

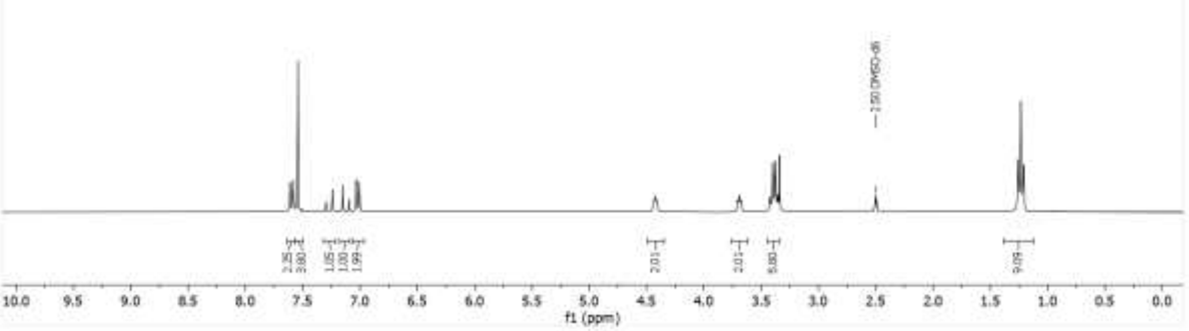

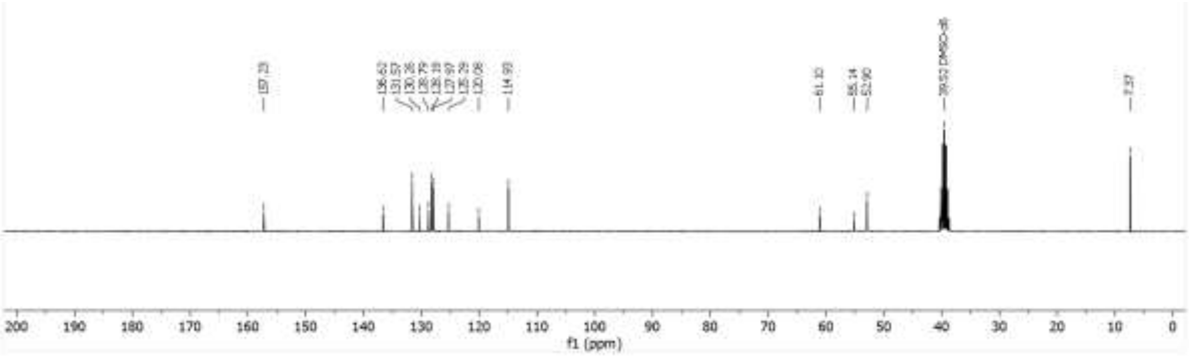

*(E)-N,N,N-triethyl-2-(4-(3-(trifluoromethyl)styryl)phenoxy)ethan-1-aminium iodide (**15**).*

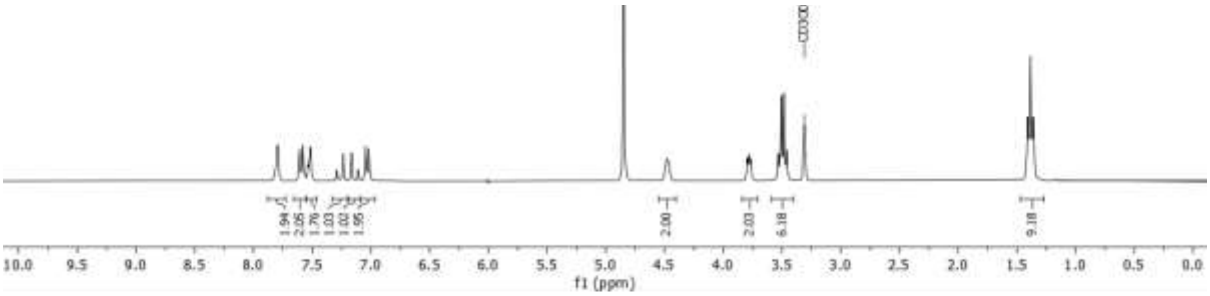

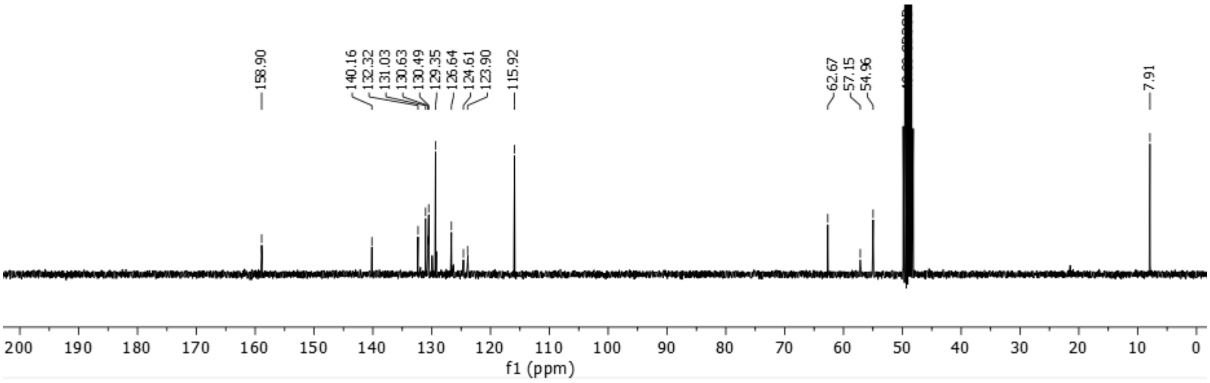

*(E)-N,N,N-triethyl-2-(4-(4-(trifluoromethyl)styryl)phenoxy)ethan-1-aminium iodide (**16**).*

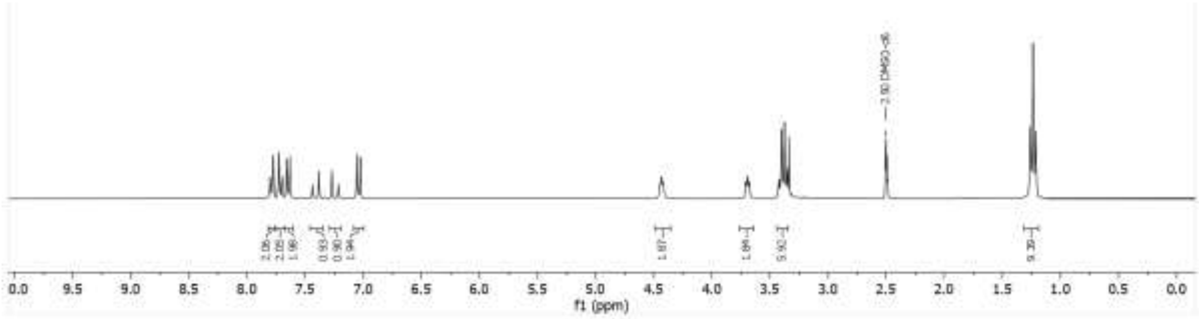

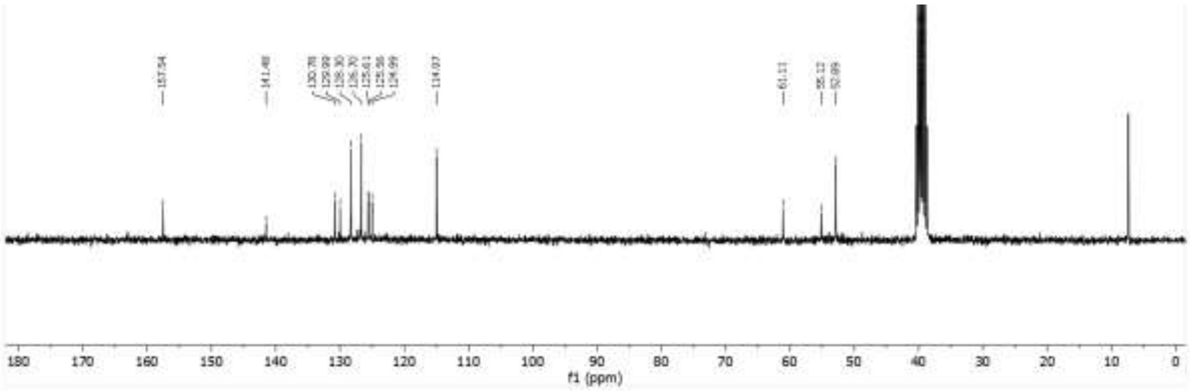

*(E)-N,N,N-triethyl-2-(4-(3-methoxystyryl)phenoxy)ethan-1-aminium iodide (**17**).*

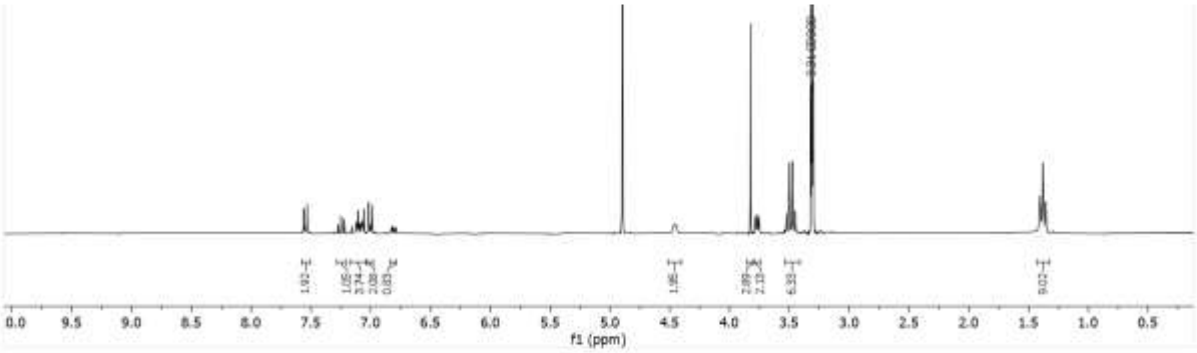

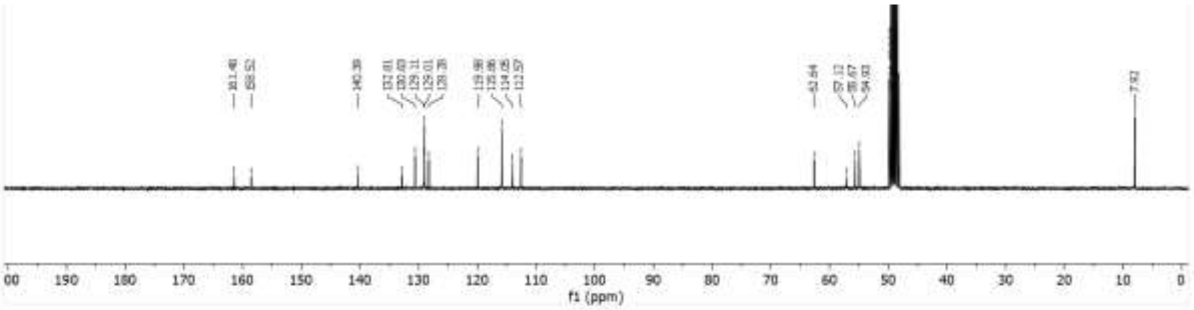

*(E)-N,N,N-triethyl-2-(4-(4-methoxystyryl)phenoxy)ethan-1-aminium iodide (**18**).*

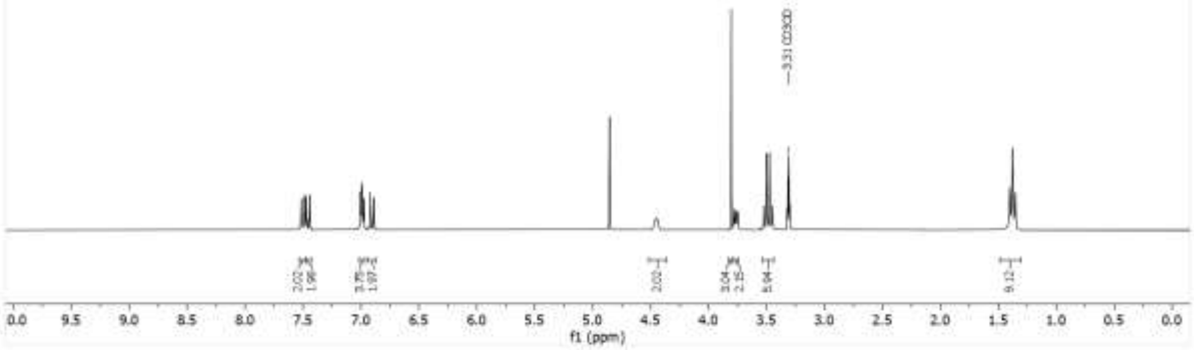

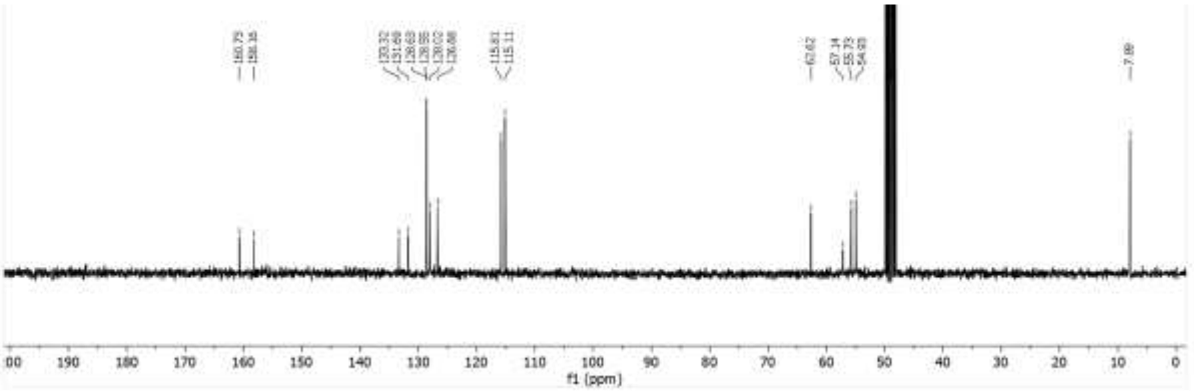

*(E)-N,N,N-triethyl-2-(4-(3-hydroxystyryl)phenoxy)ethan-1-aminium iodide (**19**).*

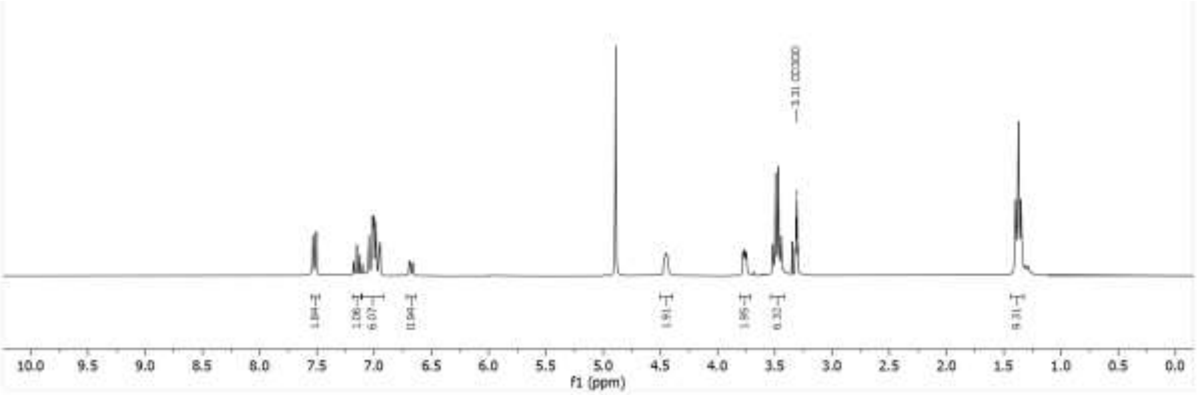

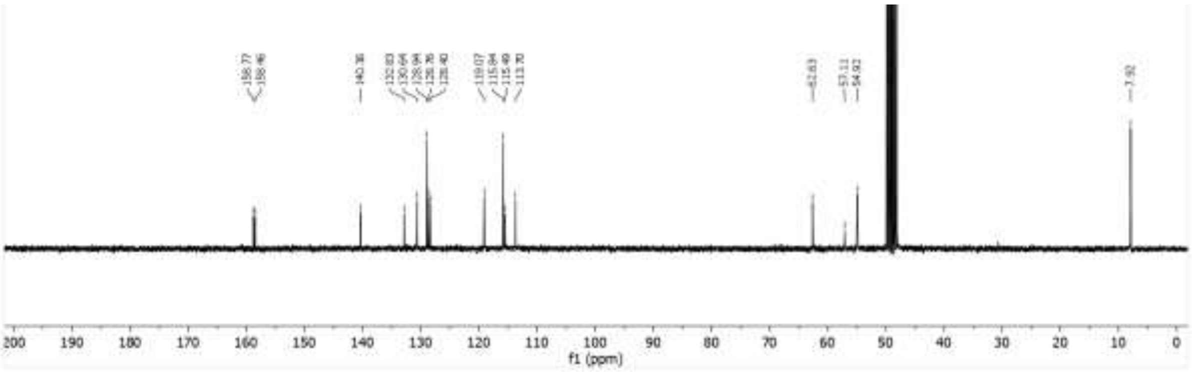

*(E)-N,N,N-triethyl-2-(4-(4-hydroxystyryl)phenoxy)ethan-1-aminium iodide (**20**).*

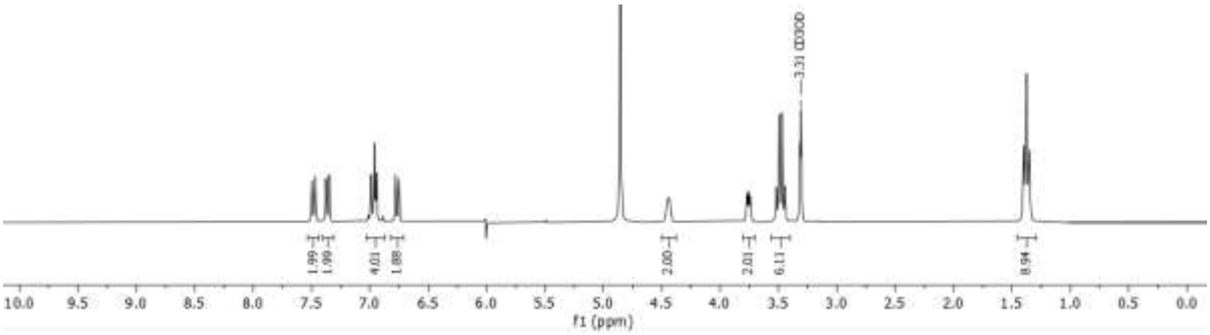

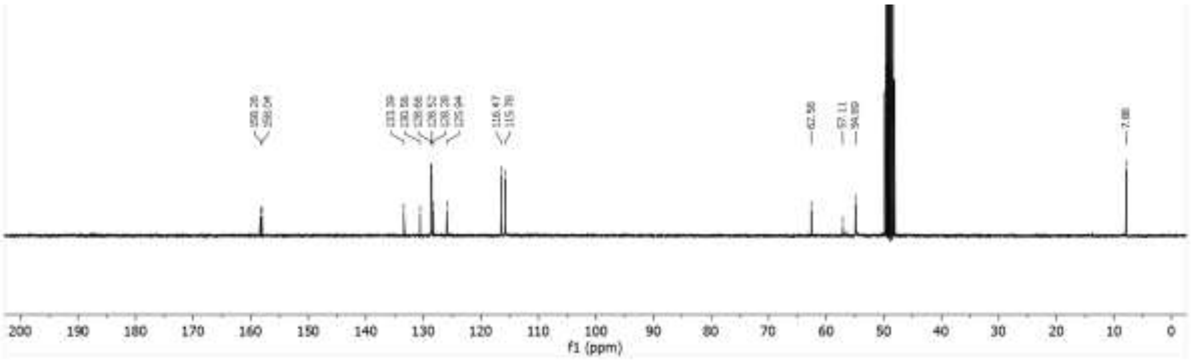

*(E)-2-(4-(3,5-dihydroxystyryl)phenoxy)-N,N,N-triethylethan-1-aminium iodide (**22**).*

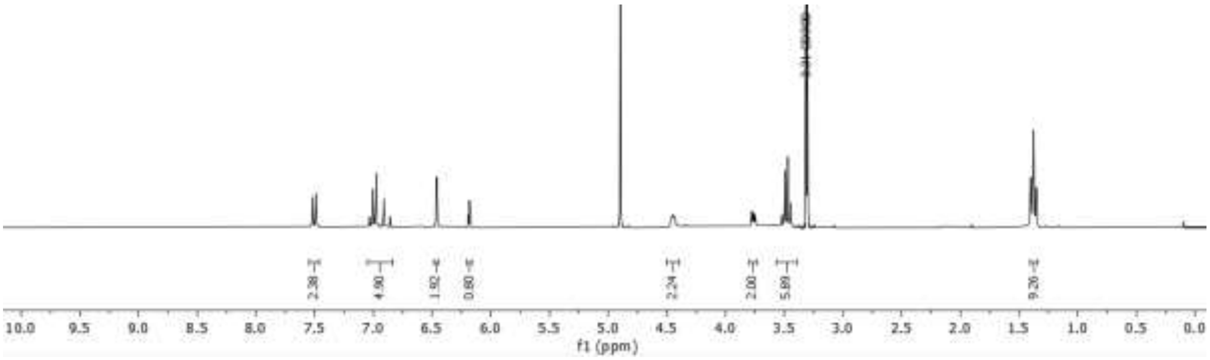

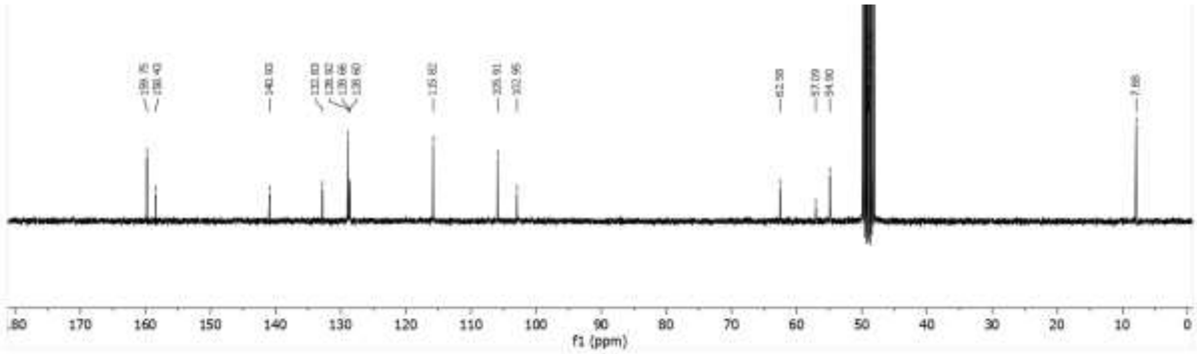

*2-(4-(benzyloxy)phenoxy)-N,N,N-triethylethan-1-aminium iodide (**23**).*

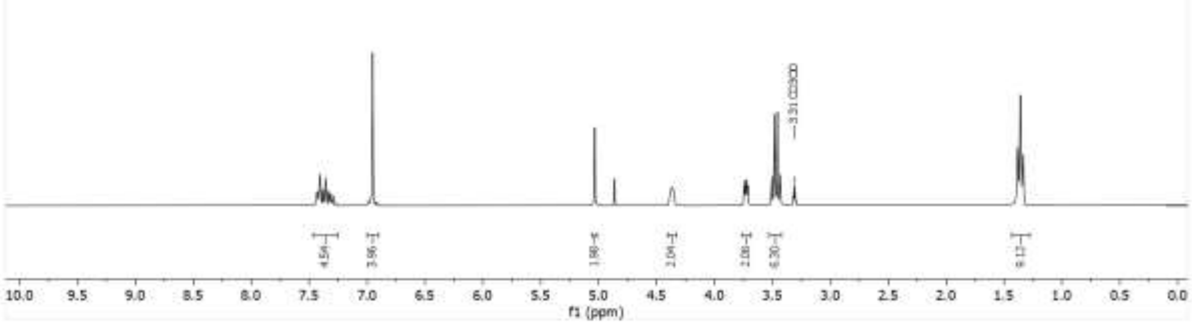

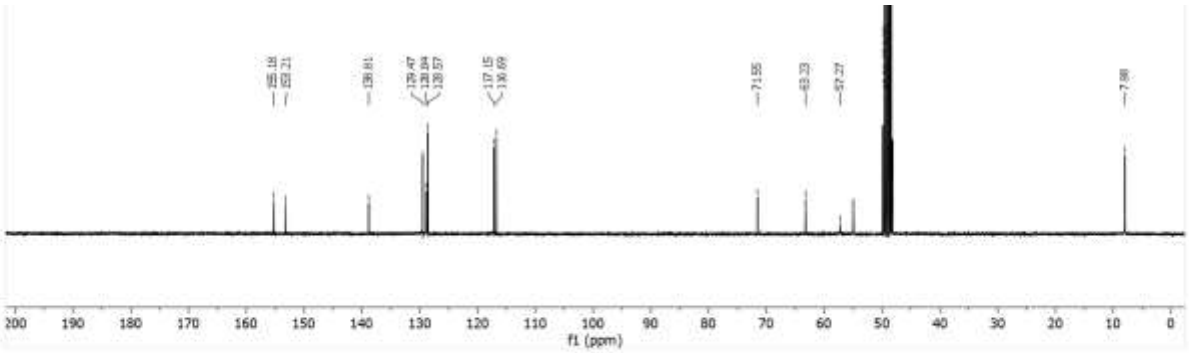

*N,N,N-triethyl-2-(4-(phenoxymethyl)phenoxy)ethan-1-aminium iodide (**24**).*

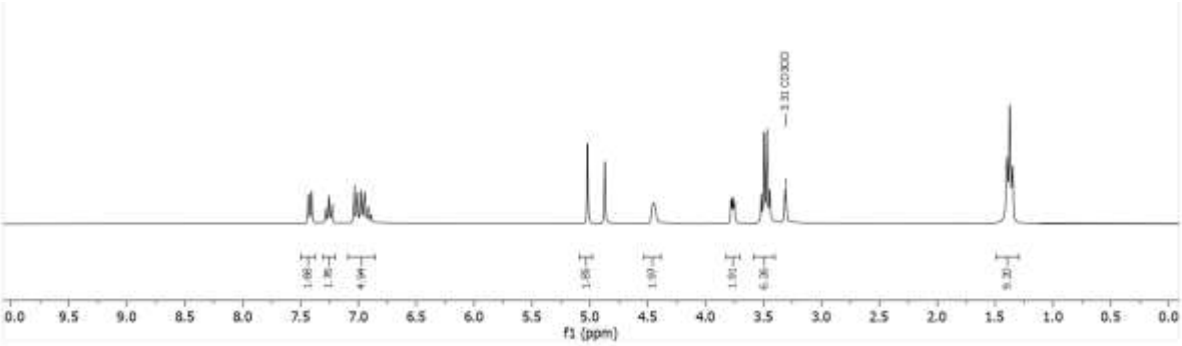

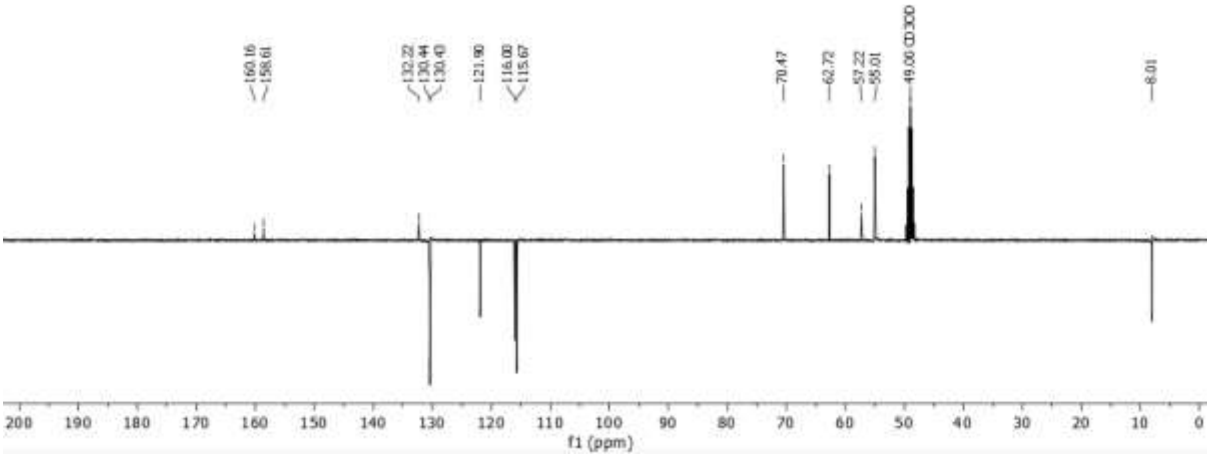

*2-(4-benzamidophenoxy)-N,N,N-triethylethan-1-aminium iodide (**25**).*

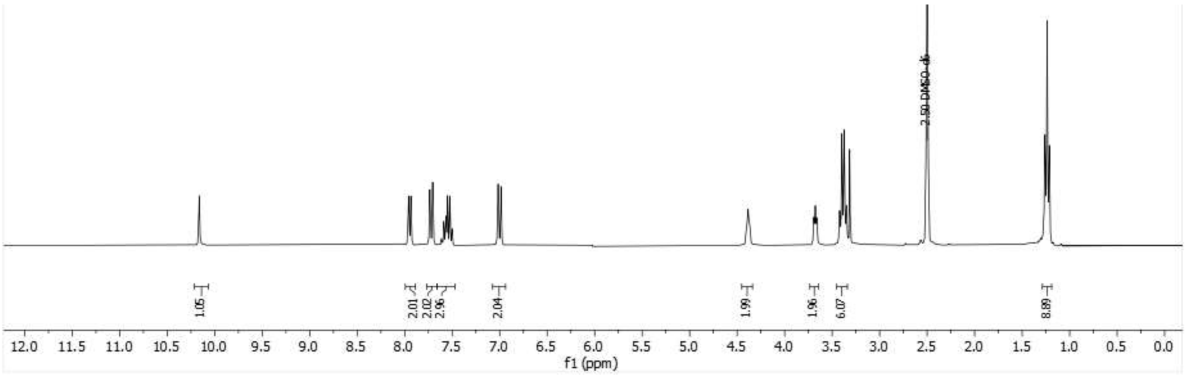

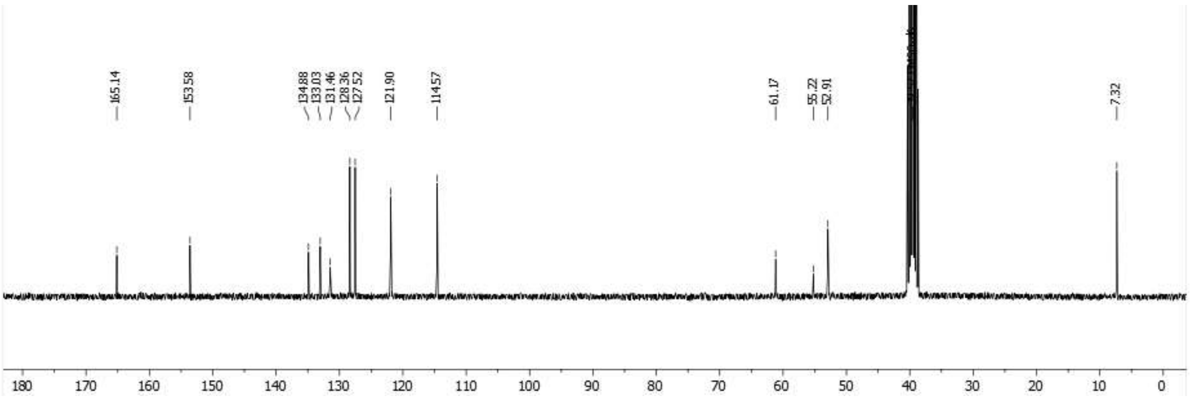

*N,N,N-triethyl-2-(4-(phenylcarbamoyl)phenoxy)ethan-1-aminium iodide (**26**).*

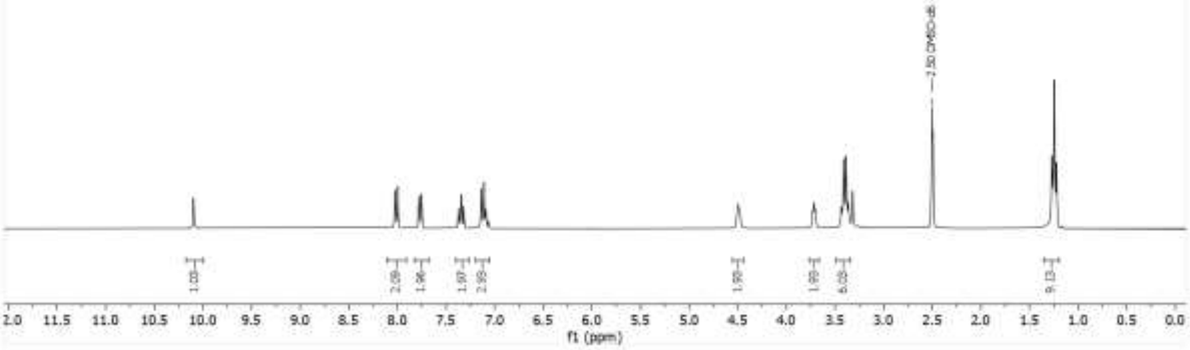

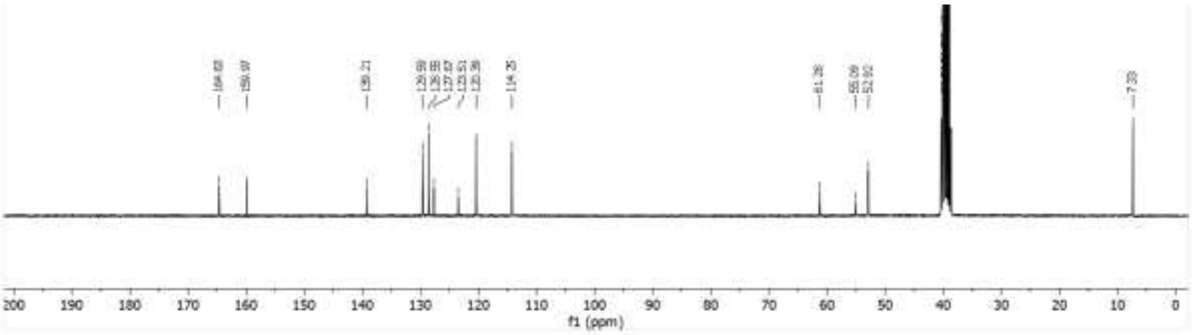

*(E)-N,N,N-triethyl-2-(4-(phenyldiazenyl)phenoxy)ethan-1-aminium iodide (**27**).*

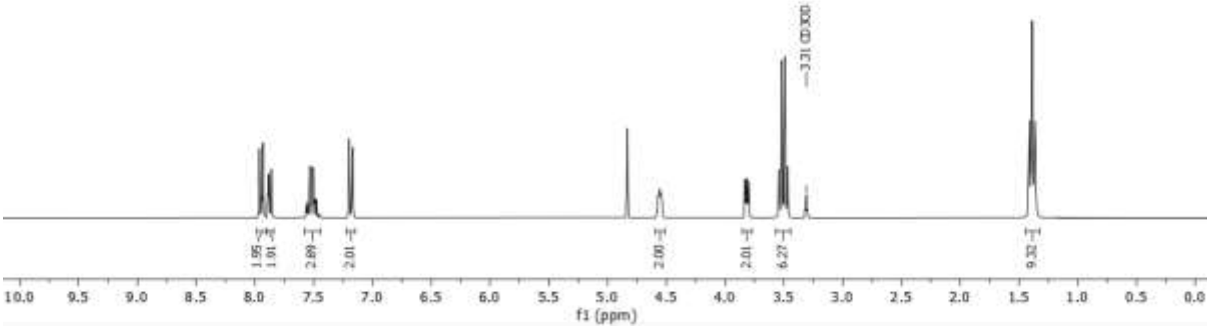

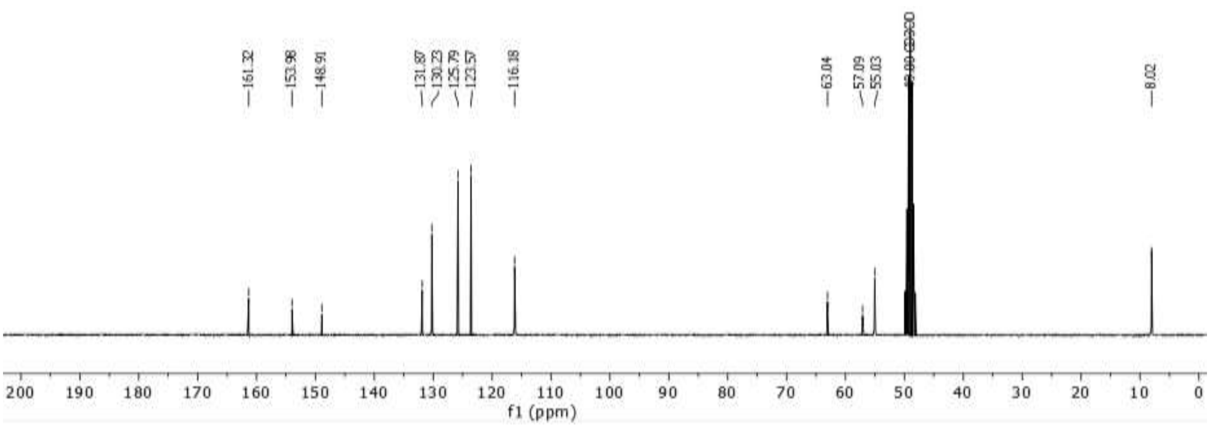

*2-(4-(benzo[d]oxazol-2-yl)phenoxy)-N,N,N-triethylethan-1-aminium iodide (**28**).*

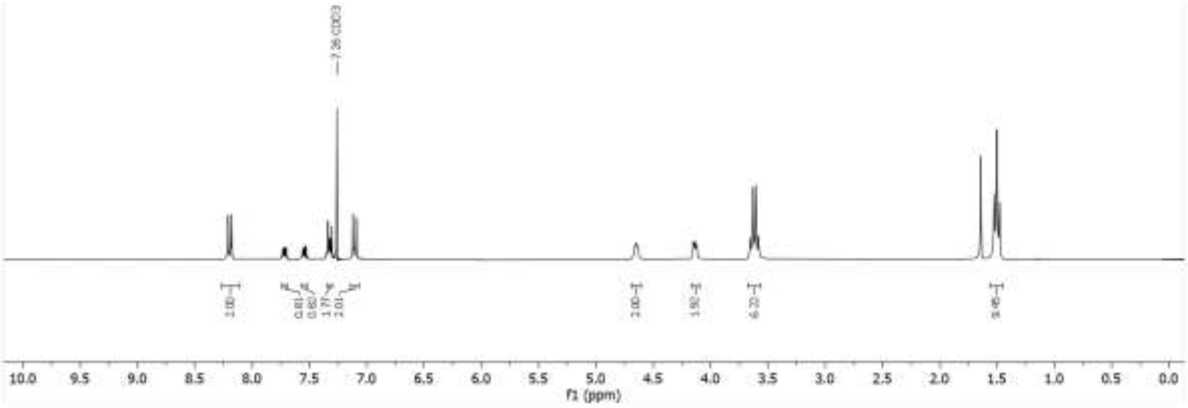

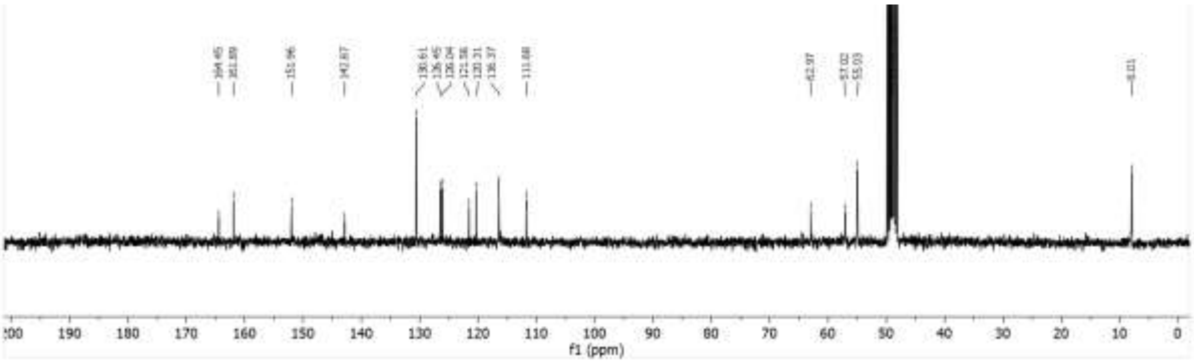

*2-(4-(1H-benzo[d]imidazol-2-yl)phenoxy)-N,N,N-triethylethan-1-aminium iodide (**29**).*

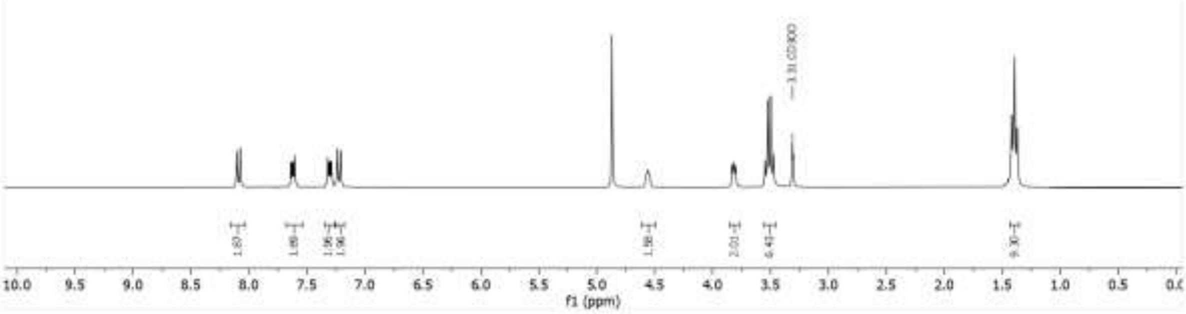

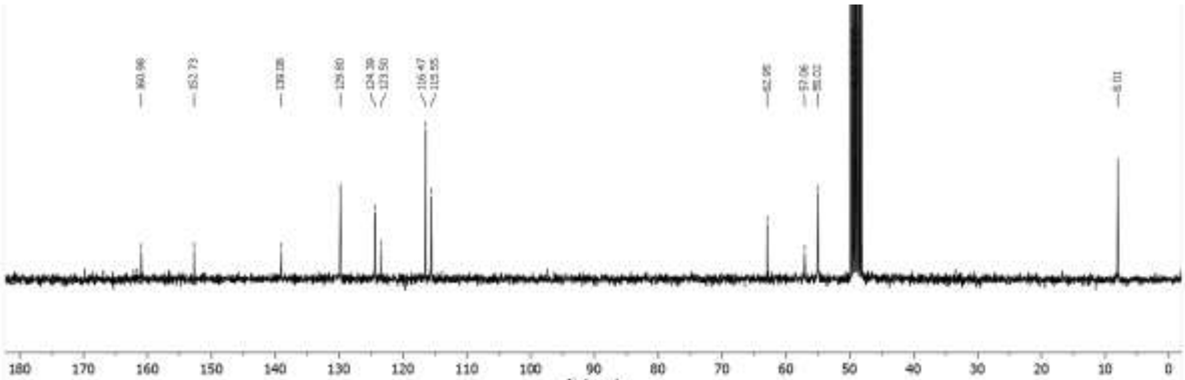

*2-(4-(1H-indol-6-yl)phenoxy)-N,N,N-triethylethan-1-aminium iodide (**30**).*

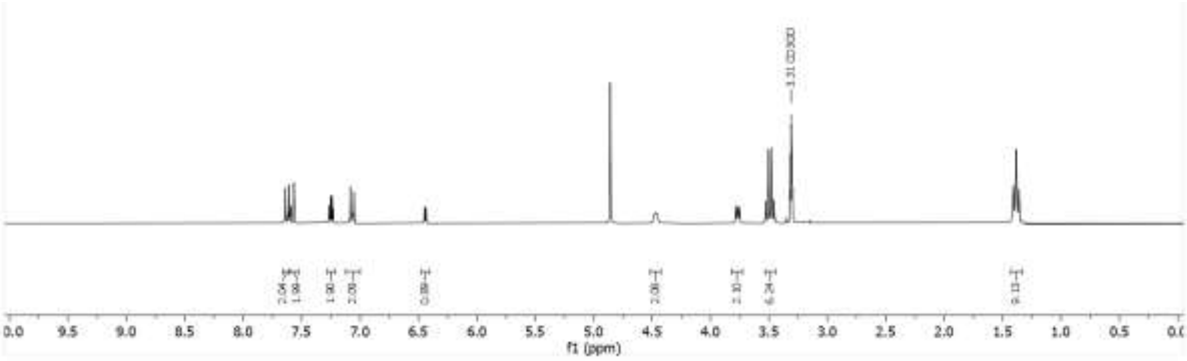

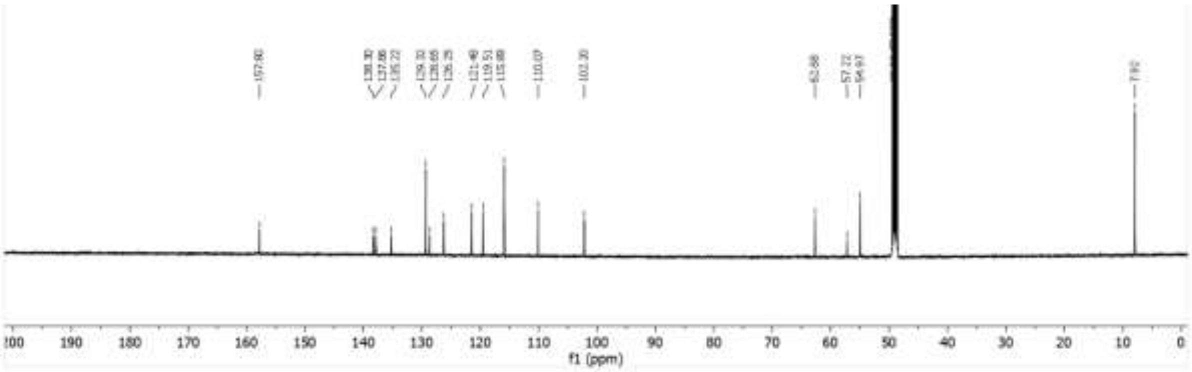

*2-(4-(1H-indol-5-yl)phenoxy)-N,N,N-triethylethan-1-aminium iodide (**31**).*

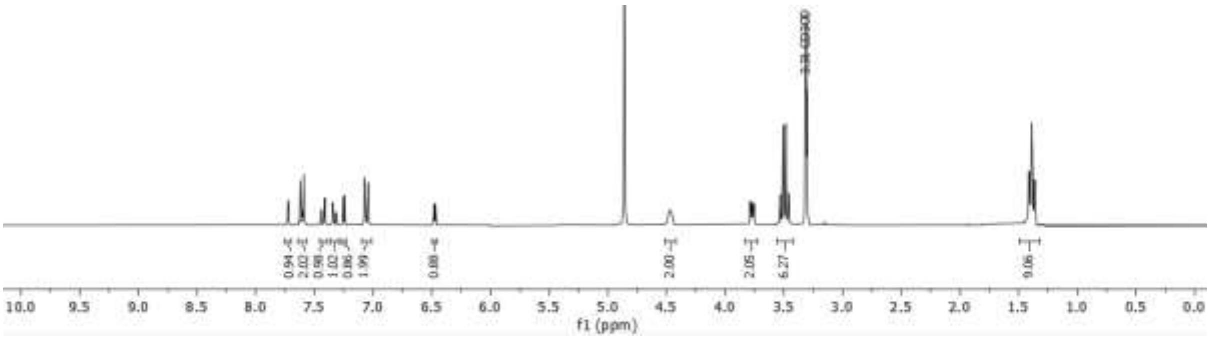

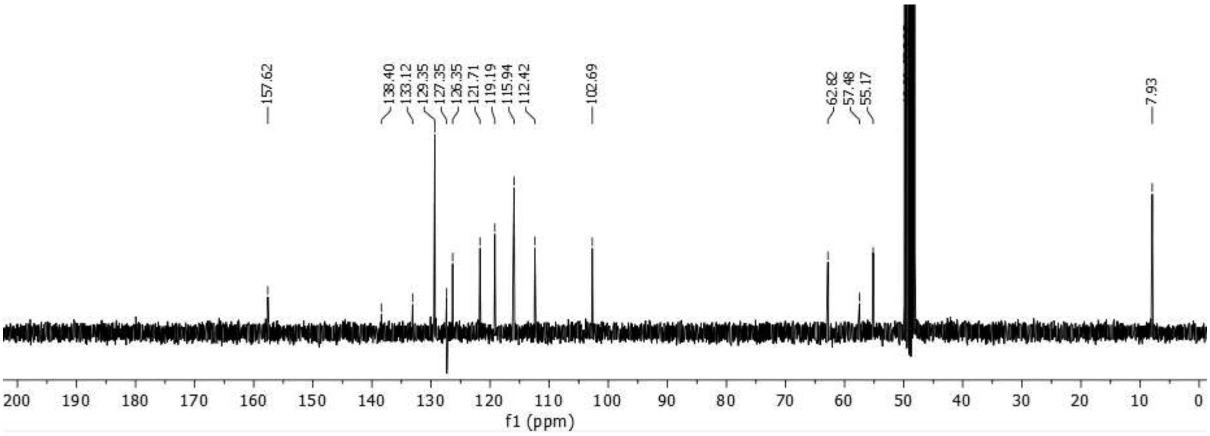

*2-(4-(benzofuran-5-yl)phenoxy)-N,N,N-triethylethan-1-aminium iodide (**32**).*

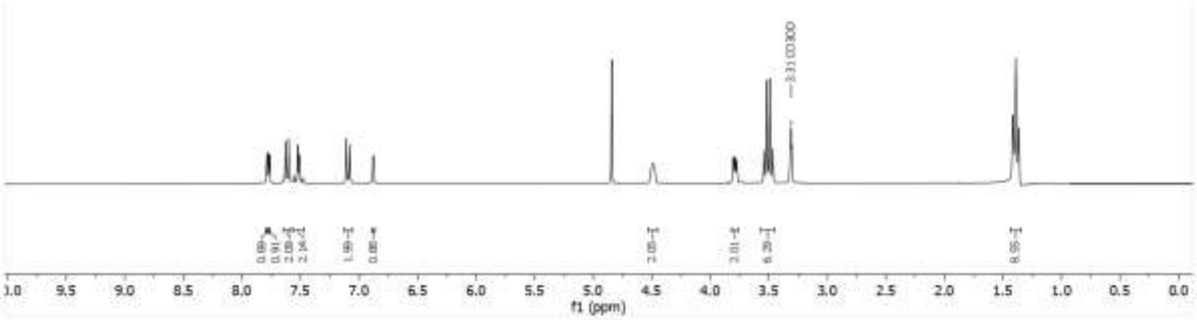

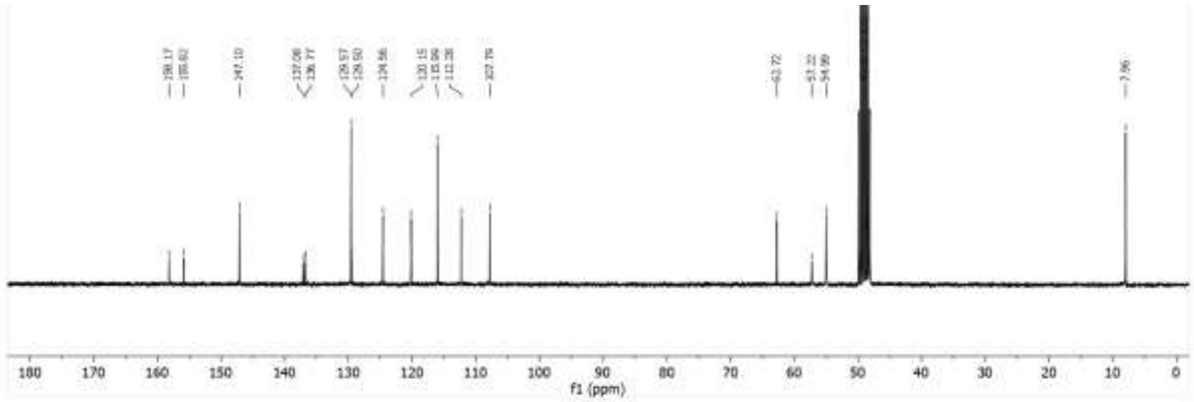

*(R)-3-(4-(1H-indol-5-yl)phenoxy)-1,1-dimethylpyrrolidin-1-ium iodide (**33**).*

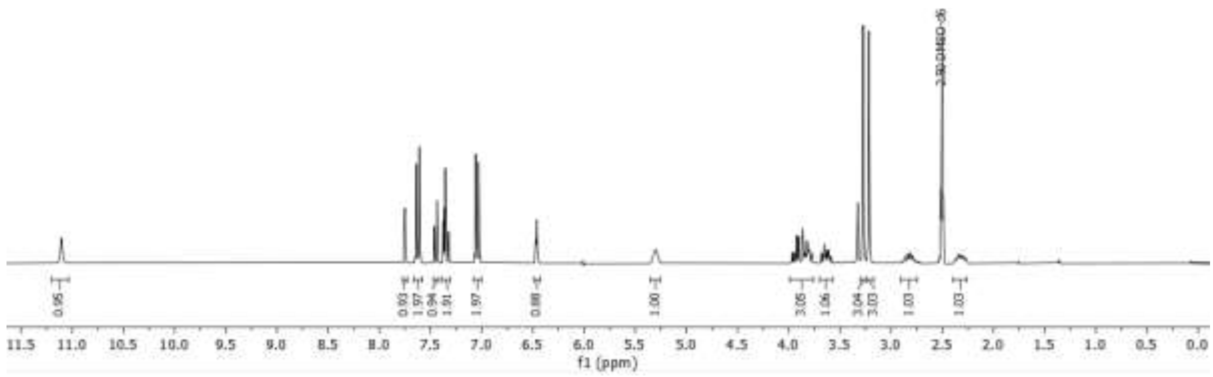

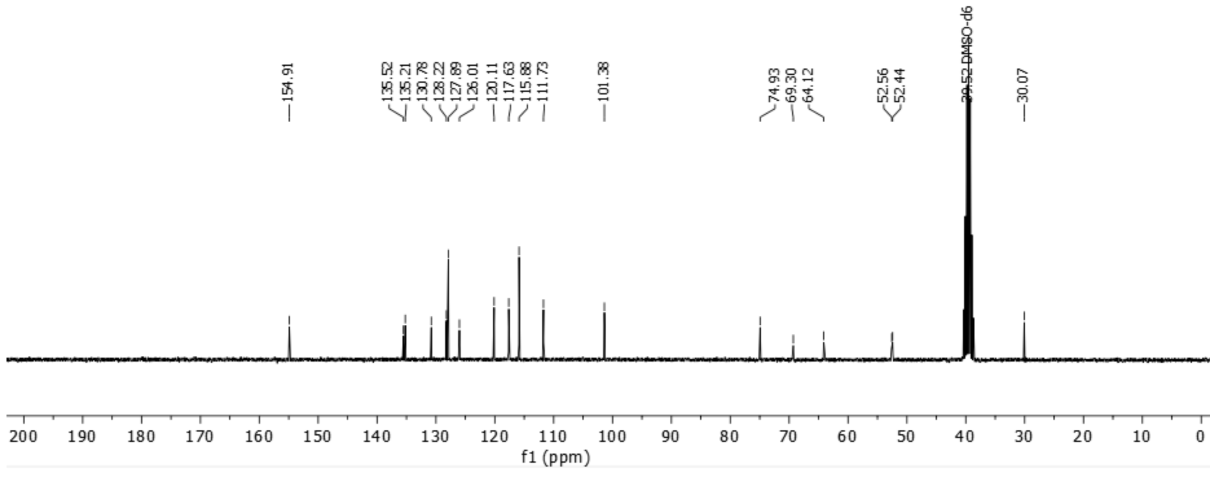

HPLC analysis of key final compounds

*N,N,N-triethyl-2-(4-phenethylphenoxy)ethan-1-aminium iodide (**6**).*

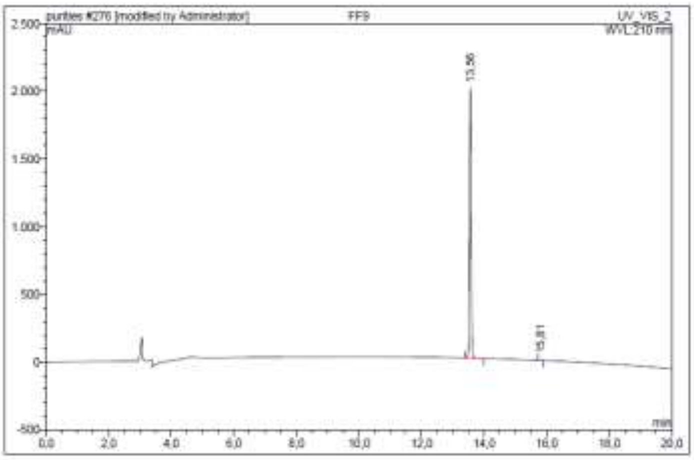

No.	Ret.Time min	Peak Name	Height mAU	Area mAU*min	Rel.Area %	Amount	Resolution(EP)
1	13,56	n.a.	1988,945	119,487	99,66	n.a.	23,22
2	15,81	n.a.	6,236	0,413	0,34	n.a.	n.a.
Total:			1995,181	119,900	100,00	0,000	

*2-(4-(benzo[d]oxazol-2-yl)phenoxy)-N,N,N-triethylethan-1-aminium iodide (**28**).*

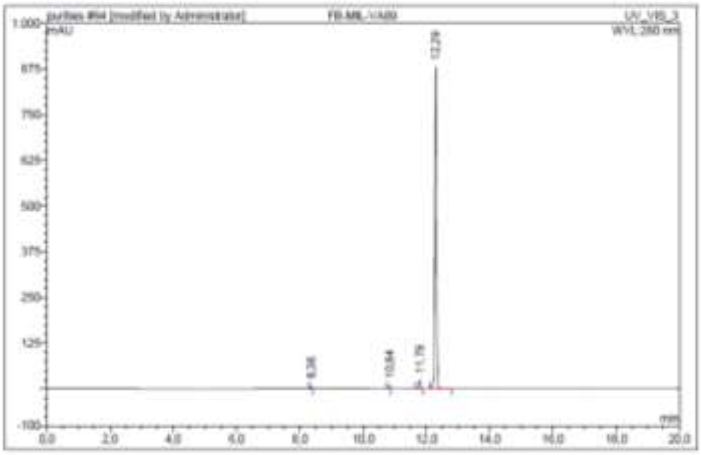

No.	Ret.Time min	Peak Name	Height mAU	Area mAU*min	Rel.Area %	Amount	Resolution(EP)
1	8,36	n.a.	8,725	0,454	0,84	n.a.	31,01
2	10,84	n.a.	8,684	0,404	0,75	n.a.	11,46
3	11,79	n.a.	23,501	1,328	2,47	n.a.	5,67
4	12,29	n.a.	880,381	51,699	95,94	n.a.	n.a.
Total:			921,291	53,886	100,00	0,000	

*(R)-3-(4-(1H-indol-5-yl)phenoxy)-1,1-dimethylpyrrolidin-1-ium iodide (**33**).*

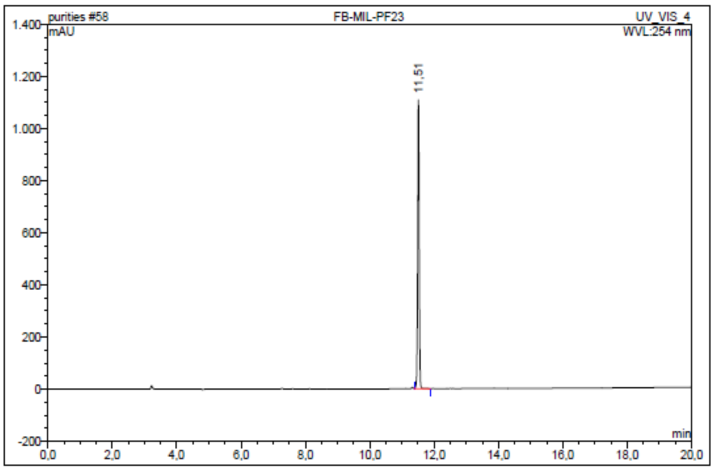

No.	Ret.Time min	Peak Name	Height mAU	Area mAU*min	Rel.Area %	Amount	Resolution(EP)
1	11,51	n.a.	1108,653	60,049	100,00	n.a.	n.a.
Total:			1108,653	60,049	100,00	0,000	

Example traces of two-electrode voltage-clamp recordings used to collect data for Figures 2 and 3.

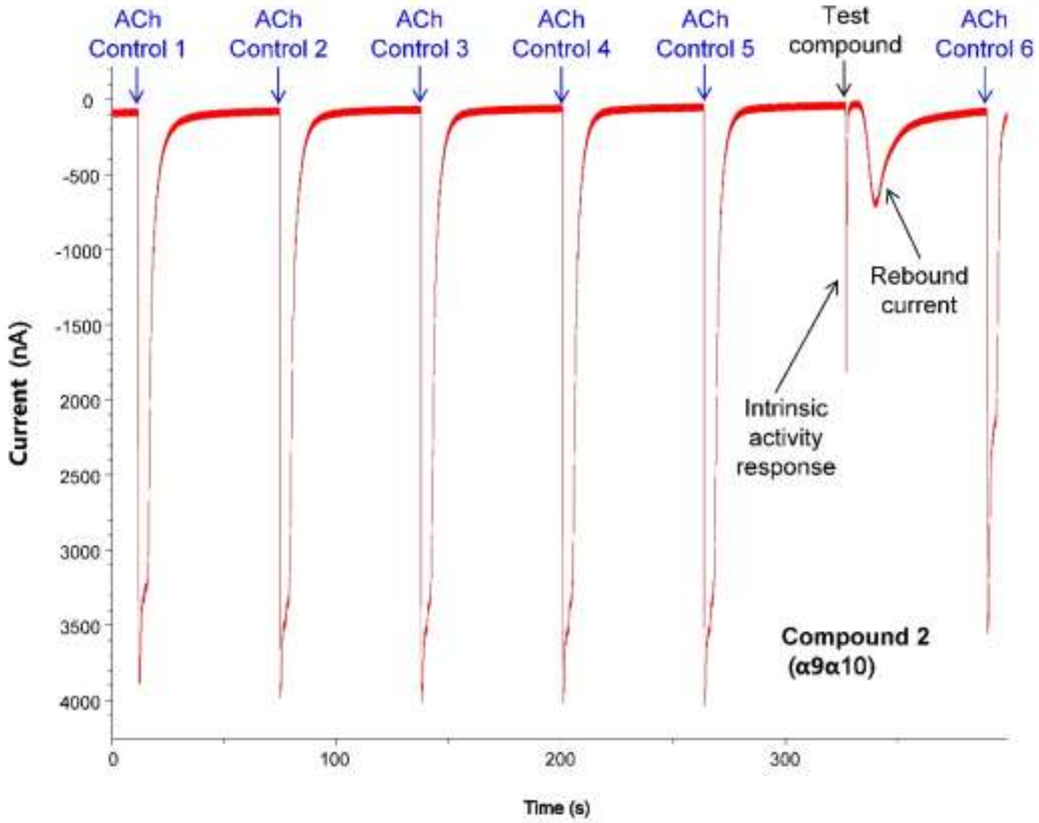

For illustrative purposes, timings of applications of ACh control and test compound applications are indicated in this case (for test Compound 2 at X. laevis oocytes expressing α9α10-nAChR). The very short initial response to test compound ("intrinsic activity response") and subsequent rebound current are indicated. For the remaining example recordings, identical application timings were used.

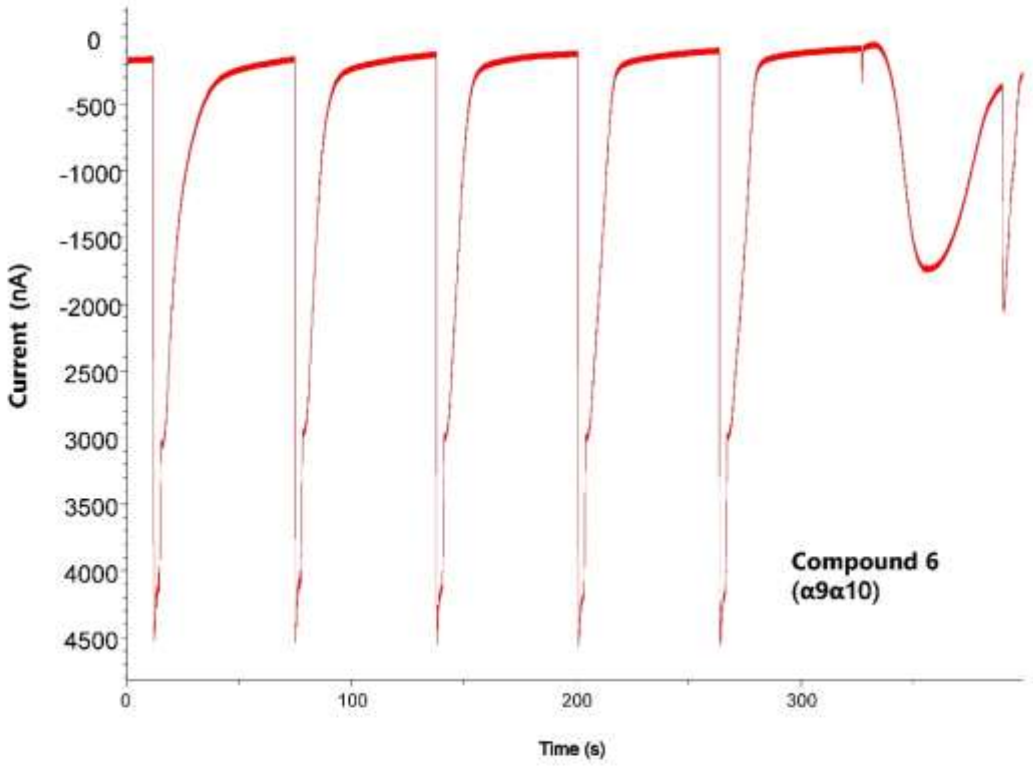

0
-500
-1000
-1500
-2000
2500
3000
3500
4000
4500
Current (nA)
0
100
200
300
Time (s)
Compound 6
(α9α10)

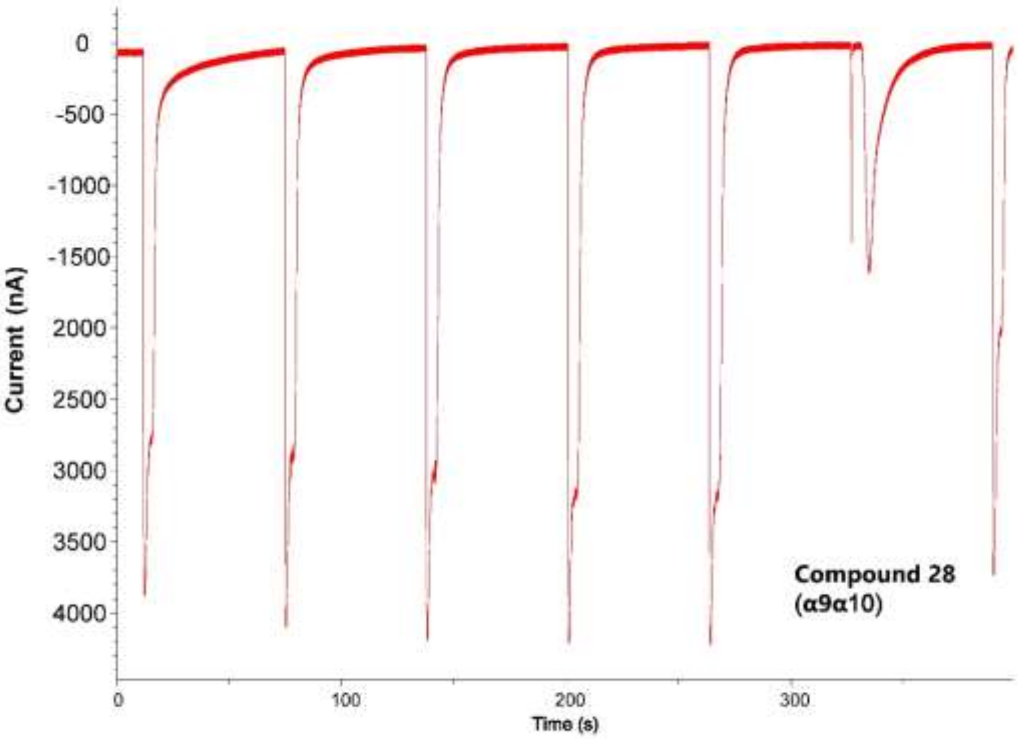

Current (nA)
0
-500
-1000
-1500
2000
2500
3000
3500
4000
0
100
200
300
Time (s)
Compound 28
(α9α10)

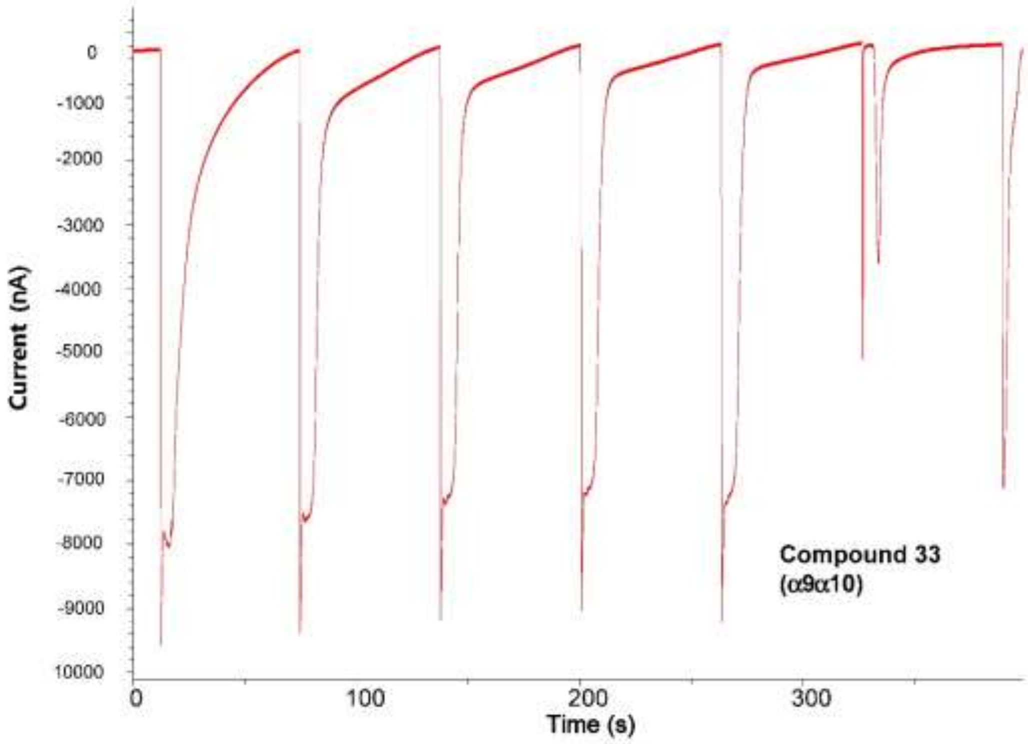
Current (nA)
0
-1000
-2000
-3000
-4000
-5000
-6000
-7000
-8000
-9000
10000
0
100
200
300
Time (s)
Compound 33
(α9α10)

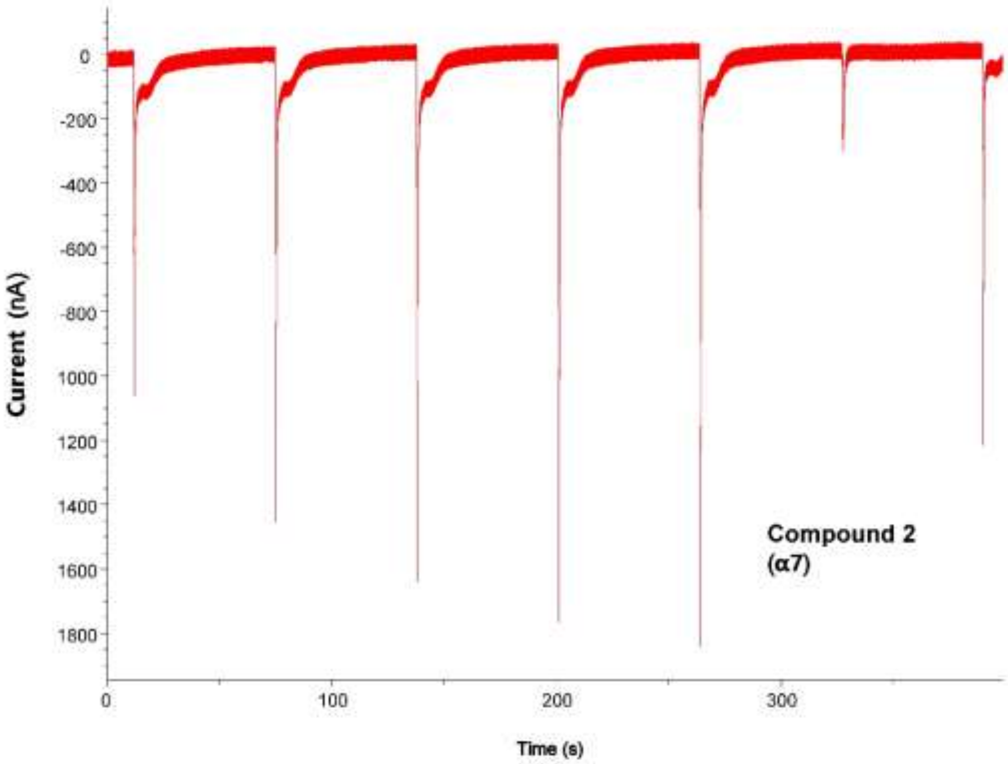

0
-200
-400
-600
-800
1000
1200
1400
1600
1800
Current (nA)
0
100
200
300
Time (s)
Compound 2
(α7)

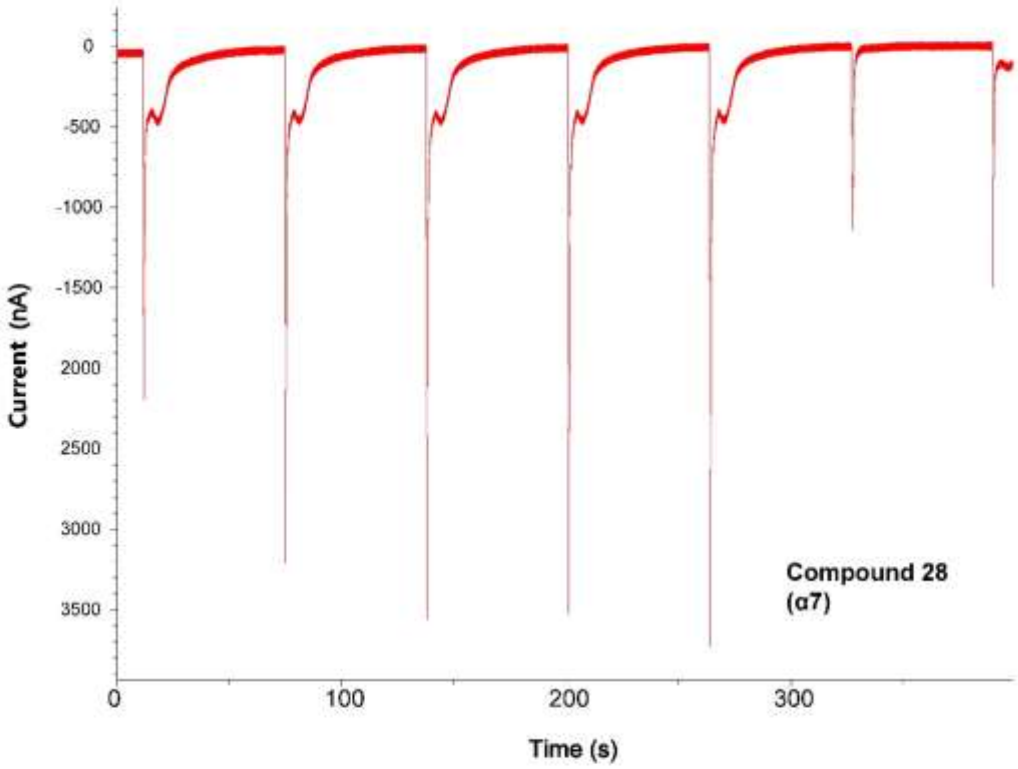
Current (nA)
0
-500
-1000
-1500
2000
2500
3000
3500
0
100
200
300
Time (s)
Compound 28
(α7)

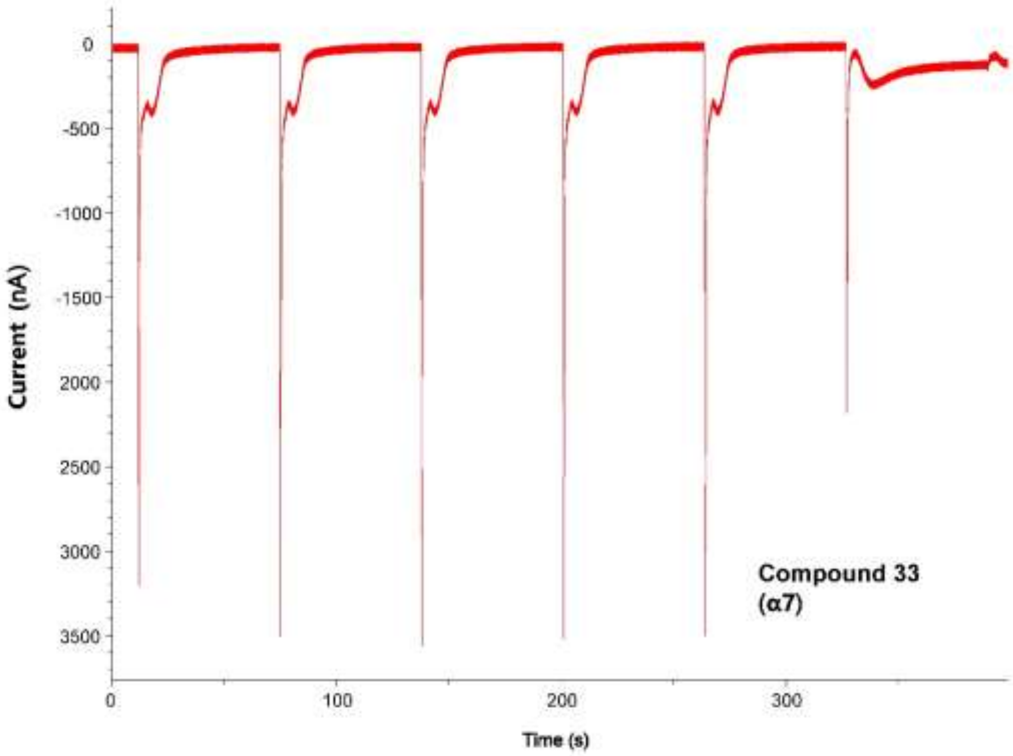

0
-500
-1000
-1500
2000
2500
3000
3500
Current (nA)
0
100
200
300
Time (s)
Compound 33
(α7)
